# Supplementary material for: Self-reported eating rate and metabolic syndrome in Japanese people: cross-sectional study
Source: BMJ Open. 2014 Sep 5;4(9):e005241. doi: 10.1136/bmjopen-2014-005241 (PMC4158192; doi:10.1136/bmjopen-2014-005241)
Supplement: Author's manuscript [file bmjopen-2014-005241.draft_revisions.pdf]

Eating rate and prevalence of metabolic syndrome in Japanese

Journal:	*BMJ Open*
Manuscript ID:	bmjopen-2014-005241
Article Type:	Research
Date Submitted by the Author:	11-Mar-2014
Complete List of Authors:	Nagahama, Satsue; All Japan Labor Welfare Foundation, ; School of Medicine, Toho University, Department of Environmental and Occupational Health<br>Kurotani, Kayo; National Center for Global Health and Medicine, Department of Epidemiology and Prevention, Center for Clinical Sciences<br>Pham, Ngoc Minh; Thai Nguyen University of Medicine and Pharmacy, Department of Epidemiology, Faculty of Public Health<br>Nanri, Akiko; National Center for Global Health and Medicine, Department of Epidemiology and Prevention, Center for Clinical Sciences<br>Kuwahara, Keisuke; National Center for Global Health and Medicine, Department of Epidemiology and Prevention, Center for Clinical Sciences<br>Dan, Masashi; All Japan Labor Welfare Foundation,<br>Nishiwaki, Yuji; School of Medicine, Toho University, Department of Environmental and Occupational Health<br>Mizoue, Tetsuya; National Center for Global Health and Medicine, Department of Epidemiology and Prevention, Center for Clinical Sciences
**Primary Subject Heading**:	Epidemiology
Secondary Subject Heading:	Epidemiology
Keywords:	EPIDEMIOLOGY, PREVENTIVE MEDICINE, PUBLIC HEALTH

SCHOLARONE™
Manuscripts

Eating rate and prevalence of metabolic syndrome in Japanese

Satsue Nagahama [1 2 3*], Kayo Kurotani [1], Ngoc Minh Pham [4], Akiko Nanri [1], Keisuke Kuwahara [1], Masashi Dan [2], Yuji Nishiwaki [3], Tetsuya Mizoue [1]

[1] Department of Epidemiology and Prevention, Center for Clinical Sciences, National Center for Global Health and Medicine, 1-21-1, Toyama, Shinjuku-ku, Tokyo, Japan, Zip162-8655; Tel +81-3-3202-7181, Fax 81-3-3202-7364, E-mail: satsue-n@umin.ac.jp

[2] All Japan Labor Welfare Foundation, Tokyo, Japan;

[3] Department of Environmental and Occupational Health, School of Medicine, Toho University, Tokyo, Japan;

[4] Department of Epidemiology, Faculty of Public Health, Thai Nguyen University of Medicine and Pharmacy, Thai Nguyen Province, Vietnam;

Keywords: eating rate, eating speed, metabolic syndrome, health check, Japan

Word count: 2354

For peer review only

For peer review only - http://bmjopen.bmj.com/site/about/guidelines.xhtml

ABSTRACT

Objectives: To examine the association between eating rate and the prevalence of metabolic syndrome.

Design: Cross-sectional study.

Setting: Annual health checkup for workers at a health check service center in Japan.

Participants: A total of 56,865 participants (41,820 males and 15,045 females) who attended health checkup in 2011 and reported not to have a history of coronary heart disease or stroke.

Main outcome measure: Metabolic syndrome was defined according to two criteria: the Adult Treatment Program III of the National Cholesterol Education Program (NCEP-ATPIII) and International Diabetes Federation (IDF).

Results: In multiple logistic regression models, eating rate was significantly and positively associated with metabolic syndrome defined by both criteria. The odds ratio (95% confidence interval) for slow, normal and fast were 0.70 (0.62 to 0.79), 1.00 (reference) and 1.61 (1.53 to 1.70), respectively in men (P for trend <0.001), and 0.74 (0.60 to 0.91), 1.00 (reference) and 1.27 (1.13 to 1.43), respectively, in women (P for trend <0.001) under NCEP-ATPIII criteria. The association of eating rate and metabolic syndrome was attenuated after further adjustment for body mass index, but remained

For peer review only

statistically significant only in men. Of components of metabolic syndrome, the strongest association with eating rate was abdominal obesity. The associations of slow eating with high blood pressure and hyperglycemia and those of fast eating with abnormal lipid profile were still significant in men, even after adjustment for body mass index.

Conclusions: Our results suggest that eating rate is associated with higher prevalence of metabolic syndrome and that this association is largely accounted for by higher body mass.

For peer review only

For peer review only - http://bmjopen.bmj.com/site/about/guidelines.xhtml

Strength and Limitation

This large scale study was the first study to elucidate the association between eating rate and metabolic syndrome in men and women, separately.

This is the only study to report the association between eating rate and metabolic syndrome defined by using waist circumference.

Eating rate was assessed by self-report questionnaire.

For peer review only

For peer review only - http://bmjopen.bmj.com/site/about/guidelines.xhtml

Introduction

Metabolic syndrome (MetS) is a cluster of physiological risk factors associated with cardiovascular disease and several types of cancer.[1] Determination of etiologic factors for MetS is required for the establishment of public health strategies to reduce its prevalence and prevent resulting complications. Growing evidence from both observational and interventional studies in human suggests a role of dietary habits in the development of MetS,[2-4] which originates from obesity. Obesity has been extensively investigated in relation to dietary habits including eating rate since 1962, when Ferster published a theoretical and practical weight control program focusing on eating behaviors including eating rate.[5] Observational studies showed that obese people ate at a faster rate than non-obese people,[6] and reducing eating rate may be a simple and effective therapy for obesity.[7]

During the past decade, several cross-sectional studies have found a positive association between eating rate and overweight[8-11] or insulin resistance.[11-15] Similarly, a few longitudinal studies showed that eating fast was associated with an increased risk of weight gain[16, 17] and type 2 diabetes.[18] In addition, some cross-sectional studies have reported that fast eating was positively associated with hypertriglyceridemia and low high-density lipoprotein cholesterol (HDL-C).[11, 14, 19] Therefore it is conceivable that

For peer review only - http://bmjopen.bmj.com/site/about/guidelines.xhtml

eating rate may be associated with the prevalence of MetS. To our knowledge, however, only one Korean study cross-sectionally examined eating rate in relation to MetS.[20] In that study, MetS was defined by using body mass index (BMI), rather than waist circumference, and investigated the association in men only. Waist circumference is a component of most MetS definitions[21, 22] as a surrogate of central obesity, which can better predict cardiovascular risk.[23] It is therefore necessary to examine the relationship between eating rate and MetS using waist circumference in both sexes. Here, we investigated cross-sectionally the association between eating rate and the prevalence of MetS according to two criteria: the National Cholesterol Education Program, Adult Treatment Panel III (NCEP-ATPIII) criteria[21] and International Diabetes Federation (IDF) criteria[22] using a large dataset of worker health checkup in Japanese men and women.

METHODS AND PROCEDURES

Study population

Study participants were attendants of 2011 (calendar year) annual health examination at All Japan Labor Welfare Foundation (Tokyo), a health service center performing health checkup for employees. Participants were employees and their

For peer review only - http://bmjopen.bmj.com/site/about/guidelines.xhtml

dependents, aged 17-99 in men and 17-85 in women. Of 297,148 participants, we excluded 3,660 with a history of myocardial infarction, coronary heart disease or stroke, 24,452 with missing information on eating rate, 192,581 who took meal within 8 hours or provided no information on meal time, 204,423 with missing data for any of the components of MetS, 15,886 with missing information on covariates (BMI, smoking status, alcohol consumption and physical activity). Some participants met more than one of these exclusion criteria, leaving 56,865 participants (41,820 males and 15,045 females) for analysis.

Data collection and measurements

In Japan, a new health checkup program focusing on MetS was initiated in 2008 according to the recommendation from the Ministry of Health, Labor and Welfare.[24] At All Japan Labor Welfare Foundation, a self-administered questionnaire, which was recommended by the Japanese government (Ministry of Health, Labor and Welfare),[25] was used to assess eating rate, medical history and health-related lifestyles including smoking, alcohol consumption and regular physical activity. Eating rate was assessed by asking "How fast is your speed of eating?", with three response options (slow, normal and fast). A trained staff measured height to the nearest 0.1 cm, weight to

For peer review only - http://bmjopen.bmj.com/site/about/guidelines.xhtml

the nearest 0.1 kg and waist circumference to the nearest 0.1 cm at the umbilical level in a standing position. BMI was calculated as the weight in kilograms divided by the squared height in meters. Blood pressure in the sitting position was measured using an automated machine (HEM-907, Omron, Kyoto, Japan). Participants with high blood pressure (≥130 mm Hg systolic or ≥85 mm Hg diastolic) received another measurement and data showing lower systolic blood pressure was used. Venous blood sample was collected, stored in a cooler at 4 degrees for transportation to an external laboratory (SRL, Tokyo, Japan) and measured within 24 hours of blood drawing. Triglyceride level was measured by enzymatic colorimertic test (Bio Majesty JCA-BM8060, JEOL, Tokyo, Japan). HDL-C level was determined by a direct method (Bio Majesty JCA-BM8060, JEOL, Tokyo, Japan). Plasma glucose levels were determined using by the hexokinase method (an automatic clinical chemistry analyzer JCA-BM9000 series, JEOL, Tokyo, Japan).

Definitions for MetS

MetS was defined according to two criteria: NCEP-ATPIII criteria and IDF criteria. Under NCEP-ATPIII criteria, MetS was defined as the presence of three or more of the following risk factors: 1) waist circumference for Asian population ≥90 cm

For peer review only - http://bmjopen.bmj.com/site/about/guidelines.xhtml

For peer review only

in men and ≥80 cm in women, 2) triglyceride level ≥150 mg/dL (1.7 mmol/L), 3) HDL-C level <40 mg/dL (1.04 mmol/L) in men and <50 mg/dL (1.3 mmol/L) in women, 4) blood pressure ≥130 mm Hg systolic or ≥85 mm Hg diastolic, 5) fasting glucose level ≥100 mg/dL (5.6 mmol/L).[21] To meet the criteria for IDF MetS, participants must have central obesity (waist circumference ≥90 cm in men and ≥80 cm in women) plus any two of the following factors: 1) HDL-C level <40 mg/dL (1.04 mmol/L) in men and <50 mg/dL (1.29 mmol/L) in women, 2) triglyceride level ≥150 mg/dL (1.7mmol/L), 3) blood pressure ≥130 mm Hg systolic or ≥85 mm Hg diastolic, 4) fasting glucose level ≥100 mg/dL (5.6 mmol/L).[22] In both criteria, participants under medication for diabetes, hypertension and dyslipidemia were considered as having respective factor, irrespective of measured data.

Statistical analysis

Participants were divided into three groups according to eating rate (slow, normal and fast). The characteristics of participants across eating rate categories were expressed as means (standard deviation), medians (interquartile range) and percentages for normal continuous variables, non-normal continuous variables and categorical variables, respectively. Trend association was assessed by assigning ordinal numbers (0

For peer review only

For peer review only - http://bmjopen.bmj.com/site/about/guidelines.xhtml

to 2) to the categories of eating rate (slow, normal and fast, respectively) and was tested using linear regression and an extension of the Wilcoxon Rank-Sum test for normal and non-normal continuous variables, respectively, and logistic regression for categorical variables. Multiple logistic regression analysis was used to estimate the odds ratios (OR) and 95% confidence interval (CI) for the prevalence of MetS across eating rate categories, with normal eating rate as the reference. In multiple regression analysis, the initial model was adjusted for age (continuous, year) only. In the second model, we further adjusted for smoking status (non-smoker, daily smoker consuming <20 cigarettes per day or ≥20 cigarettes per day), alcohol consumption (non-drinker, <1 *go*, 1 to <2 *go* or ≥2 *go* per day; *go* is a Japanese traditional unit of measurement for alcohol and a *go* of sake (Japanese traditional beverage) contains ~23g of ethanol) and physical activity (walking time <60 min per day or ≥60 min per day). In the third model, we added BMI (continuous, kg/m^2) to the second model. We performed likelihood ratio test for testing the interaction between eating rate and sex. All analyses were done for men and women separately because the interaction was significant (P for interaction <0.001). We repeated the above analyses for each component of MetS. Two-tailed P value <0.05 was considered statistically significant. All statistical analyses were performed with STATA, version 12.1 (StataCorp, College Station, TX, USA).

For peer review only - http://bmjopen.bmj.com/site/about/guidelines.xhtml

RESULTS

The prevalence of MetS defined by NCEP-ATPIII and IDF was 18.5% and 12.8%, respectively, in men and 12.9% and 11.4%, respectively, in women. **Table 1** shows characteristics of the study participants across categories of eating rate. Men who ate fast tended to be young, whereas women who ate slowly tended to be young. Those who ate fast had significantly higher BMI, waist circumference, triglyceride level and systolic and diastolic blood pressures and lower HDL-C level in both men and women.

The ORs of the prevalence of MetS across eating rate are shown in **Table 2**. Regardless of which criteria we used, faster eating was associated with higher prevalence of MetS in age- and multivariable-adjusted models. The trend was more apparent in men than in women under IDF criteria. The multivariable-adjusted ORs (95% CI) of MetS for eating slow, normal and fast rate were 0.70 (0.62 to 0.79), 1.00 (reference) and 1.61 (1.53 to 1.70), respectively, in men (P for trend <0.001), and 0.74 (0.60 to 0.91), 1.00 (reference) and 1.27 (1.13 to 1.43), respectively, in women (P for trend <0.001) under NCEP-ATPIII criteria; the correspondent values were 0.62 (0.54 to 0.72), 1.00 (reference) and 1.82 (1.72 to 1.94), respectively, in men (P for trend <0.001), and 0.73 (0.58 to 0.91), 1.00 (reference) and 1.32 (1.17 to 1.49), respectively, in women

For peer review only - http://bmjopen.bmj.com/site/about/guidelines.xhtml

(P for trend <0.001) under IDF criteria. Further adjustment for BMI markedly attenuated these associations; however, the association with eating rate and MetS under both criteria remained statistically significant in men.

Table 3 shows the ORs of the prevalence of individual MetS components across three categories of eating rate. The prevalence of central obesity increased from slow to fast eating in both men and women (OR 0.63, 1.00 (reference) and 1.97, respectively, in men (P for trend <0.001), and 0.73, 1.00(reference) and 1.44, respectively, in women (P for trend <0.001)). High blood pressure was positively associated with eating rate in both sexes. Eating rate was associated with fasting glucose, triglyceride and HDL-C in men and with HDL-C in women. Additional adjustment for BMI largely attenuated these associations, but the associations of slow eating with high blood pressure (men and women) and hyperglycemia (men) and those of fast eating with abnormal lipid profile (men) remained statistically significant.

DISCUSSION

In this large population of Japanese men and women, we found that eating rate was positively associated with the prevalence of MetS, especially in men. Of components of MetS, the association with abdominal obesity was strongest. The

For peer review only - http://bmjopen.bmj.com/site/about/guidelines.xhtml

relationship with blood pressure in both sexes and fasting plasma glucose and lipids in men remained statistically significant even after additional adjustment for BMI. To our best knowledge, the present study is the first to report a positive association between eating rate and the prevalence of MetS defined by using waist circumference.

The present finding for Mets is consistent with that of a study among Korean men reporting that eating rate was positively associated with MetS, which was defined by using BMI instead of waist circumference.[20] As regards MetS components, our study is compatible with some cross-sectional studies showing that eating rate was associated with higher BMI[8-10] and two longitudinal studies showing that eating rate was associated with weight gain.[16, 17] In a Korean study that elucidated the association between eating rate and components of MetS for men and women separately, eating rate was associated with obesity, high blood pressure, hyperglycemia and abnormal lipid profile in men, whereas it was associated with only obesity in women.[11] Our results were largely consistent with those in the Korean study (except for blood pressure in women).

We found that the association between eating rate and MetS was stronger in men than in women. In the present study, the association between eating rate and MetS components (hyperglycemia with slow eating and dyslipidemia with fast eating)

For peer review only - http://bmjopen.bmj.com/site/about/guidelines.xhtml

remained statistically significant even after adjusting for BMI in men but not in women. This finding appears to be compatible with those in previous studies showing sex difference in association of eating rate with MetS components[11] and insulin resistance.[12] These results may reflect the difference in actual eating speed between men and women. One study elucidated that women took more bites, smaller bite size and slower bites than men in eating the same amount of doughnut, irrespectively of body size.[26] Another study showed that objectively measured eating speed in men with self-reported slow eating was faster than that in women with self-reported fast eating.[27] Taken together, eating rate may have a greater impact on metabolism in men than that in women.

Although mechanisms whereby eating rate influences metabolism is not fully elucidated, overeating may link fast eating to MetS. Fast eating gives few satiety signal from oral cavity to the brain,[28, 29] induces less satiation and satiety due to a lack of stomach expansion[30] and alters the circulating levels of certain gut hormones.[31, 32] In these pathways, fast eating leads to excess energy intake,[33, 34] resulting in overweight and MetS. Because fast eating has been associated with obesity even after adjusting for total energy intake,[8-11, 14] there may be other pathways. One study showed that interleukin-1β and interleukin-6 were higher among those who ate fast than among those who ate slowly, even after accounting for energy intake and BMI.[35] The authors

For peer review only - http://bmjopen.bmj.com/site/about/guidelines.xhtml

ascribed the elevation of these inflammatory cytokines to an increased postprandial hyperglycemia after eating fast. These cytokines were well-known to give rise to obesity, dyslipidemia and insulin resistance,[36-39] and thus also accounted for the association between fast eating and MetS.

The strengths of our study deserve mention. Data of this large-scale study was derived from annual health-checkup for employees of various companies. Therefore, the present findings may be applicable to apparently healthy workers in Japan. In addition, body weight, body height and waist circumference was measured by trained technicians, which increased the validity of our study. Nonetheless, several limitations in the present study merit consideration. First, eating rate was self-reported. However, self-reported eating rate has been shown to be well correlated with friend-reported one[9] or objectively measured one.[27] Second, total energy intake was not available in our study. However, because energy intake is influenced by eating rate and thus may act as a mediator rather than confounder, the adjustment of energy intake could underestimate the association between eating rate and MetS. Moreover, eating rate has been associated with body weight independent of energy intake.[7-10, 13] Third, cross-sectional design precludes any causal inferences about the role of eating rate. Finally, we cannot exclude a possibility of the effects of residual confounding and confounding by unmeasured variables.

For peer review only - http://bmjopen.bmj.com/site/about/guidelines.xhtml

In conclusion, we found a positive trend association between eating rate and the prevalence of MetS, especially in men. The association between eating rate and MetS was largely accounted for by level of obesity, only eating rate per se may have small effect, in any, on the components of MetS. Further research should address whether reducing eating rate prevents obesity and MetS.

For peer review only

For peer review only - http://bmjopen.bmj.com/site/about/guidelines.xhtml

Table1. Characteristics of the study individuals according to eating rates

	Men (n = 41,820)			P for trend [a]	Women (n = 15,045)			P for trend [a]
	Slow	Normal	Fast		Slow	Normal	Fast	
n (%)	2,821 (6.8)	24,893 (59.5)	14,106 (33.7)		1,398 (9.3)	9,893 (65.8)	3,754 (24.9)	
Age (years) [b]	46.9 ± 12.3	46.9 ± 10.9	45.0 ± 10.4	<0.001	43.5 ± 12.5	47.2 ± 11.6	46.7 ± 11.2	<0.001
Walking time, ≥60 min/day (%)	21.8	19.0	20.6	0.004	15.5	15.0	16.1	0.798
Smoking status (%)								
Non-smoker	61.9	55.0	56.6	<0.001	82.9	83.1	80.7	0.572
Daily consuming <20 cigarettes /day	28.6	34.6	31.3		16.0	15.7	17.6	
Daily consuming ≥20 cigarettes /day	9.5	10.4	12.1		1.1	1.2	1.7	

For peer review only - http://bmjopen.bmj.com/site/about/guidelines.xhtml

For peer review only

Alcohol (%)								
Non-drinker	30.2	26.7	26.7	<0.001	53.4	52.2	49.8	<0.001
Drinker <1 *go* /day [d]	33.9	35.7	34.5		34.2	35.9	35.5	
Drinker 1 to <2 *go* /day [d]	24.6	26.3	26.8		9.5	9.5	11.5	
Drinker ≥2 *go* /day [d]	11.3	11.3	12.0		2.9	2.4	3.2	
BMI (kg/m^2)[b]	22.4 ± 3.3	23.4 ± 3.3	24.6 ± 3.7	<0.001	21.0 ± 3.5	21.8 ± 3.5	22.5 ± 3.8	<0.001
Waist circumference (cm)[b]	80.3 ± 9.2	82.9 ± 9.0	86.0 ± 9.8	<0.001	75.5 ± 9.5	77.7 ± 9.4	79.6 ± 9.8	<0.001
Systolic blood pressure (mm Hg)[b]	123.5 ± 15.5	126.1 ± 15.5	126.7 ± 15.1	<0.001	113.1 ± 16.3	117.3 ± 17.2	117.0 ± 17.2	<0.001
Diastolic blood pressure (mm Hg)[b]	75.2± 11.4	77.3 ± 11.9	78.0 ± 12.0	<0.001	69.1 ± 10.9	71.4 ± 11.5	71.5 ± 11.9	<0.001
Fasting plasma glucose (mg/dL)[c]	91 (85 to 98)	92 (86 to 99)	92 (86 to 99)	0.001	87 (82 to 93)	88 (83 to 94)	88 (83 to 94)	0.001
Triglyceride (mg/dL)[c]	92 (65 to 138)	99 (69 to 148)	107 (73 to 161)	<0.001	63 (48 to 87)	69 (51 to 96)	71 (52 to 101)	<0.001

For peer review only - http://bmjopen.bmj.com/site/about/guidelines.xhtml

For peer review only

HDL-C (mg/dL)[b]	61.3 ± 15.3	59.4 ± 15.0	57.2 ± 14.3	<0.001	71.4 ± 15.3	70.5 ± 15.8	69.3 ± 15.5	<0.001

Cross to sectional survey of 56,865 examinees in All Japan Labor Welfare Foundation, Japan, 2011.

BMI=body mass index; HDL-C=high to density lipoprotein cholesterol.

[a] Linear regression and an extension of the Wilcoxon Rank-Sum test for normal and non-normal continuous variables, respectively, and logistic regression for categorical variables, assigning ordinal number (0 to 2) to eating rate.

[b] Mean ± SD.

[c] Median (interquartile range).

[d] One *go* contains ~23g of ethanol.

For peer review only

For peer review only - http://bmjopen.bmj.com/site/about/guidelines.xhtml

Table2. Odds ratios and 95% confidence intervals for metabolic syndrome according to eating rate (n = 56,865)

Eating rate	Men (n = 41,820)			P for trend [a]	Women (n = 15,045)			P for trend [a]
	Slow	Normal	Fast		Slow	Normal	Fast	
n (%)	2,821 (6.8)	24,893 (59.5)	14,106 (33.7)		1,398 (9.3)	9,893 (65.8)	3,754 (25.0)	
NCEP-ATPIII								
MetS, n	361	4,180	3,193		116	1,261	547	
Model 1 [b]	0.70(0.62 to 0.79)	1.00 (Ref)	1.62(1.53 to 1.71)	<0.001	0.75(0.61 to 0.92)	1.00 (Ref)	1.27(1.13 to 1.42)	<0.001
Model 2 [c]	0.70(0.62 to 0.79)	1.00 (Ref)	1.61(1.53 to 1.70)	<0.001	0.74(0.60 to 0.91)	1.00 (Ref)	1.27(1.13 to 1.43)	<0.001
Model 3 [d]	0.91(0.80 to 1.04)	1.00 (Ref)	1.10(1.03 to 1.17)	<0.001	0.88(0.70 to 1.11)	1.00 (Ref)	0.98(0.86 to 1.12)	0.714
IDF								

For peer review only - http://bmjopen.bmj.com/site/about/guidelines.xhtml

For peer review only

MetS, n	205	2,727	2,450		101	1,115	504	
Model 1 [b]	0.62(0.54 to 0.72)	1.00 (Ref)	1.84(1.73 to 1.95)	<0.001	0.73(0.59 to 0.92)	1.00 (Ref)	1.32(1.17 to 1.49)	<0.001
Model 2 [c]	0.62(0.54 to 0.72)	1.00 (Ref)	1.82(1.72 to 1.94)	<0.001	0.73(0.58 to 0.91)	1.00 (Ref)	1.32(1.17 to 1.49)	<0.001
Model 3 [d]	0.81(0.67 to 0.97)	1.00 (Ref)	1.15(1.07 to 1.24)	<0.001	0.87(0.68 to 1.12)	1.00 (Ref)	1.00(0.87 to 1.14)	0.553

NCEP-ATPIII= the National Cholesterol Education Program, Adult Treatment Panel III: the presence of three or more of the following risk factors: 1) waist circumference ≥90 cm in men and ≥80 cm in women, 2) triglyceride level ≥150 mg/dL (1.7 mmol/L), 3) HDL-C level <40 mg/dL (1.04 mmol/L) in men and <50 mg/dL (1.3 mmol/L) in women, 4) blood pressure ≥130 mm Hg systolic or ≥85 mm Hg diastolic, 5) fasting glucose level ≥100 mg/dL (5.6 mmol/L); Ref=Reference; IDF= International Diabetes Federation: waist circumference ≥90 cm in men and ≥80 cm in women, and any two of the following factors: 1) HDL-C level <40 mg/dL (1.04 mmol/L) in men and <50 mg/dL (1.29 mmol/L) in women, 2) triglyceride level ≥150 mg/dL (1.7mmol/L), 3) blood pressure ≥130 mm Hg systolic or ≥85 mm Hg diastolic, 4) fasting glucose level ≥100 mg/dL (5.6 mmol/L).

[a] Multiple logistic regression, assigning ordinal number (0 to 2) to eating rate.

[b] Adjusted for age.

For peer review only

For peer review only - http://bmjopen.bmj.com/site/about/guidelines.xhtml

[c] Adjusted for age, smoking status, alcohol, and regular physical activity.

[d] Adjusted for age, smoking status, alcohol, regular physical activity, and body mass index.

For peer review only

For peer review only - http://bmjopen.bmj.com/site/about/guidelines.xhtml

Table3. Odds ratios and 95% confidence intervals for components of metabolic syndrome according to eating rate (n = 56,865)

	Men (n = 41,820)				Women (n = 15,045)			
Eating rate	slow	normal	fast	P for trend [a]	slow	normal	fast	P for trend [a]
n (%)	2,821 (6.8)	24,893 (59.5)	14,106 (33.7)		1,398 (9.3)	9,893 (65.8)	3,754 (24.9)	
Central obesity [b]								
Model 1 [c]	0.63(0.56 to 0.71)	1.00 (Ref)	1.98(1.89 to 2.08)	<0.001	0.73(0.64 to 0.83)	1.00 (Ref)	1.44(1.34 to 1.56)	<0.001
Model 2 [d]	0.63(0.56 to 0.70)	1.00 (Ref)	1.97(1.88 to 2.07)	<0.001	0.73(0.64 to 0.83)	1.00 (Ref)	1.44(1.33 to 1.56)	<0.001
High blood pressure [e]								
Model 1 [c]	0.75(0.69 to 0.82)	1.00 (Ref)	1.22(1.17 to 1.27)	<0.001	0.76(0.66 to 0.88)	1.00 (Ref)	1.10(1.01 to 1.21)	<0.001
Model 2 [d]	0.74(0.68 to 0.81)	1.00 (Ref)	1.20(1.15 to 1.26)	<0.001	0.76(0.65 to 0.88)	1.00 (Ref)	1.10(1.00 to 1.20)	<0.001

For peer review only - http://bmjopen.bmj.com/site/about/guidelines.xhtml

Model 3 [f]	0.88(0.81 to 0.96)	1.00 (Ref)	0.97(0.93 to 1.02)	0.645	0.85(0.72 to 0.99)	1.00 (Ref)	0.93(0.84 to 1.02)	0.923
High fasting plasma glucose [g]								
Model 1 [c]	0.78(0.71 to 0.87)	1.00 (Ref)	1.17(1.12 to 1.23)	<0.001	1.03(0.85 to 1.25)	1.00 (Ref)	1.17(1.04 to 1.31)	0.035
Model 2 [d]	0.78(0.71 to 0.87)	1.00 (Ref)	1.16(1.11 to 1.22)	<0.001	1.03(0.85 to 1.25)	1.00 (Ref)	1.16(1.03 to 1.31)	0.042
Model 3 [f]	0.88(0.80 to 0.98)	1.00 (Ref)	0.99(0.94 to 1.05)	0.238	1.14(0.94 to 1.40)	1.00 (Ref)	1.02(0.90 to 1.15)	0.536
High triglyceride [h]								
Model 1 [c]	0.88(0.80 to 0.96)	1.00 (Ref)	1.32(1.26 to 1.38)	<0.001	0.83(0.67 to 1.01)	1.00 (Ref)	1.14(1.01 to 1.28)	0.002
Model 2 [d]	0.90(0.82 to 0.98)	1.00 (Ref)	1.33(1.27 to 1.39)	<0.001	0.81(0.66 to 1.00)	1.00 (Ref)	1.13(1.01 to 1.27)	0.002
Model 3 [f]	1.08(0.98 to 1.19)	1.00 (Ref)	1.07(1.02 to 1.12)	0.121	0.90(0.73 to 1.11)	1.00 (Ref)	0.98(0.87 to 1.11)	0.753
Low HDL-C [i]								
Model 1 [c]	0.83(0.73 to 0.96)	1.00 (Ref)	1.34(1.26 to 1.43)	<0.001	0.90 (0.74 to 1.09)	1.00 (Ref)	1.11(0.99 to 1.25)	0.018

For peer review only - http://bmjopen.bmj.com/site/about/guidelines.xhtml

Model 2 [d]	0.83(0.73 to 0.96)	1.00 (Ref)	1.36(1.28 to 1.45)	<0.001	0.89(0.74 to 1.08)	1.00 (Ref)	1.12(1.00 to 1.26)	0.011
Model 3 [f]	0.97(0.84 to 1.12)	1.00 (Ref)	1.10(1.03 to 1.18)	0.004	1.00(0.82 to 1.22)	1.00 (Ref)	0.96(0.85 to 1.08)	0.500

Ref=Reference; BMI=body mass index; HDL-C=high to density lipoprotein cholesterol.

[a] Multiple logistic regression, assigning ordinal number (0 to 2) to eating rate.

[b] Waist circumference ≥ 90 cm in men, ≥80 cm in women.

[c] Adjusted for age.

[d] Adjusted for age, smoking status, alcohol, and regular physical activity.

[e] Blood pressure ≥ 130 mm Hg for systolic or ≥85 mm Hg for diastolic.

[f] Adjusted for age, smoking status, alcohol, regular physical activity and body mass index.

[g] Fasting plasma glucose ≥ 100 mg/dL or under medication.

[h] Triglyceride ≥150 mg/dL or under medication.

[i] HDL-C <40 mg/dL in men, <50 mg/dL in women or under medication.

For peer review only

For peer review only - http://bmjopen.bmj.com/site/about/guidelines.xhtml

Acknowledgments

The authors would like to thank Dr. Nobuo Yanagisawa and Dr. Takeshi Kawaguchi for their coordinating the study.

Contributors: SN and KKurotani designed study and drafted the manuscript. SN, NMP, AN, KKuwahara performed the data analysis. MD collected and interpreted the data. All authors have participated in the interpretation of the findings, revised it critically for important intellectual content and approved final version to be published. TM and YN provided administrative, technical and material support.SN and TM are guarantors.

Funding: This study was supported by the Industrial Health Foundation.

Competing interests: All authors have completed the ICMJE uniform disclosure form at www.icmje.org/coi_disclosure.pdf and declare: all authors declare no interests.

Ethical approval: The research protocol was approved by the Ethics Committee of the National Center for Global Health and Medicine and the Ethics Committee of Toho University.

Data sharing: No additional data available.

Transparency: SN affirms that the manuscript is an honest, accurate, and transparent account of the study being reported; that no important aspects of the study have been omitted; and that any discrepancies from the study as planned (and, if relevant,

For peer review only - http://bmjopen.bmj.com/site/about/guidelines.xhtml

For peer review only

registered) have been explained.

For peer review only

For peer review only - http://bmjopen.bmj.com/site/about/guidelines.xhtml

References

1. Eckel RH, Alberti KG, Grundy SM, et al. The metabolic syndrome. *Lancet* 2010;**375**:181-83.

2. Gomez-Martinez S, Martinez-Gomez D, Perez de Heredia F, et al. Eating habits and total and abdominal fat in Spanish adolescents: influence of physical activity. The AVENA study. *J Adolesc Health* 2012;**50**:403-09.

3. Ilanne-Parikka P, Eriksson JG, Lindstrom J, et al. Effect of lifestyle intervention on the occurrence of metabolic syndrome and its components in the Finnish Diabetes Prevention Study. *Diabetes care* 2008;**31**:805-07.

4. Nanri A, Tomita K, Matsushita Y, et al. Effect of six months lifestyle intervention in Japanese men with metabolic syndrome: randomized controlled trial. *J Occup Health* 2012;**54**:215-22.

5. Ferster CB, Nurnberger JI, Levitt EB. The control of eating. 1962. *Obes Res* 1996;**4**:401-10.

6. Hill SW, McCutcheon NB. Eating responses of obese and nonobese humans during dinner meals. *Psychosom Med* 1975;**37**:395-401.

7. Bellack AS. Behavior therapy for weight reduction. *Addict Behav* 1975;**1**:73-82.

8. Otsuka R, Tamakoshi K, Yatsuya H, et al. Eating fast leads to obesity: findings based

For peer review only

For peer review only - http://bmjopen.bmj.com/site/about/guidelines.xhtml

on self-administered questionnaires among middle-aged Japanese men and women. *J Epidemiol* 2006;**16**:117-24.

9. Sasaki S, Katagiri A, Tsuji T, et al. Self-reported rate of eating correlates with body mass index in 18-y-old Japanese women. *Int J Obes Relat Metab Disord* 2003;**27**:1405-10.

10. Maruyama K, Sato S, Ohira T, et al. The joint impact on being overweight of self reported behaviours of eating quickly and eating until full: cross sectional survey. *Bmj* 2008;**337**:a2002.

11. Lee KS, Kim DH, Jang JS, et al. Eating rate is associated with cardiometabolic risk factors in Korean adults. *Nut Metab Cardiovasc Dis* 2013;**23**:635-41.

12. Otsuka R, Tamakoshi K, Yatsuya H, et al. Eating fast leads to insulin resistance: findings in middle-aged Japanese men and women. *Prev Med* 2008;**46**:154-59.

13. Radzeviciene L, Ostrauskas R. Fast eating and the risk of type 2 diabetes mellitus: a case-control study. *Clin Nutr* 2013;**32**:232-35.

14. Ohkuma T, Fujii H, Iwase M, et al. Impact of eating rate on obesity and cardiovascular risk factors according to glucose tolerance status: the Fukuoka Diabetes Registry and the Hisayama Study. *Diabetologia* 2013;**56**:70-77.

15. Shigeta H, Shigeta M, Nakazawa A, et al. Lifestyle, obesity, and insulin resistance.

For peer review only - http://bmjopen.bmj.com/site/about/guidelines.xhtml

Diabetes care 2001;**24**:608.

16. Gerace TA, George VA. Predictors of weight increases over 7 years in fire fighters and paramedics. *Prev Med* 1996;**25**:593-600.

17. Tanihara S, Imatoh T, Miyazaki M, et al. Retrospective longitudinal study on the relationship between 8-year weight change and current eating speed. *Appetite* 2011;**57**:179-83.

18. Sakurai M, Nakamura K, Miura K, et al. Self-reported speed of eating and 7-year risk of type 2 diabetes mellitus in middle-aged Japanese men. *Metabolism* 2012;**61**:1566-71.

19. Kral JG, Buckley MC, Kissileff HR, et al. Metabolic correlates of eating behavior in severe obesity. *Int J Obes Relat Metab Disord* 2001;**25**:258-64.

20. Shin A, Lim SY, Sung J, et al. Dietary intake, eating habits, and metabolic syndrome in Korean men. *J Am Diet Assoc* 2009;**109**:633-40.

21. Grundy SM, Cleeman JI, Daniels SR, et al. Diagnosis and management of the metabolic syndrome: an American Heart Association/National Heart, Lung, and Blood Institute Scientific Statement. *Circulation* 2005;**112**:2735-52.

22. Alberti KG, Zimmet P, Shaw J. Metabolic syndrome--a new world-wide definition. A Consensus Statement from the International Diabetes Federation. *Diabet Med*

For peer review only

For peer review only - http://bmjopen.bmj.com/site/about/guidelines.xhtml

2006;23:469-80.

23. Shen W, Punyanitya M, Chen J, et al. Waist circumference correlates with metabolic syndrome indicators better than percentage fat. *Obesity (Silver Spring)* 2006;**14**:727-36.

24. Questionnaire of Specific Health Checkups. Secondary Questionnaire of Specific Health Checkups. Available at: http://www.mhlw.go.jp/english/wp/wp-hw3/dl/2-007.pdf. Accessed 21 Feb 2014.

25. Specific Health Checkups and Specific Health Guidance. Secondary Specific Health Checkups and Specific Health Guidance. Available at: http://www.mhlw.go.jp/seisakunitsuite/bunya/kenkou_iryou/kenkou/seikatsu/dl/hoken-program2_02.pdf. Accessed 21 Feb 2014.

26. Hill SW, McCutcheon NB. Contributions of obesity, gender, hunger, food preference, and body size to bite size, bite speed, and rate of eating. *Appetite* 1984;**5**:73-83.

27. Ekuni D, Furuta M, Takeuchi N, et al. Self-reports of eating quickly are related to a decreased number of chews until first swallow, total number of chews, and total duration of chewing in young people. *Arch Oral Biol* 2012;**57**:981-86.

28. Rolls ET. Sensory processing in the brain related to the control of food intake. *Proc Nutr Soc* 2007;**66**:96-112.

29. Morton GJ, Cummings DE, Baskin DG, et al. Central nervous system control of

For peer review only

food intake and body weight. *Nature* 2006;**443**:289-95.

30. de Graaf C, Blom WA, Smeets PA, et al. Biomarkers of satiation and satiety. *Am J Clin Nutr* 2004;**79**:946-61.

31. Kokkinos A, le Roux CW, Alexiadou K, et al. Eating slowly increases the postprandial response of the anorexigenic gut hormones, peptide YY and glucagon-like peptide-1. *J Clin Endocrinol Metab* 2010;**95**:333-37.

32. Sobki SH, Zaid AA, Khan HA, et al. Significant impact of pace of eating on serum ghrelin and glucose levels. *Clin Biochem* 2010;**43**:522-24.

33. Viskaal-van Dongen M, Kok FJ, de Graaf C. Eating rate of commonly consumed foods promotes food and energy intake. *Appetite* 2011;**56**:25-31.

34. Scisco JL, Muth ER, Dong Y, et al. Slowing bite-rate reduces energy intake: an application of the bite counter device. *J Am Diet Assoc* 2011;**111**:1231-35.

35. Mochizuki K, Misaki Y, Miyauchi R, et al. A higher rate of eating is associated with higher circulating interluekin-1beta concentrations in Japanese men not being treated for metabolic diseases. *Nutrition* 2012;**28**:978-83.

36. Besedovsky H, del Rey A, Sorkin E, et al. Immunoregulatory feedback between interleukin-1 and glucocorticoid hormones. *Science* 1986;**233**:652-54

37. Matsuki T, Horai R, Sudo K, et al. IL-1 plays an important role in lipid metabolism

For peer review only

by regulating insulin levels under physiological conditions. *J Exp Med* 2003;**198**:877-88.

38. Speaker KJ, Fleshner M. Interleukin-1 beta: a potential link between stress and the development of visceral obesity. *BMC physiol* 2012;**27**:8.

39. Thorand B, Baumert J, Doring A, et al. Sex differences in the relation of body composition to markers of inflammation. *Atherosclerosis* 2006;**184**:216-24.

For peer review only

For peer review only - http://bmjopen.bmj.com/site/about/guidelines.xhtml

STROBE Statement—Checklist of items that should be included in reports of ***cross-sectional studies***

MS ID#: BMJ.2014.018520
MS TITLE: Eating rate and prevalence of metabolic syndrome in Japanese.

	Item No	Recommendation	Location in manuscript
**Title and abstract**	1	(*a*) Indicate the study's design with a commonly used term in the title or the abstract	Line 20 on page 2
		(*b*) Provide in the abstract an informative and balanced summary of what was done and what was found	What was done: line 18-19 on page 2<br>What was found: line 40-42 on page 2
**Introduction**			
Background/rationale	2	Explain the scientific background and rationale for the investigation being reported	Scientific background: line 50-73 on page 5<br>Rationale: Line 73-78 on page 5-6
Objectives	3	State specific objectives, including any prespecified hypotheses	Line 67-68 and line 77-78 on page 6
**Methods**			
Study design	4	Present key elements of study design early in the paper	Line 74 on page 6
Setting	5	Describe the setting, locations, and relevant dates, including periods of recruitment, exposure, follow-up, and data collection	Line 82-85 on page 6-7
Participants	6	(*a*) Give the eligibility criteria, and the sources and methods of selection of participants	Line 85-92 on page 7
Variables	7	Clearly define all outcomes, exposures, predictors, potential confounders, and effect modifiers. Give diagnostic criteria, if applicable	Line 118-150 on page 8-10
Data sources/ measurement	8*	For each variable of interest, give sources of data and details of methods of assessment (measurement). Describe comparability of assessment methods if there is more than one group	Line 100-102 on page 7
Bias	9	Describe any efforts to address potential sources of bias	Exclusion: line 85-92 on page 7<br>Adjustment: line 145-151 on page 10
Study size	10	Explain how the study size was arrived at	Not provided
Quantitative variables	11	Explain how quantitative variables were handled in the analyses. If applicable, describe which groupings were chosen and why	Line 144-151 on page 10

For peer review only - http://bmjopen.bmj.com/site/about/guidelines.xhtml

Statistical methods	12	(*a*) Describe all statistical methods, including those used to control for confounding	Line 134-156 on page 9-10
		(*b*) Describe any methods used to examine subgroups and interactions	Line 151-153 on page 10
		(*c*) Explain how missing data were addressed	We excluded participants who had missing information on potential confounding variables (line 89 on page 7).
		(*d*) If applicable, describe analytical methods taking account of sampling strategy	N/A
		(*e*) Describe any sensitivity analyses	N/A
**Results**			
Participants	13*	(a) Report numbers of individuals at each stage of study—eg numbers potentially eligible, examined for eligibility, confirmed eligible, included in the study, completing follow-up, and analysed	Line 159-160 on page 11<br>Table 1
		(b) Give reasons for non-participation at each stage	N/A
		(c) Consider use of a flow diagram	N/A
Descriptive data	14*	(a) Give characteristics of study participants (eg demographic, clinical, social) and information on exposures and potential confounders	Table 1<br>Line 159-164 on page 11
		(b) Indicate number of participants with missing data for each variable of interest	N/A
Outcome data	15*	Report numbers of outcome events or summary measures	Table 2<br>Line 159-160 on page 11
Main results	16	(*a*) Give unadjusted estimates and, if applicable, confounder-adjusted estimates and their precision (eg, 95% confidence interval). Make clear which confounders were adjusted for and why they were included	Table 2, Table3<br>Line 165-187 on page 11-12
		(*b*) Report category boundaries when continuous variables were categorized	Table2, Table3
		(*c*) If relevant, consider translating estimates of relative risk into absolute risk for a meaningful time period	N/A
Other analyses	17	Report other analyses done—eg analyses of subgroups and interactions, and sensitivity analyses	Line 152-153 on page 10
**Discussion**			
Key results	18	Summarise key results with reference to study objectives	Line 190-194 on page 12-13
Limitations	19	Discuss limitations of the study, taking into account sources of potential bias	Line 237-246 on page 15

For peer review only - http://bmjopen.bmj.com/site/about/guidelines.xhtml

		or imprecision. Discuss both direction and magnitude of any potential bias	
Interpretation	20	Give a cautious overall interpretation of results considering objectives, limitations, multiplicity of analyses, results from similar studies, and other relevant evidence	Line 197-219 on page 13-14
Generalisability	21	Discuss the generalisability (external validity) of the study results	Line 247-251 on page 16
**Other information**			
Funding	22	Give the source of funding and the role of the funders for the present study and, if applicable, for the original study on which the present article is based	This study was supported by the Industrial Health Foundation for drafting the maniscript.

N/A: Not applicable.

*Give information separately for exposed and unexposed groups.

Note: An Explanation and Elaboration article discusses each checklist item and gives methodological background and published examples of transparent reporting. The STROBE checklist is best used in conjunction with this article (freely available on the Web sites of PLoS Medicine at http://www.plosmedicine.org/, Annals of Internal Medicine at http://www.annals.org/, and Epidemiology at http://www.epidem.com/). Information on the STROBE Initiative is available at www.strobe-statement.org.

For peer review only

BMJ Open

Self-reported eating rate and metabolic syndrome in Japanese: cross-sectional study

Journal:	*BMJ Open*
Manuscript ID:	bmjopen-2014-005241.R1
Article Type:	Research
Date Submitted by the Author:	31-May-2014
Complete List of Authors:	Nagahama, Satsue; All Japan Labor Welfare Foundation, ; School of Medicine, Toho University, Department of Environmental and Occupational Health<br>Kurotani, Kayo; National Center for Global Health and Medicine, Department of Epidemiology and Prevention, Center for Clinical Sciences<br>Pham, Ngoc Minh; Thai Nguyen University of Medicine and Pharmacy, Department of Epidemiology, Faculty of Public Health<br>Nanri, Akiko; National Center for Global Health and Medicine, Department of Epidemiology and Prevention, Center for Clinical Sciences<br>Kuwahara, Keisuke; National Center for Global Health and Medicine, Department of Epidemiology and Prevention, Center for Clinical Sciences<br>Dan, Masashi; All Japan Labor Welfare Foundation,<br>Nishiwaki, Yuji; School of Medicine, Toho University, Department of Environmental and Occupational Health<br>Mizoue, Tetsuya; National Center for Global Health and Medicine, Department of Epidemiology and Prevention, Center for Clinical Sciences
**Primary Subject Heading**:	Epidemiology
Secondary Subject Heading:	Epidemiology
Keywords:	EPIDEMIOLOGY, PREVENTIVE MEDICINE, PUBLIC HEALTH

SCHOLARONE™
Manuscripts

Self-reported eating rate and metabolic syndrome in Japanese: cross-sectional study

Satsue Nagahama [1 2 3*], Kayo Kurotani [1], Ngoc Minh Pham [4], Akiko Nanri [1], Keisuke Kuwahara [1], Masashi Dan [2], Yuji Nishiwaki [3], Tetsuya Mizoue [1]

[1] Department of Epidemiology and Prevention, Center for Clinical Sciences, National Center for Global Health and Medicine, 1-21-1, Toyama, Shinjuku-ku, Tokyo, Japan, Zip162-8655; Tel +81-3-3202-7181, Fax 81-3-3202-7364,

E-mail: satsue_0323@hotmail.com

[2] All Japan Labor Welfare Foundation, Tokyo, Japan;

[3] Department of Environmental and Occupational Health, School of Medicine, Toho University, Tokyo, Japan;

[4] Department of Epidemiology, Faculty of Public Health, Thai Nguyen University of Medicine and Pharmacy, Thai Nguyen Province, Vietnam;

Keywords: eating rate, metabolic syndrome, health checkup, Japan, Joint Interim Statement

For peer review only - http://bmjopen.bmj.com/site/about/guidelines.xhtml

Word count: 2373

For peer review only

For peer review only - http://bmjopen.bmj.com/site/about/guidelines.xhtml

ABSTRACT

Objectives: To examine the association between self-reported eating rate and metabolic syndrome.

Design: Cross-sectional study.

Setting: Annual health checkup at a health check service center in Japan.

Participants: A total of 56,865 participants (41,820 males and 15,045 females) who attended health checkup in 2011 and reported not to have a history of coronary heart disease or stroke.

Main outcome measure: Metabolic syndrome was defined by the joint of interim statement of the International Diabetes Federation and the American Heart Association/National Heart, Lung, and Blood Institute.

Results: In multiple logistic regression models, eating rate was significantly and positively associated with metabolic syndrome. The multivariable-adjusted odds ratios (95% confidence interval) for slow, normal and fast were 0.70 (0.62 to 0.79), 1.00 (reference) and 1.61 (1.53 to 1.70), respectively, in men (P for trend <0.001), and 0.74 (0.60 to 0.91), 1.00 (reference) and 1.27 (1.13 to 1.43), respectively, in women (P for trend <0.001). The association of eating rate and metabolic syndrome was attenuated after further adjustment for body mass index in both sexes, but remained statistically

For peer review only

significant in men. Of metabolic syndrome components, abdominal obesity showed the strongest association with eating rate. The associations of slow eating with high blood pressure and hyperglycemia and that of fast eating with lipid abnormality were still significant in men, even after adjustment for body mass index.

Conclusions: Results suggest that eating rate is associated with the presence of metabolic syndrome and that this association is largely accounted for by the difference of body mass according to eating rate.

For peer review only

For peer review only - http://bmjopen.bmj.com/site/about/guidelines.xhtml

Strength and Limitation

This study included a large number of participants, used waist circumference in defining metabolic syndrome, and analyzed data for men and women separately.

Eating rate was assessed by a self-reported questionnaire. Information on dietary intake was not obtained.

For peer review only

Introduction

Metabolic syndrome (MetS) is a cluster of physiological risk factors associated with cardiovascular disease and several types of cancer.[1] Determination of etiologic factors for MetS is required for the establishment of public health strategies to reduce its prevalence and prevent resulting complications. Growing evidence from both observational and interventional studies suggests a role of dietary habits in the development of MetS,[2-4] which originates from obesity. Obesity has been extensively investigated in relation to dietary habits including eating rate since 1962, when Ferster published a theoretical and practical weight control program focusing on eating behaviors including eating rate.[5] Observational studies showed that obese people ate at a faster rate than non-obese people,[6] and reducing eating rate may be a simple and effective therapy for obesity.[7]

During the past decade, several cross-sectional studies have found a positive association between eating rate and overweight[8-11] or insulin resistance.[11-15] Similarly, a few longitudinal studies showed that eating fast was associated with an increased risk of weight gain[16, 17] and type 2 diabetes.[18] In addition, some cross-sectional studies have reported that fast eating was positively associated with hypertriglyceridemia and low high-density lipoprotein cholesterol (HDL-C).[11, 14, 19] Therefore it is conceivable that

For peer review only - http://bmjopen.bmj.com/site/about/guidelines.xhtml

eating rate may be associated with MetS. To our knowledge, however, only one Korean study cross-sectionally examined eating rate in relation to MetS.[20] In that study, MetS was defined by using body mass index (BMI), rather than waist circumference, and investigated the association in men only. Waist circumference is a component of most MetS definitions as a surrogate of central obesity, which can better predict cardiovascular risk.[21] It is therefore necessary to examine the relationship between eating rate and MetS using waist circumference in both sexes. Here, we investigated cross-sectionally the association between self-reported eating rate and the presence of MetS according to the joint of interim statement of the International Diabetes Federation and the American Heart Association/National Heart, Lung, and Blood Institute (JIS)[22] using a large dataset of health checkup in Japanese men and women.

METHODS AND PROCEDURES

Study population

In Japan, health checkup under occupational health and safety law is mandatory for all employed workers[23] and has been modified in 2008 when the recommendation for new national health checkup system focusing on MetS has been launched.[24] Study participants were attendants of 2011 (calendar year) annual health checkup at All Japan

For peer review only - http://bmjopen.bmj.com/site/about/guidelines.xhtml

Labor Welfare Foundation (Tokyo), a health service center performing health checkup. Participants were mainly Japanese employees but also included a small number of their dependents and foreign workers, aged 17-99 in men and 17-85 in women. Of 297,148 participants, we excluded 3,660 with a history of myocardial infarction, coronary heart disease or stroke, 24,452 with missing information on eating rate, 192,581 who took meal within 8 hours or provided no information on meal time, 204,423 with missing data for any of the components of MetS, 15,886 with missing information on covariates (BMI, smoking status, alcohol consumption and physical activity). Some participants met more than one of these exclusion criteria, leaving 56,865 participants (41,820 males and 15,045 females) for analysis. We did not obtain written informed consent from each participant; instead, we disclosed the execution of the study by showing posters, giving participants an opportunity to refuse the use of their data for the study. This procedure conforms to the Japanese Ethical Guidelines for Epidemiological Research. The research protocol was approved by the Ethics Committee of the National Center for Global Health and Medicine and the Ethics Committee of Toho University.

Data collection and measurements

A self-administered questionnaire, which was recommended for specific health examination by the Japanese government (Ministry of Health, Labour and Welfare),[25]

For peer review only - http://bmjopen.bmj.com/site/about/guidelines.xhtml

was used to assess eating rate, medical history and health-related lifestyles including smoking, alcohol consumption and regular physical activity. Eating rate was assessed by asking "How fast is your speed of eating?", with three response options (slow, normal and fast). A trained staff measured height to the nearest 0.1 cm, weight to the nearest 0.1 kg and waist circumference to the nearest 0.1 cm at the umbilical level in a standing position. BMI was calculated as the weight in kilograms divided by the squared height in meters. Blood pressure in the sitting position was measured using an automated machine (HEM-907, Omron, Kyoto, Japan). Participants with high blood pressure (≥130 mm Hg systolic or ≥85 mm Hg diastolic) received another measurement and data showing lower systolic blood pressure was used. Venous blood sample was collected, stored in a cooler at 4 degrees for transportation to an external laboratory (SRL, Tokyo, Japan) and measured within 24 hours of blood drawing. Triglyceride level was measured by enzymatic colorimertic test (Bio Majesty JCA-BM8060, JEOL, Tokyo, Japan). HDL-C level was determined by a direct method (Bio Majesty JCA-BM8060, JEOL, Tokyo, Japan). Plasma glucose levels were determined using by the hexokinase method (an automatic clinical chemistry analyzer JCA-BM9000 series, JEOL, Tokyo, Japan).

Definitions for MetS

For peer review only - http://bmjopen.bmj.com/site/about/guidelines.xhtml

According to the JIS criteria, MetS was defined as three or more of the following risk factors: 1) waist circumference for Asian population ≥90 cm in men and ≥80 cm in women, 2) triglyceride level ≥150 mg/dL (1.7 mmol/L), 3) HDL-C level <40 mg/dL (1.04 mmol/L) in men and <50 mg/dL (1.3 mmol/L) in women, 4) blood pressure ≥130 mm Hg systolic or ≥85 mm Hg diastolic, 5) fasting glucose level ≥100 mg/dL (5.6 mmol/L).[22] Participants under medication for diabetes, hypertension and dyslipidemia were considered as having respective factor, irrespective of measured data.

Statistical analysis

Participants were divided into three groups according to eating rate (slow, normal and fast). The characteristics of participants across eating rate categories were expressed as means (standard deviation) for continuous variables and percentages for categorical variables, respectively. Fasting plasma glucose and triglyceride were expressed as medians (interquartile range) due to their skewed distribution. Trend association was assessed by assigning ordinal numbers (0 to 2) to the categories of eating rate (slow, normal and fast, respectively) and was tested using linear regression, an extension of the Wilcoxon Rank-Sum test and logistic regression, as appropriate. Multiple logistic regression analysis was used to estimate the odds ratios (OR) with 95% confidence intervals (CI) for the presence of MetS across eating rate categories,

For peer review only

For peer review only - http://bmjopen.bmj.com/site/about/guidelines.xhtml

with normal eating rate as the reference. We adjusted for age (continuous, year) in the basic model. In the second model, we further adjusted for smoking status (non-smoker, daily smoker consuming <20 cigarettes per day or ≥20 cigarettes per day), physical activity (walking time <60 min per day or ≥60 min per day), and alcohol consumption (non-drinker, <1 *go*, 1 to <2 *go* or ≥2 *go* per day; one *go* of sake, Japanese traditional beverage, is about 180 ml of 10 to 14% of ethanol and contains ~23g of ethanol). In the third model, we added BMI (continuous, kg/m^2) to the second model. We performed likelihood ratio test for testing the interaction between eating rate and sex. All analyses were done for men and women separately because the interaction was significant (P for interaction <0.001). We repeated the above analyses for each component of MetS. Two-tailed P value <0.05 was considered statistically significant. All statistical analyses were performed with STATA, version 12.1 (StataCorp, College Station, TX, USA).

RESULTS

The prevalence of MetS was 18.5% in men and 12.9% in women. **Table 1** shows characteristics of the study participants across categories of eating rate. Men who ate fast tended to be young, whereas women who ate slowly tended to be young. Those who ate fast had significantly higher BMI, waist circumference, triglyceride level and

For peer review only - http://bmjopen.bmj.com/site/about/guidelines.xhtml

systolic and diastolic blood pressures and lower HDL-C level in both men and women.

The ORs of the presence of MetS across eating rate are shown in **Table 2**. Faster eating was associated with higher presence of MetS in age- and multivariable-adjusted models. The trend was more apparent in men than in women. The multivariable-adjusted ORs (95% CI) of MetS for eating slow, normal and fast rate were 0.70 (0.62 to 0.79), 1.00 (reference) and 1.61 (1.53 to 1.70), respectively, in men (P for trend <0.001), and 0.74 (0.60 to 0.91), 1.00 (reference) and 1.27 (1.13 to 1.43), respectively, in women (P for trend <0.001). Further adjustment for BMI markedly attenuated these associations; however, the association with fast eating and MetS remained statistically significant in men.

Table 3 shows the ORs of the presence of individual MetS components across three categories of eating rate. The ORs of central obesity increased from slow to fast eating in both men and women (OR 0.63, 1.00 (reference) and 1.97, respectively, in men (P for trend <0.001), and 0.73, 1.00(reference) and 1.44, respectively, in women (P for trend <0.001)). High blood pressure and triglyceride were positively associated with eating rate in both sexes. High fasting plasma glucose and low HDL-C were associated with slow eating in both sexes, but they were associated with fast eating in men only. Additional adjustment for BMI largely attenuated these associations, but the

For peer review only

For peer review only - http://bmjopen.bmj.com/site/about/guidelines.xhtml

associations of slow eating with high blood pressure (men and women) and hyperglycemia (men) and those of fast eating with abnormal lipid profile (men) remained statistically significant.

DISCUSSION

In this large population of Japanese men and women, we found that eating rate was positively associated with the presence of MetS, especially in men. Of components of MetS, the association with abdominal obesity was strongest. The relationship with blood pressure in both sexes and fasting plasma glucose and lipids in men remained statistically significant even after additional adjustment for BMI. To our best knowledge, the present study is the first to report a positive association between eating rate and MetS defined by using waist circumference.

The present finding for MetS is consistent with that of a study among Korean men reporting that eating rate was positively associated with MetS, which was defined by using BMI instead of waist circumference.[20] As regards MetS components, our study is compatible with some cross-sectional studies showing that eating rate was associated with higher BMI[8-10] and two longitudinal studies showing that eating rate was associated with weight gain.[16, 17] In a Korean study that elucidated the association

For peer review only - http://bmjopen.bmj.com/site/about/guidelines.xhtml

between eating rate and components of MetS for men and women separately, eating rate was associated with obesity, high blood pressure, hyperglycemia and abnormal lipid profile in men, whereas it was associated with only obesity in women.[11] Our results were largely consistent with those in the Korean study (except for blood pressure in women).

Notably, we found that the associations of MetS components with eating rate were largely attenuated after adjustment for BMI, a finding compatible with those of a cross-sectional study in Korea[11] and a prospective study in Japan.[18] This result indicates that obesity is a mediator whereby fast eating deteriorates MetS components. We also found, however, that some associations remained statistically significant even after adjusting for BMI (dyslipidemia with fast eating and hyperglycemia with slow eating in men, and high blood pressure with slow eating in both men and women). Similarly, the above-mentioned Korean study[11] reported that high rate of eating remained an important determinant for low HDL-C and high fasting plasma glucose after adjustment for BMI in men. Therefore, there may be pathways other than weight gain that might underlie the association between eating rate and MetS.

We found that the association between eating rate and MetS was stronger in men than in women, consistent with a previous study in Korea.[11] Such sex difference

For peer review only - http://bmjopen.bmj.com/site/about/guidelines.xhtml

may reflect the difference in actual eating speed between men and women. One study elucidated that women took more bites, smaller bite size and slower bites than men in eating the same amount of doughnut, irrespectively of body size.[26] Another study showed that objectively measured eating speed in men with self-reported slow eating was faster than that in women with self-reported fast eating.[27] Taken together, eating rate may have a greater impact on metabolism in men than that in women.

Although mechanisms whereby eating rate influences metabolism is not fully elucidated, overeating may link fast eating to MetS. Fast eating gives few satiety signal from oral cavity to the brain,[28, 29] induces less satiation and satiety due to a lack of stomach expansion[30] and alters the circulating levels of certain gut hormones.[31, 32] In these pathways, fast eating leads to excess energy intake,[33, 34] resulting in overweight and MetS. Because fast eating has been associated with obesity even after adjusting for total energy intake,[8-11, 14] there may be other pathways. One study showed that interleukin-1β and interleukin-6 were higher among those who ate fast than among those who ate slowly, even after accounting for energy intake and BMI.[35] These cytokines could induce insulin resistance,[36, 37] contributing to high blood pressure via an increased renal sodium and water retention, plasma noradrenalin and sympathetic nervous system activity.[38-40]

For peer review only

For peer review only - http://bmjopen.bmj.com/site/about/guidelines.xhtml

The strengths of our study deserve mention. The present study has large sample size (56,865 participants). In addition, body weight, body height and waist circumference were measured by trained technicians, which increased the validity of our study. Nonetheless, several limitations in the present study merit consideration. First, eating rate was self-reported. However, self-reported eating rate has been shown to be well correlated with friend-reported one[9] or objectively measured one.[27] Second, total energy intake was not available in our study. However, because energy intake is influenced by eating rate and thus may act as a mediator rather than confounder, the adjustment of energy intake could underestimate the association between eating rate and MetS. Moreover, eating rate has been associated with body weight independent of energy intake.[7-10, 13] Third, the study participants were mainly workers in various industries including manufacturing (43.6%), service (27.8%) and transport and telecommunications (9.9%), and these figures are similar to those of national survey.[41] However, information on profession of participants was not available, and thus caution is required when generalize the present finding. Fourth, cross-sectional design precludes any causal inferences about the role of eating rate. Finally, we cannot exclude a possibility of the effects of residual confounding and confounding by unmeasured variables.

For peer review only - http://bmjopen.bmj.com/site/about/guidelines.xhtml

In conclusion, we found a positive trend association between self-reported eating rate and the presence of MetS in men and women. The association between eating rate and MetS was largely accounted for by the difference of body mass across eating rate. Further research should address whether reducing eating rate prevents obesity and MetS.

For peer review only

For peer review only - http://bmjopen.bmj.com/site/about/guidelines.xhtml

Table1. Characteristics of the study individuals according to eating rates

	Men (n = 41,820)				Women (n = 15,045)			
	Slow	Normal	Fast	P for trend [a]	Slow	Normal	Fast	P for trend [a]
n (%)	2,821 (6.8)	24,893 (59.5)	14,106 (33.7)		1,398 (9.3)	9,893 (65.8)	3,754 (24.9)	
Age (years) [b]	46.9 ± 12.3	46.9 ± 10.9	45.0 ± 10.4	<0.001	43.5 ± 12.5	47.2 ± 11.6	46.7 ± 11.2	<0.001
Walking time, ≥60 min/day (%)	21.8	19.0	20.6	0.004	15.5	15.0	16.1	0.798
Smoking status (%)								
Non-smoker	61.9	55.0	56.6	<0.001	82.9	83.1	80.7	0.572

For peer review only

For peer review only - http://bmjopen.bmj.com/site/about/guidelines.xhtml

Daily consuming <20 cigarettes /day	28.6	34.6	31.3		16.0	15.7	17.6	
Daily consuming ≥20 cigarettes /day	9.5	10.4	12.1		1.1	1.2	1.7	
Alcohol (%)								
Non-drinker	30.2	26.7	26.7	<0.001	53.4	52.2	49.8	<0.001
Drinker <1 *go* /day [d]	33.9	35.7	34.5		34.2	35.9	35.5	
Drinker 1 to <2 *go* /day [d]	24.6	26.3	26.8		9.5	9.5	11.5	
Drinker ≥2 *go* /day [d]	11.3	11.3	12.0		2.9	2.4	3.2	
BMI (kg/m^2)[b]	22.4 ± 3.3	23.4 ± 3.3	24.6 ± 3.7	<0.00	21.0 ± 3.5	21.8 ± 3.5	22.5 ± 3.8	<0.00

For peer review only - http://bmjopen.bmj.com/site/about/guidelines.xhtml

For peer review only

				1				1
Waist circumference (cm)[b]	80.3 ± 9.2	82.9 ± 9.0	86.0 ± 9.8	<0.001	75.5 ± 9.5	77.7 ± 9.4	79.6 ± 9.8	<0.001
Systolic blood pressure (mm Hg)[b]	123.5 ± 15.5	126.1 ± 15.5	126.7 ± 15.1	<0.001	113.1 ± 16.3	117.3 ± 17.2	117.0 ± 17.2	<0.001
Diastolic blood pressure (mm Hg)[b]	75.2± 11.4	77.3 ± 11.9	78.0 ± 12.0	<0.001	69.1 ± 10.9	71.4 ± 11.5	71.5 ± 11.9	<0.001
Fasting plasma glucose (mg/dL)[c]	91 (85 to 98)	92 (86 to 99)	92 (86 to 99)	0.001	87 (82 to 93)	88 (83 to 94)	88 (83 to 94)	0.001
Triglyceride (mg/dL)[c]	92 (65 to 138)	99 (69 to 148)	107 (73 to 161)	<0.001	63 (48 to 87)	69 (51 to 96)	71 (52 to 101)	<0.001

For peer review only

HDL-C (mg/dL)[b]	61.3 ± 15.3	59.4 ± 15.0	57.2 ± 14.3	<0.001	71.4 ± 15.3	70.5 ± 15.8	69.3 ± 15.5	<0.001

Cross to sectional survey of 56,865 examinees in All Japan Labor Welfare Foundation, Japan, 2011.

BMI=body mass index; HDL-C=high to density lipoprotein cholesterol.

[a] Linear regression, an extension of the Wilcoxon Rank-Sum test and logistic regression, assigning ordinal number (0 to 2) to eating rate, as appropriate.

[b] Mean ± SD.

[c] medians (interquartile range).

[d] One *go* contains ~25g of ethanol.

For peer review only - http://bmjopen.bmj.com/site/about/guidelines.xhtml

Table2. Odds ratios and 95% confidence intervals for metabolic syndrome according to eating rate (n = 56,865)

	Men (n = 41,820)				Women (n = 15,045)			
Eating rate	Slow	Normal [a]	Fast	P for trend [b]	Slow	Normal [a]	Fast	P for trend [b]
n (%)	2,821 (6.8)	24,893 (59.5)	14,106 (33.7)		1,398 (9.3)	9,893 (65.8)	3,754 (24.9)	
MetS, n	361	4,180	3,193		116	1,261	547	
Model 1 [c]	0.70 (0.62 to 0.79)	1.00	1.62 (1.53 to 1.71)	<0.001	0.75 (0.61 to 0.92)	1.00	1.27 (1.13 to 1.42)	<0.001
Model 2 [d]	0.70 (0.62 to 0.79)	1.00	1.61 (1.53 to 1.70)	<0.001	0.74 (0.60 to 0.91)	1.00	1.27 (1.13 to 1.43)	<0.001

For peer review only - http://bmjopen.bmj.com/site/about/guidelines.xhtml

Model 3[e]	0.91 (0.80 to 1.04)	1.00	1.10 (1.03 to 1.17)	<0.001	0.88 (0.70 to 1.11)	1.00	0.98 (0.86 to 1.12)	0.714

MetS as defined using the criteria of the Joint Interim Statement : the presence of three or more of the following risk factors: 1) waist circumference ≥90 cm in men and ≥80 cm in women, 2) triglyceride level ≥150 mg/dL (1.7 mmol/L), 3) HDL-C level <40 mg/dL (1.04 mmol/L) in men and <50 mg/dL (1.3 mmol/L) in women, 4) blood pressure ≥130 mm Hg systolic or ≥85 mm Hg diastolic, 5) fasting glucose level ≥100 mg/dL (5.6 mmol/L).

[a] Reference.

[b] Multiple logistic regression, assigning ordinal number (0 to 2) to eating rate.

[c] Adjusted for age.

[d] Adjusted for age, smoking status, alcohol, and regular physical activity.

[e] Adjusted for age, smoking status, alcohol, regular physical activity, and body mass index.

For peer review only - http://bmjopen.bmj.com/site/about/guidelines.xhtml

Table3. Odds ratios and 95% confidence intervals for components of metabolic syndrome according to eating rate (n = 56,865)

Eating rate	Men (n = 41,820)				Women (n = 15,045)			
	Slow	Normal [a]	Fast	P for trend [b]	Slow	Normal [a]	Fast	P for trend [b]
n (%)	2,821 (6.8)	24,893 (59.5)	14,106 (33.7)		1,398 (9.3)	9,893 (65.8)	3,754 (24.9)	
Central obesity [c]								
Model 1 [d]	0.63 (0.56 to 0.71)	1.00	1.98 (1.89 to 2.08)	<0.001	0.73 (0.64 to 0.83)	1.00	1.44 (1.34 to 1.56)	<0.001
Model 2 [e]	0.63 (0.56 to 0.70)	1.00	1.97 (1.88 to 2.07)	<0.001	0.73 (0.64 to 0.83)	1.00	1.44 (1.33 to 1.56)	<0.001

For peer review only - http://bmjopen.bmj.com/site/about/guidelines.xhtml

For peer review only

High blood pressure [f]								
Model 1 [d]	0.75 (0.69 to 0.82)	1.00	1.22 (1.17 to 1.27)	<0.001	0.76 (0.66 to 0.88)	1.00	1.10 (1.01 to 1.21)	<0.001
Model 2 [e]	0.74 (0.68 to 0.81)	1.00	1.20 (1.15 to 1.26)	<0.001	0.76 (0.65 to 0.88)	1.00	1.10 (1.00 to 1.20)	<0.001
Model 3 [g]	0.88 (0.81 to 0.96)	1.00	0.97 (0.93 to 1.02)	0.645	0.85 (0.72 to 0.99)	1.00	0.93 (0.84 to 1.02)	0.923
High fasting plasma glucose [h]								
Model 1 [d]	0.78 (0.71 to 0.87)	1.00	1.17 (1.12 to 1.23)	<0.001	1.03 (0.85 to 1.25)	1.00	1.17 (1.04 to 1.31)	0.035
Model 2 [e]	0.78 (0.71 to	1.00	1.16 (1.11 to	<0.00	1.03 (0.85 to	1.00	1.16 (1.03 to	0.042

For peer review only

For peer review only - http://bmjopen.bmj.com/site/about/guidelines.xhtml

	0.87)		1.22)	1	1.25)		1.31)	
Model 3 [g]	0.88 (0.80 to 0.98)	1.00	0.99 (0.94 to 1.05)	0.238	1.14 (0.94 to 1.40)	1.00	1.02 (0.90 to 1.15)	0.536
High triglyceride [i]								
Model 1 [d]	0.88 (0.80 to 0.96)	1.00	1.32 (1.26 to 1.38)	<0.001	0.83 (0.67 to 1.01)	1.00	1.14 (1.01 to 1.28)	0.002
Model 2 [e]	0.90 (0.82 to 0.98)	1.00	1.33 (1.27 to 1.39)	<0.001	0.81 (0.66 to 1.00)	1.00	1.13 (1.01 to 1.27)	0.002
Model 3 [g]	1.08 (0.98 to 1.19)	1.00	1.07 (1.02 to 1.12)	0.121	0.90 (0.73 to 1.11)	1.00	0.98 (0.87 to 1.11)	0.753
Low HDL-C [j]								

For peer review only

Model 1 [c]	0.83 (0.73 to 0.96)	1.00	1.34 (1.26 to 1.43)	<0.001	0.90 (0.74 to 1.09)	1.00	1.11 (0.99 to 1.25)	0.018
Model 2 [d]	0.83 (0.73 to 0.96)	1.00	1.36 (1.28 to 1.45)	<0.001	0.89 (0.74 to 1.08)	1.00	1.12 (1.00 to 1.26)	0.011
Model 3 [g]	0.97 (0.84 to 1.12)	1.00	1.10 (1.03 to 1.18)	0.004	1.00 (0.82 to 1.22)	1.00	0.96 (0.85 to 1.08)	0.500

For peer review only

BMI=body mass index; HDL-C=high to density lipoprotein cholesterol.

[a] Reference.

[b] Multiple logistic regression, assigning ordinal number (0 to 2) to eating rate.

[c] Waist circumference ≥90 cm in men, ≥80 cm in women.

[d] Adjusted for age.

[e] Adjusted for age, smoking status, alcohol, and regular physical activity.

[f] Blood pressure ≥130 mm Hg for systolic or ≥85 mm Hg for diastolic.

[g] Adjusted for age, smoking status, alcohol, regular physical activity and body mass index.

[h] Fasting plasma glucose ≥100 mg/dL or under medication.

[i] Triglyceride ≥150 mg/dL or under medication.

[j] HDL-C <40 mg/dL in men, <50 mg/dL in women or under medication.

For peer review only

For peer review only - http://bmjopen.bmj.com/site/about/guidelines.xhtml

Acknowledgments

The authors would like to thank Dr. Nobuo Yanagisawa and Dr. Takeshi Kawaguchi for their coordinating the study.

Contributors: SN and KKurotani designed study and drafted the manuscript. SN, NMP, AN, KKuwahara performed the data analysis. MD collected and interpreted the data. All authors have participated in the interpretation of the findings, revised it critically for important intellectual content and approved final version to be published. TM and YN provided administrative, technical and material support.SN and TM are guarantors.

Funding: This study was supported by the Industrial Health Foundation.

Competing interests: All authors have completed the ICMJE uniform disclosure form at www.icmje.org/coi_disclosure.pdf and declare: all authors declare no interests.

Ethical approval: The research protocol was approved by the Ethics Committee of the National Center for Global Health and Medicine and the Ethics Committee of Toho University.

Data sharing: No additional data available.

Transparency: SN affirms that the manuscript is an honest, accurate, and transparent account of the study being reported; that no important aspects of the study have been omitted; and that any discrepancies from the study as planned (and, if relevant,

For peer review only

registered) have been explained.

For peer review only

For peer review only - http://bmjopen.bmj.com/site/about/guidelines.xhtml

References

1. Eckel RH, Alberti KG, Grundy SM, et al. The metabolic syndrome. *Lancet* 2010;**375**:181-83.

2. Gomez-Martinez S, Martinez-Gomez D, Perez de Heredia F, et al. Eating habits and total and abdominal fat in Spanish adolescents: influence of physical activity. The AVENA study. *J Adolesc Health* 2012;**50**:403-09.

3. Ilanne-Parikka P, Eriksson JG, Lindstrom J, et al. Effect of lifestyle intervention on the occurrence of metabolic syndrome and its components in the Finnish Diabetes Prevention Study. *Diabetes care* 2008;**31**:805-07.

4. Nanri A, Tomita K, Matsushita Y, et al. Effect of six months lifestyle intervention in Japanese men with metabolic syndrome: randomized controlled trial. *J Occup Health* 2012;**54**:215-22.

5. Ferster CB, Nurnberger JI, Levitt EB. The control of eating. 1962. *Obes Res* 1996;**4**:401-10.

6. Hill SW, McCutcheon NB. Eating responses of obese and nonobese humans during dinner meals. *Psychosom Med* 1975;**37**:395-401.

7. Bellack AS. Behavior therapy for weight reduction. *Addict Behav* 1975;**1**:73-82.

8. Otsuka R, Tamakoshi K, Yatsuya H, et al. Eating fast leads to obesity: findings based

For peer review only

For peer review only - http://bmjopen.bmj.com/site/about/guidelines.xhtml

on self-administered questionnaires among middle-aged Japanese men and women. *J Epidemiol* 2006;**16**:117-24.

9. Sasaki S, Katagiri A, Tsuji T, et al. Self-reported rate of eating correlates with body mass index in 18-y-old Japanese women. *Int J Obes Relat Metab Disord* 2003;**27**:1405-10.

10. Maruyama K, Sato S, Ohira T, et al. The joint impact on being overweight of self reported behaviours of eating quickly and eating until full: cross sectional survey. *BMJ* 2008;**337**:a2002.

11. Lee KS, Kim DH, Jang JS, et al. Eating rate is associated with cardiometabolic risk factors in Korean adults. *Nut Metab Cardiovasc Dis* 2013;**23**:635-41.

12. Otsuka R, Tamakoshi K, Yatsuya H, et al. Eating fast leads to insulin resistance: findings in middle-aged Japanese men and women. *Prev Med* 2008;**46**:154-59.

13. Radzeviciene L, Ostrauskas R. Fast eating and the risk of type 2 diabetes mellitus: a case-control study. *Clin Nutr* 2013;**32**:232-35.

14. Ohkuma T, Fujii H, Iwase M, et al. Impact of eating rate on obesity and cardiovascular risk factors according to glucose tolerance status: the Fukuoka Diabetes Registry and the Hisayama Study. *Diabetologia* 2013;**56**:70-77.

15. Shigeta H, Shigeta M, Nakazawa A, et al. Lifestyle, obesity, and insulin resistance.

For peer review only

For peer review only - http://bmjopen.bmj.com/site/about/guidelines.xhtml

Diabetes care 2001;**24**:608.

16. Gerace TA, George VA. Predictors of weight increases over 7 years in fire fighters and paramedics. *Prev Med* 1996;**25**:593-600.

17. Tanihara S, Imatoh T, Miyazaki M, et al. Retrospective longitudinal study on the relationship between 8-year weight change and current eating speed. *Appetite* 2011;**57**:179-83.

18. Sakurai M, Nakamura K, Miura K, et al. Self-reported speed of eating and 7-year risk of type 2 diabetes mellitus in middle-aged Japanese men. *Metabolism* 2012;**61**:1566-71.

19. Kral JG, Buckley MC, Kissileff HR, et al. Metabolic correlates of eating behavior in severe obesity. *Int J Obes Relat Metab Disord* 2001;**25**:258-64.

20. Shin A, Lim SY, Sung J, et al. Dietary intake, eating habits, and metabolic syndrome in Korean men. *J Am Diet Assoc* 2009;**109**:633-40.

21. Shen W, Punyanitya M, Chen J, et al. Waist circumference correlates with metabolic syndrome indicators better than percentage fat. *Obesity (Silver Spring)* 2006;**14**:727-36.

22. Alberti KG, Eckel RH, Grundy SM, et al. Harmonizing the metabolic syndrome: a joint interim statement of the International Diabetes Federation Task Force on Epidemiology and Prevention; National Heart, Lung, and Blood Institute; American

For peer review only

For peer review only - http://bmjopen.bmj.com/site/about/guidelines.xhtml

Heart Association; World Heart Federation; International Atherosclerosis Society; and International Association for the Study of Obesity. *Circulation* 2009;**120**:1640-5.

23. Ministry of Justice, Japan. Industrial Safty and Health Act, Article 66 (Medical Examination). Available at: http://www.japaneselawtranslation.go.jp/law/detail/?id=1926&vm=&re=. Accessed 26 May 2014.

24. Ministry of Health, Labour and Welfare. Specific Health Checkups and Specific Health Guidance. Secondary Specific Health Checkups and Specific Health Guidance. Available at: http://www.mhlw.go.jp/english/wp/wp-hw3/dl/2-007.pdf. Accessed 26 May 2014.

25. Ministry of Health, Labour and Welfare. Questionnaire of Specific Health Checkups. Secondary Questionnaire of Specific Health Checkups. Available at: http://www.mhlw.go.jp/seisakunitsuite/bunya/kenkou_iryou/kenkou/seikatsu/dl/hoken-program2_02.pdf. Accessed 26 May 2014. (in Japanese)

26. Hill SW, McCutcheon NB. Contributions of obesity, gender, hunger, food preference, and body size to bite size, bite speed, and rate of eating. *Appetite* 1984;**5**:73-83.

27. Ekuni D, Furuta M, Takeuchi N, et al. Self-reports of eating quickly are related to a decreased number of chews until first swallow, total number of chews, and total

review only

For peer review only - http://bmjopen.bmj.com/site/about/guidelines.xhtml

duration of chewing in young people. *Arch Oral Biol* 2012;**57**:981-86.

28. Rolls ET. Sensory processing in the brain related to the control of food intake. *Proc Nutr Soc* 2007;**66**:96-112.

29. Morton GJ, Cummings DE, Baskin DG, et al. Central nervous system control of food intake and body weight. *Nature* 2006;**443**:289-95.

30. de Graaf C, Blom WA, Smeets PA, et al. Biomarkers of satiation and satiety. *Am J Clin Nutr* 2004;**79**:946-61.

31. Kokkinos A, le Roux CW, Alexiadou K, et al. Eating slowly increases the postprandial response of the anorexigenic gut hormones, peptide YY and glucagon-like peptide-1. *J Clin Endocrinol Metab* 2010;**95**:333-37.

32. Sobki SH, Zaid AA, Khan HA, et al. Significant impact of pace of eating on serum ghrelin and glucose levels. *Clin Biochem* 2010;**43**:522-24.

33. Viskaal-van Dongen M, Kok FJ, de Graaf C. Eating rate of commonly consumed foods promotes food and energy intake. *Appetite* 2011;**56**:25-31.

34. Scisco JL, Muth ER, Dong Y, et al. Slowing bite-rate reduces energy intake: an application of the bite counter device. *J Am Diet Assoc* 2011;**111**:1231-35.

35. Mochizuki K, Misaki Y, Miyauchi R, et al. A higher rate of eating is associated with higher circulating interluekin-1beta concentrations in Japanese men not being treated for

For peer review only

For peer review only - http://bmjopen.bmj.com/site/about/guidelines.xhtml

metabolic diseases. *Nutrition* 2012;**28**:978-83.

36. Besedovsky H, del Rey A, Sorkin E, et al. Immunoregulatory feedback between interleukin-1 and glucocorticoid hormones. *Science* 1986;**233**:652-54

37. Matsuki T, Horai R, Sudo K, et al. IL-1 plays an important role in lipid metabolism by regulating insulin levels under physiological conditions. *J Exp Med* 2003;**198**:877-88.

38. Yatabe MS, Yatabe J, Yoneda M, et al. Salt sensitivity is associated with insulin resistance, sympathetic overactivity, and decreased suppression of circulating renin activity in lean patients with essential hypertension. *Am J Clin Nutr* 2010;**92**:77-82.

39. Facchini FS, DoNascimento C, Reaven GM, et al. Blood pressure, sodium intake, insulin resistance, and urinary nitrate excretion. *Hypertension* 1999;**33**:1008-12.

40. Pollare T, Lithell H, Berne C. Insulin resistance is a characteristic feature of primary hypertension independent of obesity. *Metabolism*. 1990;**39**:167-74.

41. Ministry of Health, Labour and Welfare. Special Survey on Industrial Safety and Health. Available at: http://www.mhlw.go.jp/bunya/roudoukijun/anzeneisei11/h23.html. Accessed 26 May 2014. (in Japanese)

For peer review only - http://bmjopen.bmj.com/site/about/guidelines.xhtml

Self-reported eating rate and metabolic syndrome in Japanese: cross-sectional study

Satsue Nagahama [1 2 3*], Kayo Kurotani [1], Ngoc Minh Pham [4], Akiko Nanri [1], Keisuke Kuwahara [1], Masashi Dan [2], Yuji Nishiwaki [3], Tetsuya Mizoue [1]

[1] Department of Epidemiology and Prevention, Center for Clinical Sciences, National Center for Global Health and Medicine, 1-21-1, Toyama, Shinjuku-ku, Tokyo, Japan, Zip162-8655; Tel +81-3-3202-7181, Fax 81-3-3202-7364,

E-mail: satsue_0323@hotmail.com

[2] All Japan Labor Welfare Foundation, Tokyo, Japan;

[3] Department of Environmental and Occupational Health, School of Medicine, Toho University, Tokyo, Japan;

[4] Department of Epidemiology, Faculty of Public Health, Thai Nguyen University of Medicine and Pharmacy, Thai Nguyen Province, Vietnam;

Keywords: eating rate, metabolic syndrome, health checkup, Japan, Joint Interim Statement

For peer review only - http://bmjopen.bmj.com/site/about/guidelines.xhtml

19 Word count: 2373

For peer review only

For peer review only - http://bmjopen.bmj.com/site/about/guidelines.xhtml

ABSTRACT

Objectives: To examine the association between self-reported eating rate and metabolic syndrome.

Design: Cross-sectional study.

Setting: Annual health checkup at a health check service center in Japan.

Participants: A total of 56,865 participants (41,820 males and 15,045 females) who attended health checkup in 2011 and reported not to have a history of coronary heart disease or stroke.

Main outcome measure: Metabolic syndrome was defined by the joint of interim statement of the International Diabetes Federation and the American Heart Association/National Heart, Lung, and Blood Institute.

Results: In multiple logistic regression models, eating rate was significantly and positively associated with metabolic syndrome. The multivariable-adjusted odds ratios (95% confidence interval) for slow, normal and fast were 0.70 (0.62 to 0.79), 1.00 (reference) and 1.61 (1.53 to 1.70), respectively, in men (P for trend <0.001), and 0.74 (0.60 to 0.91), 1.00 (reference) and 1.27 (1.13 to 1.43), respectively, in women (P for trend <0.001). The association of eating rate and metabolic syndrome was attenuated after further adjustment for body mass index in both sexes, but remained statistically

For peer review only - http://bmjopen.bmj.com/site/about/guidelines.xhtml

significant in men. Of metabolic syndrome components, abdominal obesity showed the strongest association with eating rate. The associations of slow eating with high blood pressure and hyperglycemia and that of fast eating with lipid abnormality were still significant in men, even after adjustment for body mass index.

Conclusions: Results suggest that eating rate is associated with the presence of metabolic syndrome and that this association is largely accounted for by the difference of body mass according to eating rate.

For peer review only

Strength and Limitation

This study included a large number of participants, used waist circumference in defining metabolic syndrome, and analyzed data for men and women separately.

Eating rate was assessed by a self-reported questionnaire. Information on dietary intake was not obtained.

For peer review only

For peer review only - http://bmjopen.bmj.com/site/about/guidelines.xhtml

Introduction

Metabolic syndrome (MetS) is a cluster of physiological risk factors associated with cardiovascular disease and several types of cancer.[1] Determination of etiologic factors for MetS is required for the establishment of public health strategies to reduce its prevalence and prevent resulting complications. Growing evidence from both observational and interventional studies suggests a role of dietary habits in the development of MetS,[2-4] which originates from obesity. Obesity has been extensively investigated in relation to dietary habits including eating rate since 1962, when Ferster published a theoretical and practical weight control program focusing on eating behaviors including eating rate.[5] Observational studies showed that obese people ate at a faster rate than non-obese people,[6] and reducing eating rate may be a simple and effective therapy for obesity.[7]

During the past decade, several cross-sectional studies have found a positive association between eating rate and overweight[8-11] or insulin resistance.[11-15] Similarly, a few longitudinal studies showed that eating fast was associated with an increased risk of weight gain[16, 17] and type 2 diabetes.[18] In addition, some cross-sectional studies have reported that fast eating was positively associated with hypertriglyceridemia and low high-density lipoprotein cholesterol (HDL-C).[11, 14, 19] Therefore it is conceivable that

For peer review only - http://bmjopen.bmj.com/site/about/guidelines.xhtml

eating rate may be associated with MetS. To our knowledge, however, only one Korean study cross-sectionally examined eating rate in relation to MetS.[20] In that study, MetS was defined by using body mass index (BMI), rather than waist circumference, and investigated the association in men only. Waist circumference is a component of most MetS definitions as a surrogate of central obesity, which can better predict cardiovascular risk.[21] It is therefore necessary to examine the relationship between eating rate and MetS using waist circumference in both sexes. Here, we investigated cross-sectionally the association between self-reported eating rate and the presence of MetS according to the joint of interim statement of the International Diabetes Federation and the American Heart Association/National Heart, Lung, and Blood Institute (JIS)[22] using a large dataset of health checkup in Japanese men and women.

METHODS AND PROCEDURES

Study population

In Japan, health checkup under occupational health and safety law is mandatory for all employed workers[23] and has been modified in 2008 when the recommendation for new national health checkup system focusing on MetS has been launched.[24] Study participants were attendants of 2011 (calendar year) annual health checkup at All Japan

For peer review only - http://bmjopen.bmj.com/site/about/guidelines.xhtml

Labor Welfare Foundation (Tokyo), a health service center performing health checkup. Participants were mainly Japanese employees but also included a small number of their dependents and foreign workers, aged 17-99 in men and 17-85 in women. Of 297,148 participants, we excluded 3,660 with a history of myocardial infarction, coronary heart disease or stroke, 24,452 with missing information on eating rate, 192,581 who took meal within 8 hours or provided no information on meal time, 204,423 with missing data for any of the components of MetS, 15,886 with missing information on covariates (BMI, smoking status, alcohol consumption and physical activity). Some participants met more than one of these exclusion criteria, leaving 56,865 participants (41,820 males and 15,045 females) for analysis. We did not obtain written informed consent from each participant; instead, we disclosed the execution of the study by showing posters, giving participants an opportunity to refuse the use of their data for the study. This procedure conforms to the Japanese Ethical Guidelines for Epidemiological Research. The research protocol was approved by the Ethics Committee of the National Center for Global Health and Medicine and the Ethics Committee of Toho University.

Data collection and measurements

A self-administered questionnaire, which was recommended for specific health examination by the Japanese government (Ministry of Health, Labour and Welfare),[25]

For peer review only - http://bmjopen.bmj.com/site/about/guidelines.xhtml

was used to assess eating rate, medical history and health-related lifestyles including smoking, alcohol consumption and regular physical activity. Eating rate was assessed by asking "How fast is your speed of eating?", with three response options (slow, normal and fast). A trained staff measured height to the nearest 0.1 cm, weight to the nearest 0.1 kg and waist circumference to the nearest 0.1 cm at the umbilical level in a standing position. BMI was calculated as the weight in kilograms divided by the squared height in meters. Blood pressure in the sitting position was measured using an automated machine (HEM-907, Omron, Kyoto, Japan). Participants with high blood pressure (≥130 mm Hg systolic or ≥85 mm Hg diastolic) received another measurement and data showing lower systolic blood pressure was used. Venous blood sample was collected, stored in a cooler at 4 degrees for transportation to an external laboratory (SRL, Tokyo, Japan) and measured within 24 hours of blood drawing. Triglyceride level was measured by enzymatic colorimertic test (Bio Majesty JCA-BM8060, JEOL, Tokyo, Japan). HDL-C level was determined by a direct method (Bio Majesty JCA-BM8060, JEOL, Tokyo, Japan). Plasma glucose levels were determined using by the hexokinase method (an automatic clinical chemistry analyzer JCA-BM9000 series, JEOL, Tokyo, Japan).

Definitions for MetS

For peer review only

For peer review only - http://bmjopen.bmj.com/site/about/guidelines.xhtml

According to the JIS criteria, MetS was defined as three or more of the following risk factors: 1) waist circumference for Asian population ≥90 cm in men and ≥80 cm in women, 2) triglyceride level ≥150 mg/dL (1.7 mmol/L), 3) HDL-C level <40 mg/dL (1.04 mmol/L) in men and <50 mg/dL (1.3 mmol/L) in women, 4) blood pressure ≥130 mm Hg systolic or ≥85 mm Hg diastolic, 5) fasting glucose level ≥100 mg/dL (5.6 mmol/L).[22] Participants under medication for diabetes, hypertension and dyslipidemia were considered as having respective factor, irrespective of measured data.

Statistical analysis

Participants were divided into three groups according to eating rate (slow, normal and fast). The characteristics of participants across eating rate categories were expressed as means (standard deviation) for continuous variables and percentages for categorical variables, respectively. Fasting plasma glucose and triglyceride were expressed as medians (interquartile range) due to their skewed distribution. Trend association was assessed by assigning ordinal numbers (0 to 2) to the categories of eating rate (slow, normal and fast, respectively) and was tested using linear regression, an extension of the Wilcoxon Rank-Sum test and logistic regression, as appropriate. Multiple logistic regression analysis was used to estimate the odds ratios (OR) with 95% confidence intervals (CI) for the presence of MetS across eating rate categories,

For peer review only - http://bmjopen.bmj.com/site/about/guidelines.xhtml

with normal eating rate as the reference. We adjusted for age (continuous, year) in the basic model. In the second model, we further adjusted for smoking status (non-smoker, daily smoker consuming <20 cigarettes per day or ≥20 cigarettes per day), physical activity (walking time <60 min per day or ≥60 min per day), and alcohol consumption (non-drinker, <1 *go*, 1 to <2 *go* or ≥2 *go* per day; one *go* of sake, Japanese traditional beverage, is about 180 ml of 10 to 14% of ethanol and contains ~23g of ethanol). In the third model, we added BMI (continuous, kg/m^2) to the second model. We performed likelihood ratio test for testing the interaction between eating rate and sex. All analyses were done for men and women separately because the interaction was significant (P for interaction <0.001). We repeated the above analyses for each component of MetS. Two-tailed P value <0.05 was considered statistically significant. All statistical analyses were performed with STATA, version 12.1 (StataCorp, College Station, TX, USA).

RESULTS

The prevalence of MetS was 18.5% in men and 12.9% in women. **Table 1** shows characteristics of the study participants across categories of eating rate. Men who ate fast tended to be young, whereas women who ate slowly tended to be young. Those who ate fast had significantly higher BMI, waist circumference, triglyceride level and

For peer review only - http://bmjopen.bmj.com/site/about/guidelines.xhtml

For peer review only

systolic and diastolic blood pressures and lower HDL-C level in both men and women.

The ORs of the presence of MetS across eating rate are shown in **Table 2**. Faster eating was associated with higher presence of MetS in age- and multivariable-adjusted models. The trend was more apparent in men than in women. The multivariable-adjusted ORs (95% CI) of MetS for eating slow, normal and fast rate were 0.70 (0.62 to 0.79), 1.00 (reference) and 1.61 (1.53 to 1.70), respectively, in men (P for trend <0.001), and 0.74 (0.60 to 0.91), 1.00 (reference) and 1.27 (1.13 to 1.43), respectively, in women (P for trend <0.001). Further adjustment for BMI markedly attenuated these associations; however, the association with fast eating and MetS remained statistically significant in men.

Table 3 shows the ORs of the presence of individual MetS components across three categories of eating rate. The ORs of central obesity increased from slow to fast eating in both men and women (OR 0.63, 1.00 (reference) and 1.97, respectively, in men (P for trend <0.001), and 0.73, 1.00(reference) and 1.44, respectively, in women (P for trend <0.001)). High blood pressure and triglyceride were positively associated with eating rate in both sexes. High fasting plasma glucose and low HDL-C were associated with slow eating in both sexes, but they were associated with fast eating in men only. Additional adjustment for BMI largely attenuated these associations, but the

For peer review only - http://bmjopen.bmj.com/site/about/guidelines.xhtml

associations of slow eating with high blood pressure (men and women) and hyperglycemia (men) and those of fast eating with abnormal lipid profile (men) remained statistically significant.

DISCUSSION

In this large population of Japanese men and women, we found that eating rate was positively associated with the presence of MetS, especially in men. Of components of MetS, the association with abdominal obesity was strongest. The relationship with blood pressure in both sexes and fasting plasma glucose and lipids in men remained statistically significant even after additional adjustment for BMI. To our best knowledge, the present study is the first to report a positive association between eating rate and MetS defined by using waist circumference.

The present finding for MetS is consistent with that of a study among Korean men reporting that eating rate was positively associated with MetS, which was defined by using BMI instead of waist circumference.[20] As regards MetS components, our study is compatible with some cross-sectional studies showing that eating rate was associated with higher BMI[8-10] and two longitudinal studies showing that eating rate was associated with weight gain.[16, 17] In a Korean study that elucidated the association

For peer review only - http://bmjopen.bmj.com/site/about/guidelines.xhtml

between eating rate and components of MetS for men and women separately, eating rate was associated with obesity, high blood pressure, hyperglycemia and abnormal lipid profile in men, whereas it was associated with only obesity in women.[11] Our results were largely consistent with those in the Korean study (except for blood pressure in women).

Notably, we found that the associations of MetS components with eating rate were largely attenuated after adjustment for BMI, a finding compatible with those of a cross-sectional study in Korea[11] and a prospective study in Japan.[18] This result indicates that obesity is a mediator whereby fast eating deteriorates MetS components. We also found, however, that some associations remained statistically significant even after adjusting for BMI (dyslipidemia with fast eating and hyperglycemia with slow eating in men, and high blood pressure with slow eating in both men and women). Similarly, the above-mentioned Korean study[11] reported that high rate of eating remained an important determinant for low HDL-C and high fasting plasma glucose after adjustment for BMI in men. Therefore, there may be pathways other than weight gain that might underlie the association between eating rate and MetS.

We found that the association between eating rate and MetS was stronger in men than in women, consistent with a previous study in Korea.[11] Such sex difference

For peer review only - http://bmjopen.bmj.com/site/about/guidelines.xhtml

may reflect the difference in actual eating speed between men and women. One study elucidated that women took more bites, smaller bite size and slower bites than men in eating the same amount of doughnut, irrespectively of body size.[26] Another study showed that objectively measured eating speed in men with self-reported slow eating was faster than that in women with self-reported fast eating.[27] Taken together, eating rate may have a greater impact on metabolism in men than that in women.

Although mechanisms whereby eating rate influences metabolism is not fully elucidated, overeating may link fast eating to MetS. Fast eating gives few satiety signal from oral cavity to the brain,[28, 29] induces less satiation and satiety due to a lack of stomach expansion[30] and alters the circulating levels of certain gut hormones.[31, 32] In these pathways, fast eating leads to excess energy intake,[33, 34] resulting in overweight and MetS. Because fast eating has been associated with obesity even after adjusting for total energy intake,[8-11, 14] there may be other pathways. One study showed that interleukin-1β and interleukin-6 were higher among those who ate fast than among those who ate slowly, even after accounting for energy intake and BMI.[35] These cytokines could induce insulin resistance,[36, 37] contributing to high blood pressure via an increased renal sodium and water retention, plasma noradrenalin and sympathetic nervous system activity.[38-40]

For peer review only - http://bmjopen.bmj.com/site/about/guidelines.xhtml

The strengths of our study deserve mention. The present study has large sample size (56,865 participants). In addition, body weight, body height and waist circumference were measured by trained technicians, which increased the validity of our study. Nonetheless, several limitations in the present study merit consideration. First, eating rate was self-reported. However, self-reported eating rate has been shown to be well correlated with friend-reported one[9] or objectively measured one.[27] Second, total energy intake was not available in our study. However, because energy intake is influenced by eating rate and thus may act as a mediator rather than confounder, the adjustment of energy intake could underestimate the association between eating rate and MetS. Moreover, eating rate has been associated with body weight independent of energy intake.[7-10, 13] Third, the study participants were mainly workers in various industries including manufacturing (43.6%), service (27.8%) and transport and telecommunications (9.9%), and these figures are similar to those of national survey.[41] However, information on profession of participants was not available, and thus caution is required when generalize the present finding. Fourth, cross-sectional design precludes any causal inferences about the role of eating rate. Finally, we cannot exclude a possibility of the effects of residual confounding and confounding by unmeasured variables.

For peer review only

248 In conclusion, we found a positive trend association between self-reported
249 eating rate and the presence of MetS in men and women. The association between
250 eating rate and MetS was largely accounted for by the difference of body mass across
251 eating rate. Further research should address whether reducing eating rate prevents
252 obesity and MetS.

For peer review only

For peer review only - http://bmjopen.bmj.com/site/about/guidelines.xhtml

For peer review only

Table1. Characteristics of the study individuals according to eating rates

	Men (n = 41,820)				Women (n = 15,045)			
	Slow	Normal	Fast	P for trend [a]	Slow	Normal	Fast	P for trend [a]
n (%)	2,821 (6.8)	24,893 (59.5)	14,106 (33.7)		1,398 (9.3)	9,893 (65.8)	3,754 (24.9)	
Age (years) [b]	46.9 ± 12.3	46.9 ± 10.9	45.0 ± 10.4	<0.001	43.5 ± 12.5	47.2 ± 11.6	46.7 ± 11.2	<0.001
Walking time, ≥60 min/day (%)	21.8	19.0	20.6	0.004	15.5	15.0	16.1	0.798
Smoking status (%)								
Non-smoker	61.9	55.0	56.6	<0.001	82.9	83.1	80.7	0.572

For peer review only - http://bmjopen.bmj.com/site/about/guidelines.xhtml

Daily consuming <20 cigarettes /day	28.6	34.6	31.3		16.0	15.7	17.6	
Daily consuming ≥20 cigarettes /day	9.5	10.4	12.1		1.1	1.2	1.7	
Alcohol (%)				<0.001				<0.001
Non-drinker	30.2	26.7	26.7		53.4	52.2	49.8	
Drinker <1 *go* /day [d]	33.9	35.7	34.5		34.2	35.9	35.5	
Drinker 1 to <2 *go* /day [d]	24.6	26.3	26.8		9.5	9.5	11.5	
Drinker ≥2 *go* /day [d]	11.3	11.3	12.0		2.9	2.4	3.2	
BMI (kg/m^2)[b]	22.4 ± 3.3	23.4 ± 3.3	24.6 ± 3.7	<0.00	21.0 ± 3.5	21.8 ± 3.5	22.5 ± 3.8	<0.00

For peer review only

For peer review only - http://bmjopen.bmj.com/site/about/guidelines.xhtml

Waist circumference (cm)[b]	80.3 ± 9.2	82.9 ± 9.0	86.0 ± 9.8	<0.001	75.5 ± 9.5	77.7 ± 9.4	79.6 ± 9.8	<0.001
Systolic blood pressure (mm Hg)[b]	123.5 ± 15.5	126.1 ± 15.5	126.7 ± 15.1	<0.001	113.1 ± 16.3	117.3 ± 17.2	117.0 ± 17.2	<0.001
Diastolic blood pressure (mm Hg)[b]	75.2± 11.4	77.3 ± 11.9	78.0 ± 12.0	<0.001	69.1 ± 10.9	71.4 ± 11.5	71.5 ± 11.9	<0.001
Fasting plasma glucose (mg/dL)[c]	91 (85 to 98)	92 (86 to 99)	92 (86 to 99)	0.001	87 (82 to 93)	88 (83 to 94)	88 (83 to 94)	0.001
Triglyceride (mg/dL)[c]	92 (65 to 138)	99 (69 to 148)	107 (73 to 161)	<0.001	63 (48 to 87)	69 (51 to 96)	71 (52 to 101)	<0.001

For peer review only

For peer review only - http://bmjopen.bmj.com/site/about/guidelines.xhtml

HDL-C (mg/dL)[b]	61.3 ± 15.3	59.4 ± 15.0	57.2 ± 14.3	<0.001	71.4 ± 15.3	70.5 ± 15.8	69.3 ± 15.5	<0.001

Cross to sectional survey of 56,865 examinees in All Japan Labor Welfare Foundation, Japan, 2011.

BMI=body mass index; HDL-C=high to density lipoprotein cholesterol.

[a] Linear regression, an extension of the Wilcoxon Rank-Sum test and logistic regression, assigning ordinal number (0 to 2) to eating rate, as appropriate.

[b] Mean ± SD.

[c] medians (interquartile range).

[d] One *go* contains ~25g of ethanol.

For peer review only

Table2. Odds ratios and 95% confidence intervals for metabolic syndrome according to eating rate (n = 56,865)

Eating rate	Men (n = 41,820) Slow	Normal [a]	Fast	P for trend [b]	Women (n = 15,045) Slow	Normal [a]	Fast	P for trend [b]
n (%)	2,821 (6.8)	24,893 (59.5)	14,106 (33.7)		1,398 (9.3)	9,893 (65.8)	3,754 (24.9)	
MetS, n	361	4,180	3,193		116	1,261	547	
Model 1 [c]	0.70 (0.62 to 0.79)	1.00	1.62 (1.53 to 1.71)	<0.001	0.75 (0.61 to 0.92)	1.00	1.27 (1.13 to 1.42)	<0.001
Model 2 [d]	0.70 (0.62 to 0.79)	1.00	1.61 (1.53 to 1.70)	<0.001	0.74 (0.60 to 0.91)	1.00	1.27 (1.13 to 1.43)	<0.001

For peer review only - http://bmjopen.bmj.com/site/about/guidelines.xhtml

Model 3[e]	0.91 (0.80 to 1.04)	1.00	1.10 (1.03 to 1.17)	<0.001	0.88 (0.70 to 1.11)	1.00	0.98 (0.86 to 1.12)	0.714

MetS as defined using the criteria of the Joint Interim Statement : the presence of three or more of the following risk factors: 1) waist circumference ≥90 cm in men and ≥80 cm in women, 2) triglyceride level ≥150 mg/dL (1.7 mmol/L), 3) HDL-C level <40 mg/dL (1.04 mmol/L) in men and <50 mg/dL (1.3 mmol/L) in women, 4) blood pressure ≥130 mm Hg systolic or ≥85 mm Hg diastolic, 5) fasting glucose level ≥100 mg/dL (5.6 mmol/L).

[a] Reference.

[b] Multiple logistic regression, assigning ordinal number (0 to 2) to eating rate.

[c] Adjusted for age.

[d] Adjusted for age, smoking status, alcohol, and regular physical activity.

[e] Adjusted for age, smoking status, alcohol, regular physical activity, and body mass index.

For peer review only

For peer review only - http://bmjopen.bmj.com/site/about/guidelines.xhtml

Table3. Odds ratios and 95% confidence intervals for components of metabolic syndrome according to eating rate (n = 56,865)

Eating rate	Men (n = 41,820)				Women (n = 15,045)			
	Slow	Normal [a]	Fast	P for trend [b]	Slow	Normal [a]	Fast	P for trend [b]
n (%)	2,821 (6.8)	24,893 (59.5)	14,106 (33.7)		1,398 (9.3)	9,893 (65.8)	3,754 (24.9)	
Central obesity [c]								
Model 1 [d]	0.63 (0.56 to 0.71)	1.00	1.98 (1.89 to 2.08)	<0.001	0.73 (0.64 to 0.83)	1.00	1.44 (1.34 to 1.56)	<0.001
Model 2 [e]	0.63 (0.56 to 0.70)	1.00	1.97 (1.88 to 2.07)	<0.001	0.73 (0.64 to 0.83)	1.00	1.44 (1.33 to 1.56)	<0.001

For peer review only - http://bmjopen.bmj.com/site/about/guidelines.xhtml

High blood pressure [f]								
Model 1 [d]	0.75 (0.69 to 0.82)	1.00	1.22 (1.17 to 1.27)	<0.001	0.76 (0.66 to 0.88)	1.00	1.10 (1.01 to 1.21)	<0.001
Model 2 [e]	0.74 (0.68 to 0.81)	1.00	1.20 (1.15 to 1.26)	<0.001	0.76 (0.65 to 0.88)	1.00	1.10 (1.00 to 1.20)	<0.001
Model 3 [g]	0.88 (0.81 to 0.96)	1.00	0.97 (0.93 to 1.02)	0.645	0.85 (0.72 to 0.99)	1.00	0.93 (0.84 to 1.02)	0.923
High fasting plasma glucose [h]								
Model 1 [d]	0.78 (0.71 to 0.87)	1.00	1.17 (1.12 to 1.23)	<0.001	1.03 (0.85 to 1.25)	1.00	1.17 (1.04 to 1.31)	0.035
Model 2 [e]	0.78 (0.71 to	1.00	1.16 (1.11 to	<0.00	1.03 (0.85 to	1.00	1.16 (1.03 to	0.042

For peer review only

	0.87)		1.22)	1	1.25)		1.31)	
Model 3 [g]	0.88 (0.80 to 0.98)	1.00	0.99 (0.94 to 1.05)	0.238	1.14 (0.94 to 1.40)	1.00	1.02 (0.90 to 1.15)	0.536
High triglyceride [i]								
Model 1 [d]	0.88 (0.80 to 0.96)	1.00	1.32 (1.26 to 1.38)	<0.001	0.83 (0.67 to 1.01)	1.00	1.14 (1.01 to 1.28)	0.002
Model 2 [e]	0.90 (0.82 to 0.98)	1.00	1.33 (1.27 to 1.39)	<0.001	0.81 (0.66 to 1.00)	1.00	1.13 (1.01 to 1.27)	0.002
Model 3 [g]	1.08 (0.98 to 1.19)	1.00	1.07 (1.02 to 1.12)	0.121	0.90 (0.73 to 1.11)	1.00	0.98 (0.87 to 1.11)	0.753
Low HDL-C [j]								

For peer review only

Model 1 [c]	0.83 (0.73 to 0.96)	1.00	1.34 (1.26 to 1.43)	<0.001	0.90 (0.74 to 1.09)	1.00	1.11 (0.99 to 1.25)	0.018
Model 2 [d]	0.83 (0.73 to 0.96)	1.00	1.36 (1.28 to 1.45)	<0.001	0.89 (0.74 to 1.08)	1.00	1.12 (1.00 to 1.26)	0.011
Model 3 [g]	0.97 (0.84 to 1.12)	1.00	1.10 (1.03 to 1.18)	0.004	1.00 (0.82 to 1.22)	1.00	0.96 (0.85 to 1.08)	0.500

BMI=body mass index; HDL-C=high to density lipoprotein cholesterol.

[a] Reference.

[b] Multiple logistic regression, assigning ordinal number (0 to 2) to eating rate.

[c] Waist circumference ≥90 cm in men, ≥80 cm in women.

[d] Adjusted for age.

[e] Adjusted for age, smoking status, alcohol, and regular physical activity.

For peer review only

For peer review only - http://bmjopen.bmj.com/site/about/guidelines.xhtml

[f] Blood pressure ≥130 mm Hg for systolic or ≥85 mm Hg for diastolic.

[g] Adjusted for age, smoking status, alcohol, regular physical activity and body mass index.

[h] Fasting plasma glucose ≥100 mg/dL or under medication.

[i] Triglyceride ≥150 mg/dL or under medication.

[j] HDL-C <40 mg/dL in men, <50 mg/dL in women or under medication.

For peer review only

For peer review only - http://bmjopen.bmj.com/site/about/guidelines.xhtml

Acknowledgments

The authors would like to thank Dr. Nobuo Yanagisawa and Dr. Takeshi Kawaguchi for their coordinating the study.

Contributors: SN and KKurotani designed study and drafted the manuscript. SN, NMP, AN, KKuwahara performed the data analysis. MD collected and interpreted the data. All authors have participated in the interpretation of the findings, revised it critically for important intellectual content and approved final version to be published. TM and YN provided administrative, technical and material support.SN and TM are guarantors.

Funding: This study was supported by the Industrial Health Foundation.

Competing interests: All authors have completed the ICMJE uniform disclosure form at www.icmje.org/coi_disclosure.pdf and declare: all authors declare no interests.

Ethical approval: The research protocol was approved by the Ethics Committee of the National Center for Global Health and Medicine and the Ethics Committee of Toho University.

Data sharing: No additional data available.

Transparency: SN affirms that the manuscript is an honest, accurate, and transparent account of the study being reported; that no important aspects of the study have been omitted; and that any discrepancies from the study as planned (and, if relevant,

For peer review only

For peer review only - http://bmjopen.bmj.com/site/about/guidelines.xhtml

registered) have been explained.

For peer review only

For peer review only - http://bmjopen.bmj.com/site/about/guidelines.xhtml

References

1. Eckel RH, Alberti KG, Grundy SM, et al. The metabolic syndrome. *Lancet* 2010;**375**:181-83.

2. Gomez-Martinez S, Martinez-Gomez D, Perez de Heredia F, et al. Eating habits and total and abdominal fat in Spanish adolescents: influence of physical activity. The AVENA study. *J Adolesc Health* 2012;**50**:403-09.

3. Ilanne-Parikka P, Eriksson JG, Lindstrom J, et al. Effect of lifestyle intervention on the occurrence of metabolic syndrome and its components in the Finnish Diabetes Prevention Study. *Diabetes care* 2008;**31**:805-07.

4. Nanri A, Tomita K, Matsushita Y, et al. Effect of six months lifestyle intervention in Japanese men with metabolic syndrome: randomized controlled trial. *J Occup Health* 2012;**54**:215-22.

5. Ferster CB, Nurnberger JI, Levitt EB. The control of eating. 1962. *Obes Res* 1996;**4**:401-10.

6. Hill SW, McCutcheon NB. Eating responses of obese and nonobese humans during dinner meals. *Psychosom Med* 1975;**37**:395-401.

7. Bellack AS. Behavior therapy for weight reduction. *Addict Behav* 1975;**1**:73-82.

8. Otsuka R, Tamakoshi K, Yatsuya H, et al. Eating fast leads to obesity: findings based

For peer review only

For peer review only - http://bmjopen.bmj.com/site/about/guidelines.xhtml

on self-administered questionnaires among middle-aged Japanese men and women. *J Epidemiol* 2006;**16**:117-24.

9. Sasaki S, Katagiri A, Tsuji T, et al. Self-reported rate of eating correlates with body mass index in 18-y-old Japanese women. *Int J Obes Relat Metab Disord* 2003;**27**:1405-10.

10. Maruyama K, Sato S, Ohira T, et al. The joint impact on being overweight of self reported behaviours of eating quickly and eating until full: cross sectional survey. *BMJ* 2008;**337**:a2002.

11. Lee KS, Kim DH, Jang JS, et al. Eating rate is associated with cardiometabolic risk factors in Korean adults. *Nut Metab Cardiovasc Dis* 2013;**23**:635-41.

12. Otsuka R, Tamakoshi K, Yatsuya H, et al. Eating fast leads to insulin resistance: findings in middle-aged Japanese men and women. *Prev Med* 2008;**46**:154-59.

13. Radzeviciene L, Ostrauskas R. Fast eating and the risk of type 2 diabetes mellitus: a case-control study. *Clin Nutr* 2013;**32**:232-35.

14. Ohkuma T, Fujii H, Iwase M, et al. Impact of eating rate on obesity and cardiovascular risk factors according to glucose tolerance status: the Fukuoka Diabetes Registry and the Hisayama Study. *Diabetologia* 2013;**56**:70-77.

15. Shigeta H, Shigeta M, Nakazawa A, et al. Lifestyle, obesity, and insulin resistance.

For peer review only - http://bmjopen.bmj.com/site/about/guidelines.xhtml

Diabetes care 2001;**24**:608.

16. Gerace TA, George VA. Predictors of weight increases over 7 years in fire fighters and paramedics. *Prev Med* 1996;**25**:593-600.

17. Tanihara S, Imatoh T, Miyazaki M, et al. Retrospective longitudinal study on the relationship between 8-year weight change and current eating speed. *Appetite* 2011;**57**:179-83.

18. Sakurai M, Nakamura K, Miura K, et al. Self-reported speed of eating and 7-year risk of type 2 diabetes mellitus in middle-aged Japanese men. *Metabolism* 2012;**61**:1566-71.

19. Kral JG, Buckley MC, Kissileff HR, et al. Metabolic correlates of eating behavior in severe obesity. *Int J Obes Relat Metab Disord* 2001;**25**:258-64.

20. Shin A, Lim SY, Sung J, et al. Dietary intake, eating habits, and metabolic syndrome in Korean men. *J Am Diet Assoc* 2009;**109**:633-40.

21. Shen W, Punyanitya M, Chen J, et al. Waist circumference correlates with metabolic syndrome indicators better than percentage fat. *Obesity (Silver Spring)* 2006;**14**:727-36.

22. Alberti KG, Eckel RH, Grundy SM, et al. Harmonizing the metabolic syndrome: a joint interim statement of the International Diabetes Federation Task Force on Epidemiology and Prevention; National Heart, Lung, and Blood Institute; American

For peer review only - http://bmjopen.bmj.com/site/about/guidelines.xhtml

For peer review only

Heart Association; World Heart Federation; International Atherosclerosis Society; and International Association for the Study of Obesity. *Circulation* 2009;**120**:1640-5.

23. Ministry of Justice, Japan. Industrial Safty and Health Act, Article 66 (Medical Examination). Available at: http://www.japaneselawtranslation.go.jp/law/detail/?id=1926&vm=&re=. Accessed 26 May 2014.

24. Ministry of Health, Labour and Welfare. Specific Health Checkups and Specific Health Guidance. Secondary Specific Health Checkups and Specific Health Guidance. Available at: http://www.mhlw.go.jp/english/wp/wp-hw3/dl/2-007.pdf. Accessed 26 May 2014.

25. Ministry of Health, Labour and Welfare. Questionnaire of Specific Health Checkups. Secondary Questionnaire of Specific Health Checkups. Available at: http://www.mhlw.go.jp/seisakunitsuite/bunya/kenkou_iryou/kenkou/seikatsu/dl/hoken-program2_02.pdf. Accessed 26 May 2014. (in Japanese)

26. Hill SW, McCutcheon NB. Contributions of obesity, gender, hunger, food preference, and body size to bite size, bite speed, and rate of eating. *Appetite* 1984;**5**:73-83.

27. Ekuni D, Furuta M, Takeuchi N, et al. Self-reports of eating quickly are related to a decreased number of chews until first swallow, total number of chews, and total

For peer review only - http://bmjopen.bmj.com/site/about/guidelines.xhtml

duration of chewing in young people. *Arch Oral Biol* 2012;**57**:981-86.

28. Rolls ET. Sensory processing in the brain related to the control of food intake. *Proc Nutr Soc* 2007;**66**:96-112.

29. Morton GJ, Cummings DE, Baskin DG, et al. Central nervous system control of food intake and body weight. *Nature* 2006;**443**:289-95.

30. de Graaf C, Blom WA, Smeets PA, et al. Biomarkers of satiation and satiety. *Am J Clin Nutr* 2004;**79**:946-61.

31. Kokkinos A, le Roux CW, Alexiadou K, et al. Eating slowly increases the postprandial response of the anorexigenic gut hormones, peptide YY and glucagon-like peptide-1. *J Clin Endocrinol Metab* 2010;**95**:333-37.

32. Sobki SH, Zaid AA, Khan HA, et al. Significant impact of pace of eating on serum ghrelin and glucose levels. *Clin Biochem* 2010;**43**:522-24.

33. Viskaal-van Dongen M, Kok FJ, de Graaf C. Eating rate of commonly consumed foods promotes food and energy intake. *Appetite* 2011;**56**:25-31.

34. Scisco JL, Muth ER, Dong Y, et al. Slowing bite-rate reduces energy intake: an application of the bite counter device. *J Am Diet Assoc* 2011;**111**:1231-35.

35. Mochizuki K, Misaki Y, Miyauchi R, et al. A higher rate of eating is associated with higher circulating interluekin-1beta concentrations in Japanese men not being treated for

For peer review only - http://bmjopen.bmj.com/site/about/guidelines.xhtml

metabolic diseases. *Nutrition* 2012;**28**:978-83.

36. Besedovsky H, del Rey A, Sorkin E, et al. Immunoregulatory feedback between interleukin-1 and glucocorticoid hormones. *Science* 1986;**233**:652-54

37. Matsuki T, Horai R, Sudo K, et al. IL-1 plays an important role in lipid metabolism by regulating insulin levels under physiological conditions. *J Exp Med* 2003;**198**:877-88.

38. Yatabe MS, Yatabe J, Yoneda M, et al. Salt sensitivity is associated with insulin resistance, sympathetic overactivity, and decreased suppression of circulating renin activity in lean patients with essential hypertension. *Am J Clin Nutr* 2010;**92**:77-82.

39. Facchini FS, DoNascimento C, Reaven GM, et al. Blood pressure, sodium intake, insulin resistance, and urinary nitrate excretion. *Hypertension* 1999;**33**:1008-12.

40. Pollare T, Lithell H, Berne C. Insulin resistance is a characteristic feature of primary hypertension independent of obesity. *Metabolism*. 1990;**39**:167-74.

41. Ministry of Health, Labour and Welfare. Special Survey on Industrial Safety and Health. Available at: http://www.mhlw.go.jp/bunya/roudoukijun/anzeneisei11/h23.html. Accessed 26 May 2014. (in Japanese)

For peer review only - http://bmjopen.bmj.com/site/about/guidelines.xhtml

STROBE Statement—Checklist of items that should be included in reports of ***cross-sectional studies***

MS ID#: bmjopen-2014-005241.R1
MS TITLE: Self-reported eating rate and metabolic syndrome in Japanese: cross-sectional study

	Item No	Recommendation	Location in manuscript
**Title and abstract**	1	(*a*) Indicate the study's design with a commonly used term in the title or the abstract	Line 1 on page 1 and line 23 on page 3
		(*b*) Provide in the abstract an informative and balanced summary of what was done and what was found	What was done: line 21-22 on page 3<br>What was found: line 42-44 on page 4
**Introduction**			
Background/rationale	2	Explain the scientific background and rationale for the investigation being reported	Scientific background: line 51-68 on page 6 and 7<br>Rationale: Line 68-74 on page 7
Objectives	3	State specific objectives, including any prespecified hypotheses	Line 74-78 on page 7
**Methods**			
Study design	4	Present key elements of study design early in the paper	Line 75 on page 7
Setting	5	Describe the setting, locations, and relevant dates, including periods of recruitment, exposure, follow-up, and data collection	Line 84-88 on page 7-8
Participants	6	(*a*) Give the eligibility criteria, and the sources and methods of selection of participants	Line 88-95 on page 8
Variables	7	Clearly define all outcomes, exposures, predictors, potential confounders, and effect modifiers. Give diagnostic criteria, if applicable	Outcome: line 122-128 on page 10<br>Exposure: line 105-107 on page 9<br>Potential confounders: line 140-147 on page 11
Data sources/ measurement	8*	For each variable of interest, give sources of data and details of methods of assessment (measurement). Describe comparability of assessment methods if there is more than one group	Line 102-120 on page 8 and 9
Bias	9	Describe any efforts to address potential sources of bias	Exclusion: line 89-90 on page 8<br>Adjustment: line 140-147 on page 11
Study size	10	Explain how the study size was arrived at	Not provided
Quantitative variables	11	Explain how quantitative variables were handled in the analyses. If applicable, describe which groupings were chosen and why	Adjustment: line 140-147 on page 11

For peer review only - http://bmjopen.bmj.com/site/about/guidelines.xhtml

For peer review only

Statistical methods	12	(*a*) Describe all statistical methods, including those used to control for confounding	Line 130-151 on page 10-11
		(*b*) Describe any methods used to examine subgroups and interactions	Line 146-149 on page 11
		(*c*) Explain how missing data were addressed	We excluded participants who had missing information on potential confounding variables (line 90 on page 8).
		(*d*) If applicable, describe analytical methods taking account of sampling strategy	N/A
		(*e*) Describe any sensitivity analyses	N/A
**Results**			
Participants	13*	(a) Report numbers of individuals at each stage of study—eg numbers potentially eligible, examined for eligibility, confirmed eligible, included in the study, completing follow-up, and analysed	Line 159-160 on page 11<br>Table 1
		(b) Give reasons for non-participation at each stage	N/A
		(c) Consider use of a flow diagram	N/A
Descriptive data	14*	(a) Give characteristics of study participants (eg demographic, clinical, social) and information on exposures and potential confounders	Table 1<br>Line 154-178 on page 12 and 13
		(b) Indicate number of participants with missing data for each variable of interest	N/A
Outcome data	15*	Report numbers of outcome events or summary measures	Table 2<br>Line 154 on page 11
Main results	16	(*a*) Give unadjusted estimates and, if applicable, confounder-adjusted estimates and their precision (eg, 95% confidence interval). Make clear which confounders were adjusted for and why they were included	Table 2, Table3<br>Line 159- on page 11-12
		(*b*) Report category boundaries when continuous variables were categorized	Table2, Table3
		(*c*) If relevant, consider translating estimates of relative risk into absolute risk for a meaningful time period	N/A
Other analyses	17	Report other analyses done—eg analyses of subgroups and interactions, and sensitivity analyses	Line 147-149 on page 11
**Discussion**			
Key results	18	Summarise key results with reference to study objectives	Line 181-185 on page 13
Limitations	19	Discuss limitations of the study, taking into account sources of potential bias	Line 233-247 on page 16

For peer review only - http://bmjopen.bmj.com/site/about/guidelines.xhtml

		or imprecision. Discuss both direction and magnitude of any potential bias	
Interpretation	20	Give a cautious overall interpretation of results considering objectives, limitations, multiplicity of analyses, results from similar studies, and other relevant evidence	Line 188-209 on page 13-14
Generalisability	21	Discuss the generalisability (external validity) of the study results	Line 243-244 on page 16
**Other information**			
Funding	22	Give the source of funding and the role of the funders for the present study and, if applicable, for the original study on which the present article is based	This study was supported by the Industrial Health Foundation for drafting the maniscript.

N/A: Not applicable.

*Give information separately for exposed and unexposed groups.

Note: An Explanation and Elaboration article discusses each checklist item and gives methodological background and published examples of transparent reporting. The STROBE checklist is best used in conjunction with this article (freely available on the Web sites of PLoS Medicine at http://www.plosmedicine.org/, Annals of Internal Medicine at http://www.annals.org/, and Epidemiology at http://www.epidem.com/). Information on the STROBE Initiative is available at www.strobe-statement.org.

For peer review only - http://bmjopen.bmj.com/site/about/guidelines.xhtml

BMJ Open

Self-reported eating rate and metabolic syndrome in Japanese: cross-sectional study

Journal:	*BMJ Open*
Manuscript ID:	bmjopen-2014-005241.R2
Article Type:	Research
Date Submitted by the Author:	31-Jul-2014
Complete List of Authors:	Nagahama, Satsue; All Japan Labor Welfare Foundation, ; School of Medicine, Toho University, Department of Environmental and Occupational Health<br>Kurotani, Kayo; National Center for Global Health and Medicine, Department of Epidemiology and Prevention, Center for Clinical Sciences<br>Pham, Ngoc Minh; Thai Nguyen University of Medicine and Pharmacy, Department of Epidemiology, Faculty of Public Health<br>Nanri, Akiko; National Center for Global Health and Medicine, Department of Epidemiology and Prevention, Center for Clinical Sciences<br>Kuwahara, Keisuke; National Center for Global Health and Medicine, Department of Epidemiology and Prevention, Center for Clinical Sciences<br>Dan, Masashi; All Japan Labor Welfare Foundation,<br>Nishiwaki, Yuji; School of Medicine, Toho University, Department of Environmental and Occupational Health<br>Mizoue, Tetsuya; National Center for Global Health and Medicine, Department of Epidemiology and Prevention, Center for Clinical Sciences
**Primary Subject Heading**:	Epidemiology
Secondary Subject Heading:	Epidemiology
Keywords:	EPIDEMIOLOGY, PREVENTIVE MEDICINE, PUBLIC HEALTH

SCHOLARONE™
Manuscripts

Self-reported eating rate and metabolic syndrome in Japanese: cross-sectional study

Satsue Nagahama [1 2 3*], Kayo Kurotani [1], Ngoc Minh Pham [4], Akiko Nanri [1], Keisuke Kuwahara [1], Masashi Dan [2], Yuji Nishiwaki [3], Tetsuya Mizoue [1]

[1] Department of Epidemiology and Prevention, Center for Clinical Sciences, National Center for Global Health and Medicine, 1-21-1, Toyama, Shinjuku-ku, Tokyo, Japan, Zip162-8655; Tel +81-3-3202-7181, Fax 81-3-3202-7364,

E-mail: satsue_0323@hotmail.com

[2] All Japan Labor Welfare Foundation, Tokyo, Japan;

[3] Department of Environmental and Occupational Health, School of Medicine, Toho University, Tokyo, Japan;

[4] Department of Epidemiology, Faculty of Public Health, Thai Nguyen University of Medicine and Pharmacy, Thai Nguyen Province, Vietnam;

Keywords: eating rate, metabolic syndrome, health checkup, Japan, Joint Interim Statement

For peer review only

Word count: 2875

For peer review only

For peer review only - http://bmjopen.bmj.com/site/about/guidelines.xhtml

ABSTRACT

Objectives: To examine the association between self-reported eating rate and metabolic syndrome.

Design: Cross-sectional study.

Setting: Annual health checkup at a health check service center in Japan.

Participants: A total of 56,865 participants (41,820 males and 15,045 females) who attended health checkup in 2011 and reported not to have a history of coronary heart disease or stroke.

Main outcome measure: Metabolic syndrome was defined by the joint of interim statement of the International Diabetes Federation and the American Heart Association/National Heart, Lung, and Blood Institute.

Results: In multiple logistic regression models, eating rate was significantly and positively associated with metabolic syndrome. The multivariable-adjusted odds ratios (95% confidence interval) for slow, normal and fast were 0.70 (0.62 to 0.79), 1.00 (reference) and 1.61 (1.53 to 1.70), respectively, in men (P for trend <0.001), and 0.74 (0.60 to 0.91), 1.00 (reference) and 1.27 (1.13 to 1.43), respectively, in women (P for trend <0.001). Of metabolic syndrome components, abdominal obesity showed the strongest association with eating rate. The associations of eating rate and metabolic

For peer review only - http://bmjopen.bmj.com/site/about/guidelines.xhtml

syndrome and its components were largely attenuated after further adjustment for body mass index; however, the association of slow eating with lower odds of high blood pressure (men and women) and hyperglycemia (men) and that of fast eating with higher odds of lipid abnormality (men) remained statistically significant.

Conclusions: Results suggest that eating rate is associated with the presence of metabolic syndrome and that this association is largely accounted for by the difference of body mass according to eating rate.

For peer review only

For peer review only - http://bmjopen.bmj.com/site/about/guidelines.xhtml

Strength and Limitation

This study included a large number of participants, used waist circumference in defining metabolic syndrome, and analyzed data for men and women separately.

Eating rate was assessed by a self-reported questionnaire. Information on dietary intake was not obtained.

For peer review only

For peer review only - http://bmjopen.bmj.com/site/about/guidelines.xhtml

Introduction

Metabolic syndrome (MetS) is a cluster of physiological risk factors associated with cardiovascular disease and several types of cancer.[1] Determination of etiologic factors for MetS is required for the establishment of public health strategies to reduce its prevalence and prevent resulting complications. Growing evidence from both observational and interventional studies suggests a role of dietary habits in the development of MetS,[2-4] which originates from obesity. Obesity has been extensively investigated in relation to dietary habits including eating rate since 1962, when Ferster published a theoretical and practical weight control program focusing on eating behaviors including eating rate.[5] Observational studies showed that obese people ate at a faster rate than non-obese people,[6] and reducing eating rate may be a simple and effective therapy for obesity.[7]

During the past decade, several cross-sectional studies have found a positive association between eating rate and overweight[8-11] or insulin resistance.[11-15] Similarly, a few longitudinal studies showed that eating fast was associated with an increased risk of weight gain[16, 17] and type 2 diabetes.[18] In addition, some cross-sectional studies have reported that fast eating was positively associated with hypertriglyceridemia and low high-density lipoprotein cholesterol (HDL-C).[11, 14, 19] Therefore it is conceivable that

For peer review only

eating rate may be associated with MetS. To our knowledge, however, only one Korean study cross-sectionally examined eating rate in relation to MetS.[20] In that study, MetS was defined by using body mass index (BMI), rather than waist circumference, and investigated the association in men only. Waist circumference is a component of most MetS definitions as a surrogate of central obesity, which can better predict cardiovascular risk.[21] It is therefore necessary to examine the relationship between eating rate and MetS using waist circumference in both men and women. Here, we investigated cross-sectionally the association between self-reported eating rate and the presence of MetS according to the joint of interim statement of the International Diabetes Federation and the American Heart Association/National Heart, Lung, and Blood Institute (JIS)[22] using a large dataset of health checkup in Japanese men and women.

METHODS AND PROCEDURES

Study population

In Japan, health checkup under occupational health and safety law is mandatory for all employed workers[23] and has been modified in 2008 when the recommendation for new national health checkup system focusing on MetS has been launched.[24] Study

For peer review only - http://bmjopen.bmj.com/site/about/guidelines.xhtml

participants were attendants of 2011 (calendar year) annual health checkup at All Japan Labor Welfare Foundation (Tokyo), a health service center performing health checkup. Participants were mainly Japanese employees but also included a small number of their dependents and foreign workers, aged 17-99 in men and 17-85 in women. Of 297,148 participants, we excluded 3,660 with a history of myocardial infarction, coronary heart disease or stroke, which might influence both eating rate and MetS. Of the remaining 293,488 participants, we included 269,297 who reported their eating rate. Of these, we excluded 182,487 with missing data for any of the components of MetS (173,376 without plasma glucose, 61,602 without waist circumference, 43,724 without triglyceride, 43,401 without HDL-C and 504 without blood pressure; some participants had two or more missing data). Major reason for a large number of participants with missing measurement of blood glucose was that HbA1c was measured instead of blood glucose for those who attended checkup in non-fasting condition. Of the remaining 86,810 participants, we further excluded 29,337 who took meal within 8 hours before blood drawing or provided no information on meal time. After further exclusion of 608 participants with missing information on covariates (BMI, smoking status, alcohol consumption and physical activity), 56,865 participants (41,820 males and 15,045 females) remained for analysis. We did not obtain written informed consent from each

For peer review only - http://bmjopen.bmj.com/site/about/guidelines.xhtml

participant; instead, we disclosed the execution of the study by showing posters, giving participants an opportunity to refuse the use of their data for the study. In Japan, informed consent is not necessarily required for observational studies using existing data, as described in the Japanese Ethical Guidelines for Epidemiological Research.[25] The research protocol was approved by the Ethics Committee of the National Center for Global Health and Medicine and the Ethics Committee of Toho University.

Data collection and measurements

A self-administered questionnaire, which was recommended for specific health examination by the Japanese government (Ministry of Health, Labour and Welfare),[26] was used to assess eating rate, medical history and health-related lifestyles including smoking, alcohol consumption and regular physical activity. Eating rate was assessed by asking "How fast is your speed of eating?", with three response options (slow, normal and fast). A trained staff measured height to the nearest 0.1 cm, weight to the nearest 0.1 kg and waist circumference to the nearest 0.1 cm at the umbilical level in a standing position. BMI was calculated as the weight in kilograms divided by the squared height in meters. Blood pressure in the sitting position was measured using an automated machine (HEM-907, Omron, Kyoto, Japan). Participants with high blood pressure (≥130 mm Hg systolic or ≥85 mm Hg diastolic) received another measurement and data

For peer review only - http://bmjopen.bmj.com/site/about/guidelines.xhtml

showing lower systolic blood pressure was used. Venous blood sample was collected, stored in a cooler at 4 degrees for transportation to an external laboratory (SRL, Tokyo, Japan) and measured within 24 hours of blood drawing. Triglyceride level was measured by enzymatic colorimertic test (Bio Majesty JCA-BM8060, JEOL, Tokyo, Japan). HDL-C level was determined by a direct method (Bio Majesty JCA-BM8060, JEOL, Tokyo, Japan). Plasma glucose level was determined using by the hexokinase method (an automatic clinical chemistry analyzer JCA-BM9000 series, JEOL, Tokyo, Japan).

Definitions for MetS

According to the JIS criteria, MetS was defined as three or more of the following risk factors: 1) waist circumference for Asian population ≥90 cm in men and ≥80 cm in women, 2) triglyceride level ≥150 mg/dL (1.7 mmol/L), 3) HDL-C level <40 mg/dL (1.04 mmol/L) in men and <50 mg/dL (1.3 mmol/L) in women, 4) blood pressure ≥130 mm Hg systolic or ≥85 mm Hg diastolic, 5) fasting glucose level ≥100 mg/dL (5.6 mmol/L).[22] Participants under medication for diabetes, hypertension and dyslipidemia were considered as having respective factor, irrespective of measured data.

Statistical analysis

Participants were divided into three groups according to eating rate (slow,

For peer review only - http://bmjopen.bmj.com/site/about/guidelines.xhtml

normal and fast). The characteristics of participants across eating rate categories were expressed as means (standard deviation) for continuous variables and percentages for categorical variables, respectively. Fasting plasma glucose and triglyceride were highly skewed; hence, they were log-transformed and expressed as geometric means (95% confidence intervals (CI)). Trend association was assessed by assigning ordinal numbers (0 to 2) to the categories of eating rate (slow, normal and fast, respectively) and was tested using linear regression and logistic regression, as appropriate. Multiple logistic regression analysis was used to estimate the odds ratios (OR) with 95% CI for the presence of MetS across eating rate categories, with normal eating rate as the reference. We adjusted for age (continuous, year) in the basic model. In the second model, we further adjusted for smoking status (non-smoker, daily smoker consuming <20 cigarettes per day or ≥20 cigarettes per day), physical activity (walking time <60 min per day or ≥60 min per day), and alcohol consumption (non-drinker, <1 *go*, 1 to <2 *go* or ≥2 *go* per day; one *go* of sake, Japanese traditional beverage, is about 180 ml of 10 to 14% of ethanol and contains ~23g of ethanol). In the third model, we added BMI (continuous, kg/m^2) to the second model. We performed likelihood ratio test for testing the interaction between eating rate and sex. All analyses were done for men and women separately because the interaction was significant (P for interaction <0.001). We

For peer review only

For peer review only - http://bmjopen.bmj.com/site/about/guidelines.xhtml

repeated the above analyses for each component of MetS. Two-tailed P value <0.05 was considered statistically significant. All statistical analyses were performed with STATA, version 12.1 (StataCorp, College Station, TX, USA).

RESULTS

The prevalence of MetS was 18.5% in men and 12.8 % in women. **Table 1** shows characteristics of the study participants across categories of eating rate. Men who ate fast tended to be young, whereas women who ate slowly tended to be young. Both men and women who ate fast consumed greater amount of alcohol and had significantly higher BMI, waist circumference, triglyceride level and systolic and diastolic blood pressures and lower HDL-C level than those who ate slowly.

The ORs of the presence of MetS across eating rate are shown in **Table 2**. Faster eating was associated with higher presence of MetS in age- and multivariable-adjusted models. The trend was more apparent in men than in women. The multivariable-adjusted ORs (95% CI) of MetS for eating slow, normal and fast rate were 0.70 (0.62 to 0.79), 1.00 (reference) and 1.61 (1.53 to 1.70), respectively, in men (P for trend <0.001), and 0.74 (0.60 to 0.91), 1.00 (reference) and 1.27 (1.13 to 1.43), respectively, in women (P for trend <0.001). Further adjustment for BMI markedly

For peer review only - http://bmjopen.bmj.com/site/about/guidelines.xhtml

attenuated these associations; however, the association with fast eating and MetS remained statistically significant in men.

Table 3 shows the ORs of the presence of individual MetS components across three categories of eating rate. Central obesity sharply increased with increasing speed of eating; the ORs for slow, normal and fast eating were 0.63, 1.00 (reference) and 1.97, respectively, in men (P for trend <0.001), and 0.73, 1.00(reference) and 1.44, respectively, in women (P for trend <0.001). High blood pressure and high triglyceride were positively associated with eating rate in both men and women. High fasting plasma glucose and low HDL-C were associated with fast eating in both men and women, but they were associated with slow eating in men only. Additional adjustment for BMI largely attenuated these associations and the significant trend association disappeared, but the associations of slow eating with decreased odds of high blood pressure (men and women) and hyperglycemia (men) and those of fast eating with increased odds of abnormal lipid profile (men) remained statistically significant.

DISCUSSION

In this large population of Japanese men and women, we found that eating rate was positively associated with the presence of MetS, especially in men. Of components

For peer review only - http://bmjopen.bmj.com/site/about/guidelines.xhtml

of MetS, the association with abdominal obesity was strongest. These associations were largely attenuated after adjustment for BMI. However, slow eating was associated with decreased odds of high blood pressure in both men and women and high fasting plasma glucose in men, and fast eating was associated with increased odds of lipid abnormal profiles in men. To our best knowledge, the present study is the first to report a positive association between eating rate and MetS defined by using waist circumference.

The present finding for MetS is consistent with that of a study among Korean men reporting that eating rate was positively associated with MetS, which was defined by using BMI instead of waist circumference.[20] As regards MetS components, our study is compatible with some cross-sectional studies showing that eating rate was associated with higher BMI[8-11] and two longitudinal studies showing that eating rate was associated with weight gain.[16, 17] In a Korean study that elucidated the association between eating rate and components of MetS for men and women separately, eating rate was associated with obesity, high blood pressure, hyperglycemia and abnormal lipid profile in men, whereas it was associated with only obesity in women.[11] Our results were largely consistent with those in the Korean study (except for blood pressure in women).

For peer review only - http://bmjopen.bmj.com/site/about/guidelines.xhtml

Notably, we found that the associations of MetS components with eating rate were largely attenuated after adjustment for BMI, a finding compatible with those of a cross-sectional study in Korea[11] and a prospective study in Japan.[18] This result indicates that obesity is a mediator whereby fast eating deteriorates MetS components. We also found, however, that some associations remained statistically significant even after adjusting for BMI (dyslipidemia with fast eating and hyperglycemia with slow eating in men, and high blood pressure with slow eating in both men and women). Similarly, the above-mentioned Korean study[11] reported that high rate of eating remained an important determinant for low HDL-C and high fasting plasma glucose after adjustment for BMI in men. Therefore, there may be pathways other than weight gain that might underlie the association between eating rate and MetS.

We found that the association between eating rate and MetS was stronger in men than in women, consistent with a previous study in Korea.[11] Such sex difference may reflect the difference in actual eating speed between men and women. One study elucidated that women took more bites, smaller bite size and slower bites than men in eating the same amount of doughnut, irrespectively of body size.[27] Another study showed that objectively measured eating speed in men with self-reported slow eating

For peer review only

For peer review only - http://bmjopen.bmj.com/site/about/guidelines.xhtml

was faster than that in women with self-reported fast eating.[28] Taken together, eating rate may have a greater impact on metabolism in men than that in women.

Although mechanisms whereby eating rate influences metabolism is not fully elucidated, overeating may link fast eating to MetS. Fast eating gives few satiety signal from oral cavity to the brain,[29, 30] induces less satiation and satiety due to a lack of stomach expansion[31] and alters the circulating levels of certain gut hormones.[32, 33] In these pathways, fast eating leads to excess energy intake,[34, 35] resulting in overweight and MetS. Because fast eating has been associated with obesity even after adjusting for total energy intake,[8-11, 14] there may be other pathways. One study showed that interleukin-1β and interleukin-6 were higher among those who ate fast than among those who ate slowly, even after accounting for energy intake and BMI.[36] These cytokines could induce insulin resistance,[37, 38] contributing to high blood pressure via an increased renal sodium and water retention, plasma noradrenalin and sympathetic nervous system activity.[39-41]

The strengths of our study deserve mention. The present study has large sample size (56,865 participants). In addition, body weight, body height and waist circumference were measured by trained technicians, which increased the validity of our study. Nonetheless, several limitations in the present study merit consideration. First,

For peer review only - http://bmjopen.bmj.com/site/about/guidelines.xhtml

eating rate was self-reported. However, self-reported eating rate has been shown to be well correlated with friend-reported one[9] or objectively measured one.[28] Second, information on dietary intake was not available in the present study and thus total energy intake was not considered in analyses. The adjustment of energy intake, however, may not be appropriate because energy intake may increase with eating rate and thus may act as a mediator rather than confounder. Moreover, eating rate has been associated with body weight independent of energy intake.[7-10,13] Third, fast-food is an energy-dense dietary source and has been linked to MetS.[42] Because fast-food is usually consumed quickly, it may confound the association of eating rate with MetS. Fourth, the study participants were mainly workers in various industries including manufacturing (43.6%), service (27.8%) and transport and telecommunications (9.9%), and these figures are similar to those of national survey.[43] However, information on profession of participants was not available, and thus caution is required when generalize the present finding. Fifth, a large number of participants were excluded from the present analysis due to missing data for MetS components. We cannot deny a possibility of bias due to such selective inclusion. Sixth, cross-sectional design precludes any causal inferences about the role of eating rate. Finally, we cannot exclude a possibility of the effects of residual confounding and confounding by unmeasured variables.

For peer review only - http://bmjopen.bmj.com/site/about/guidelines.xhtml

For peer review only

In conclusion, we found a positive trend association between self-reported eating rate and the presence of MetS in men and women. The association between eating rate and MetS was largely accounted for by the difference of body mass across eating rate. Further research should address whether reducing eating rate prevents obesity and MetS.

For peer review only

For peer review only - http://bmjopen.bmj.com/site/about/guidelines.xhtml

Table1. Characteristics of the study individuals according to eating rates

	Men (n = 41,820)			P for trend [a]	Women (n = 15,045)			P for trend [a]
	Slow	Normal	Fast		Slow	Normal	Fast	
n (%)	2,821 (6.8)	24,893 (59.5)	14,106 (33.7)		1,398 (9.3)	9,893 (65.8)	3,754 (24.9)	
Age (years) [b]	46.9 ± 12.3	46.9 ± 10.9	45.0 ± 10.4	<0.001	43.5 ± 12.5	47.2 ± 11.6	46.7 ± 11.2	<0.001
Walking time, ≥60 min/day (%)	21.8	19.0	20.6	0.004	15.5	15.0	16.1	0.798
Smoking status (%)								
Non-smoker	61.9	55.0	56.6	<0.001	82.9	83.1	80.7	0.572
Daily consuming <20 cigarettes /day	28.6	34.6	31.3		16.0	15.7	17.6	
Daily consuming ≥20 cigarettes /day	9.5	10.4	12.1		1.1	1.2	1.7	

For peer review only - http://bmjopen.bmj.com/site/about/guidelines.xhtml

Alcohol (%)								
Non-drinker	30.2	26.7	26.7	<0.001	53.4	52.2	49.8	<0.001
Drinker <1 *go* /day [d]	33.9	35.7	34.5		34.2	35.9	35.5	
Drinker 1 to <2 *go* /day [d]	24.6	26.3	26.8		9.5	9.5	11.5	
Drinker ≥2 *go* /day [d]	11.3	11.3	12.0		2.9	2.4	3.2	
BMI (kg/m^2)[b]	22.4 ± 3.3	23.4 ± 3.3	24.6 ± 3.7	<0.001	21.0 ± 3.5	21.8 ± 3.5	22.5 ± 3.8	<0.001
Waist circumference (cm)[b]	80.3 ± 9.2	82.9 ± 9.0	86.0 ± 9.8	<0.001	75.5 ± 9.5	77.7 ± 9.4	79.6 ± 9.8	<0.001
Systolic blood pressure (mm Hg)[b]	123.5 ± 15.5	126.1 ± 15.5	126.7 ± 15.1	<0.001	113.1 ± 16.3	117.3 ± 17.2	117.0 ± 17.2	<0.001
Diastolic blood pressure (mm Hg)[b]	75.2 ± 11.4	77.3 ± 11.9	78.0 ± 12.0	<0.001	69.1 ± 10.9	71.4 ± 11.5	71.5 ± 11.9	<0.001
Fasting plasma glucose (mg/dL)[c]	93.0 (92.5 to 93.6)	94.4 (94.2 to 94.6)	94.6 (94.3 to 94.8)	<0.001	88.1 (87.5 to 88.7)	89.1 (88.9 to 89.3)	89.5 (89.1 to 89.9)	<0.001

For peer review only

For peer review only - http://bmjopen.bmj.com/site/about/guidelines.xhtml

Triglyceride (mg/dL)[c]	98.3 (96.2 to 100.4)	103.8 (103.0 to 104.6)	110.8 (109.8 to 111.9)	<0.001	67.0 (65.3 to 68.7)	71.6 (70.9 to 72.3)	74.1 (72.9 to 75.2)	<0.001
HDL-C (mg/dL)[b]	61.3 ± 15.3	59.4 ± 15.0	57.2 ± 14.3	<0.001	71.4 ± 15.3	70.5 ± 15.8	69.3 ± 15.5	<0.001

Cross to sectional survey of 56,865 examinees in All Japan Labor Welfare Foundation, Japan, 2011.

BMI=body mass index; HDL-C=high to density lipoprotein cholesterol.

[a] Linear regression and logistic regression, assigning ordinal number (0 to 2) to eating rate, as appropriate.

[b] Mean ± SD.

[c] Geometric means (95% confidence intervals).

[d] One *go* contains ~25g of ethanol.

For peer review only

For peer review only - http://bmjopen.bmj.com/site/about/guidelines.xhtml

Table2. Odds ratios and 95% confidence intervals for metabolic syndrome according to eating rate (n = 56,865)

	Men (n = 41,820)				Women (n = 15,045)			
Eating rate	Slow	Normal [a]	Fast	P for trend [b]	Slow	Normal [a]	Fast	P for trend [b]
n (%)	2,821 (6.8)	24,893 (59.5)	14,106 (33.7)		1,398 (9.3)	9,893 (65.8)	3,754 (24.9)	
MetS, n	361	4,180	3,193		116	1,261	547	
Model 1 [c]	0.70 (0.62 to 0.79)	1.00	1.62 (1.53 to 1.71)	<0.001	0.75 (0.61 to 0.92)	1.00	1.27 (1.13 to 1.42)	<0.001
Model 2 [d]	0.70 (0.62 to 0.79)	1.00	1.61 (1.53 to 1.70)	<0.001	0.74 (0.60 to 0.91)	1.00	1.27 (1.13 to 1.43)	<0.001
Model 3 [e]	0.91 (0.80 to 1.04)	1.00	1.10 (1.03 to 1.17)	<0.001	0.88 (0.70 to 1.11)	1.00	0.98 (0.86 to 1.12)	0.714

MetS=Metabolic syndrome.

For peer review only - http://bmjopen.bmj.com/site/about/guidelines.xhtml

MetS as defined using the criteria of the Joint Interim Statement : the presence of three or more of the following risk factors: 1) waist circumference ≥90 cm in men and ≥80 cm in women, 2) triglyceride level ≥150 mg/dL (1.7 mmol/L), 3) HDL-C level <40 mg/dL (1.04 mmol/L) in men and <50 mg/dL (1.3 mmol/L) in women, 4) blood pressure ≥130 mm Hg systolic or ≥85 mm Hg diastolic, 5) fasting glucose level ≥100 mg/dL (5.6 mmol/L).

[a] Reference.

[b] Multiple logistic regression, assigning ordinal number (0 to 2) to eating rate.

[c] Adjusted for age.

[d] Adjusted for age, smoking status, alcohol and regular physical activity.

[e] Adjusted for age, smoking status, alcohol, regular physical activity and body mass index.

For peer review only

Table3. Odds ratios and 95% confidence intervals for components of metabolic syndrome according to eating rate (n = 56,865)

	Men (n = 41,820)				Women (n = 15,045)			
Eating rate	Slow	Normal [a]	Fast	P for trend [b]	Slow	Normal [a]	Fast	P for trend [b]
n (%)	2,821 (6.8)	24,893 (59.5)	14,106 (33.7)		1,398 (9.3)	9,893 (65.8)	3,754 (24.9)	
Central obesity [c]								
Model 1 [d]	0.63 (0.56 to 0.71)	1.00	1.98 (1.89 to 2.08)	<0.001	0.73 (0.64 to 0.83)	1.00	1.44 (1.34 to 1.56)	<0.001
Model 2 [e]	0.63 (0.56 to 0.70)	1.00	1.97 (1.88 to 2.07)	<0.001	0.73 (0.64 to 0.83)	1.00	1.44 (1.33 to 1.56)	<0.001

For peer review only - http://bmjopen.bmj.com/site/about/guidelines.xhtml

For peer review only

High blood pressure [f]								
Model 1 [d]	0.75 (0.69 to 0.82)	1.00	1.22 (1.17 to 1.27)	<0.001	0.76 (0.66 to 0.88)	1.00	1.10 (1.01 to 1.21)	<0.001
Model 2 [e]	0.74 (0.68 to 0.81)	1.00	1.20 (1.15 to 1.26)	<0.001	0.76 (0.65 to 0.88)	1.00	1.10 (1.00 to 1.20)	<0.001
Model 3 [g]	0.88 (0.81 to 0.96)	1.00	0.97 (0.93 to 1.02)	0.645	0.85 (0.72 to 0.99)	1.00	0.93 (0.84 to 1.02)	0.923
High fasting plasma glucose [h]								
Model 1 [d]	0.78 (0.71 to 0.87)	1.00	1.17 (1.12 to 1.23)	<0.001	1.03 (0.85 to 1.25)	1.00	1.17 (1.04 to 1.31)	0.035
Model 2 [e]	0.78 (0.71 to	1.00	1.16 (1.11 to	<0.00	1.03 (0.85 to	1.00	1.16 (1.03 to	0.042

For peer review only

For peer review only - http://bmjopen.bmj.com/site/about/guidelines.xhtml

	0.87)		1.22)	1	1.25)		1.31)	
Model 3 [g]	0.88 (0.80 to 0.98)	1.00	0.99 (0.94 to 1.05)	0.238	1.14 (0.94 to 1.40)	1.00	1.02 (0.90 to 1.15)	0.536
High triglyceride [i]								
Model 1 [d]	0.88 (0.80 to 0.96)	1.00	1.32 (1.26 to 1.38)	<0.001	0.83 (0.67 to 1.01)	1.00	1.14 (1.01 to 1.28)	0.002
Model 2 [e]	0.90 (0.82 to 0.98)	1.00	1.33 (1.27 to 1.39)	<0.001	0.81 (0.66 to 1.00)	1.00	1.13 (1.01 to 1.27)	0.002
Model 3 [g]	1.08 (0.98 to 1.19)	1.00	1.07 (1.02 to 1.12)	0.121	0.90 (0.73 to 1.11)	1.00	0.98 (0.87 to 1.11)	0.753
Low HDL-C [j]								

For peer review only

Model 1 [d]	0.83 (0.73 to 0.96)	1.00	1.34 (1.26 to 1.43)	<0.001	0.90 (0.74 to 1.09)	1.00	1.11 (0.99 to 1.25)	0.018
Model 2 [e]	0.83 (0.73 to 0.96)	1.00	1.36 (1.28 to 1.45)	<0.001	0.89 (0.74 to 1.08)	1.00	1.12 (1.00 to 1.26)	0.011
Model 3 [g]	0.97 (0.84 to 1.12)	1.00	1.10 (1.03 to 1.18)	0.004	1.00 (0.82 to 1.22)	1.00	0.96 (0.85 to 1.08)	0.500

BMI=body mass index; HDL-C=high to density lipoprotein cholesterol.

[a] Reference.

[b] Multiple logistic regression, assigning ordinal number (0 to 2) to eating rate.

[c] Waist circumference ≥90 cm in men and ≥80 cm in women.

[d] Adjusted for age.

[e] Adjusted for age, smoking status, alcohol and regular physical activity.

For peer review only

For peer review only - http://bmjopen.bmj.com/site/about/guidelines.xhtml

[f] Blood pressure ≥130 mm Hg for systolic or ≥85 mm Hg for diastolic.

[g] Adjusted for age, smoking status, alcohol, regular physical activity and body mass index.

[h] Fasting plasma glucose ≥100 mg/dL or under medication.

[i] Triglyceride ≥150 mg/dL or under medication.

[j] HDL-C <40 mg/dL in men, <50 mg/dL in women or under medication.

For peer review only

For peer review only - http://bmjopen.bmj.com/site/about/guidelines.xhtml

Acknowledgments

The authors would like to thank Dr. Nobuo Yanagisawa and Dr. Takeshi Kawaguchi for their coordinating the study.

Contributors: SN and KKurotani designed study and drafted the manuscript. SN, NMP, AN, KKuwahara performed the data analysis. MD collected and interpreted the data. All authors have participated in the interpretation of the findings, revised it critically for important intellectual content and approved final version to be published. TM and YN provided administrative, technical and material support.SN and TM are guarantors.

Funding: This study was supported by the Industrial Health Foundation.

Competing interests: All authors have completed the ICMJE uniform disclosure form at www.icmje.org/coi_disclosure.pdf and declare: all authors declare no interests.

Ethical approval: The research protocol was approved by the Ethics Committee of the National Center for Global Health and Medicine and the Ethics Committee of Toho University.

Data sharing: No additional data available.

Transparency: SN affirms that the manuscript is an honest, accurate, and transparent account of the study being reported; that no important aspects of the study have been omitted; and that any discrepancies from the study as planned (and, if relevant,

For peer review only

registered) have been explained.

For peer review only

For peer review only - http://bmjopen.bmj.com/site/about/guidelines.xhtml

References

1. Eckel RH, Alberti KG, Grundy SM, et al. The metabolic syndrome. *Lancet* 2010;**375**:181-83.

2. Gomez-Martinez S, Martinez-Gomez D, Perez de Heredia F, et al. Eating habits and total and abdominal fat in Spanish adolescents: influence of physical activity. The AVENA study. *J Adolesc Health* 2012;**50**:403-09.

3. Ilanne-Parikka P, Eriksson JG, Lindstrom J, et al. Effect of lifestyle intervention on the occurrence of metabolic syndrome and its components in the Finnish Diabetes Prevention Study. *Diabetes care* 2008;**31**:805-07.

4. Nanri A, Tomita K, Matsushita Y, et al. Effect of six months lifestyle intervention in Japanese men with metabolic syndrome: randomized controlled trial. *J Occup Health* 2012;**54**:215-22.

5. Ferster CB, Nurnberger JI, Levitt EB. The control of eating. 1962. *Obes Res* 1996;**4**:401-10.

6. Hill SW, McCutcheon NB. Eating responses of obese and nonobese humans during dinner meals. *Psychosom Med* 1975;**37**:395-401.

7. Bellack AS. Behavior therapy for weight reduction. *Addict Behav* 1975;**1**:73-82.

8. Otsuka R, Tamakoshi K, Yatsuya H, et al. Eating fast leads to obesity: findings based

For peer review only - http://bmjopen.bmj.com/site/about/guidelines.xhtml

on self-administered questionnaires among middle-aged Japanese men and women. *J Epidemiol* 2006;**16**:117-24.

9. Sasaki S, Katagiri A, Tsuji T, et al. Self-reported rate of eating correlates with body mass index in 18-y-old Japanese women. *Int J Obes Relat Metab Disord* 2003;**27**:1405-10.

10. Maruyama K, Sato S, Ohira T, et al. The joint impact on being overweight of self reported behaviours of eating quickly and eating until full: cross sectional survey. *BMJ* 2008;**337**:a2002.

11. Lee KS, Kim DH, Jang JS, et al. Eating rate is associated with cardiometabolic risk factors in Korean adults. *Nut Metab Cardiovasc Dis* 2013;**23**:635-41.

12. Otsuka R, Tamakoshi K, Yatsuya H, et al. Eating fast leads to insulin resistance: findings in middle-aged Japanese men and women. *Prev Med* 2008;**46**:154-59.

13. Radzeviciene L, Ostrauskas R. Fast eating and the risk of type 2 diabetes mellitus: a case-control study. *Clin Nutr* 2013;**32**:232-35.

14. Ohkuma T, Fujii H, Iwase M, et al. Impact of eating rate on obesity and cardiovascular risk factors according to glucose tolerance status: the Fukuoka Diabetes Registry and the Hisayama Study. *Diabetologia* 2013;**56**:70-77.

15. Shigeta H, Shigeta M, Nakazawa A, et al. Lifestyle, obesity, and insulin resistance.

For peer review only - http://bmjopen.bmj.com/site/about/guidelines.xhtml

Diabetes care 2001;**24**:608.

16. Gerace TA, George VA. Predictors of weight increases over 7 years in fire fighters and paramedics. *Prev Med* 1996;**25**:593-600.

17. Tanihara S, Imatoh T, Miyazaki M, et al. Retrospective longitudinal study on the relationship between 8-year weight change and current eating speed. *Appetite* 2011;**57**:179-83.

18. Sakurai M, Nakamura K, Miura K, et al. Self-reported speed of eating and 7-year risk of type 2 diabetes mellitus in middle-aged Japanese men. *Metabolism* 2012;**61**:1566-71.

19. Kral JG, Buckley MC, Kissileff HR, et al. Metabolic correlates of eating behavior in severe obesity. *Int J Obes Relat Metab Disord* 2001;**25**:258-64.

20. Shin A, Lim SY, Sung J, et al. Dietary intake, eating habits, and metabolic syndrome in Korean men. *J Am Diet Assoc* 2009;**109**:633-40.

21. Shen W, Punyanitya M, Chen J, et al. Waist circumference correlates with metabolic syndrome indicators better than percentage fat. *Obesity (Silver Spring)* 2006;**14**:727-36.

22. Alberti KG, Eckel RH, Grundy SM, et al. Harmonizing the metabolic syndrome: a joint interim statement of the International Diabetes Federation Task Force on Epidemiology and Prevention; National Heart, Lung, and Blood Institute; American

For peer review only

For peer review only - http://bmjopen.bmj.com/site/about/guidelines.xhtml

Heart Association; World Heart Federation; International Atherosclerosis Society; and International Association for the Study of Obesity. *Circulation* 2009;**120**:1640-5.

23. Ministry of Justice, Japan. Industrial Safety and Health Act, Article 66 (Medical Examination). Available at: http://www.japaneselawtranslation.go.jp/law/detail/?id=1926&vm=&re=. Accessed 28 July 2014.

24. Ministry of Health, Labour and Welfare. Specific Health Checkups and Specific Health Guidance. Secondary Specific Health Checkups and Specific Health Guidance. Available at: http://www.mhlw.go.jp/english/wp/wp-hw3/dl/2-007.pdf. Accessed 28 July 2014.25. The Ministry of Health, Labour and Welfare, Ministry of Education, Culture, Sports, Science and Technology. Ethical guidelines for epidemiological research. 2002. Available at: http://www.niph.go.jp/wadai/ekigakurinri/guidelines.pdf. Accessed 28 July, 2014.

26. Ministry of Health, Labour and Welfare. Questionnaire of Specific Health Checkups. Secondary Questionnaire of Specific Health Checkups. Available at: http://www.mhlw.go.jp/seisakunitsuite/bunya/kenkou_iryou/kenkou/seikatsu/dl/hoken-program2_02.pdf. Accessed 28 July 2014. (in Japanese)

27. Hill SW, McCutcheon NB. Contributions of obesity, gender, hunger, food preference,

For peer review only - http://bmjopen.bmj.com/site/about/guidelines.xhtml

and body size to bite size, bite speed, and rate of eating. *Appetite* 1984;**5**:73-83.

28. Ekuni D, Furuta M, Takeuchi N, et al. Self-reports of eating quickly are related to a decreased number of chews until first swallow, total number of chews, and total duration of chewing in young people. *Arch Oral Biol* 2012;**57**:981-86.

29. Rolls ET. Sensory processing in the brain related to the control of food intake. *Proc Nutr Soc* 2007;**66**:96-112.

30. Morton GJ, Cummings DE, Baskin DG, et al. Central nervous system control of food intake and body weight. *Nature* 2006;**443**:289-95.

31. de Graaf C, Blom WA, Smeets PA, et al. Biomarkers of satiation and satiety. *Am J Clin Nutr* 2004;**79**:946-61.

32. Kokkinos A, le Roux CW, Alexiadou K, et al. Eating slowly increases the postprandial response of the anorexigenic gut hormones, peptide YY and glucagon-like peptide-1. *J Clin Endocrinol Metab* 2010;**95**:333-37.

33. Sobki SH, Zaid AA, Khan HA, et al. Significant impact of pace of eating on serum ghrelin and glucose levels. *Clin Biochem* 2010;**43**:522-24.

34. Viskaal-van Dongen M, Kok FJ, de Graaf C. Eating rate of commonly consumed foods promotes food and energy intake. *Appetite* 2011;**56**:25-31.

35. Scisco JL, Muth ER, Dong Y, et al. Slowing bite-rate reduces energy intake: an

For peer review only

For peer review only - http://bmjopen.bmj.com/site/about/guidelines.xhtml

application of the bite counter device. *J Am Diet Assoc* 2011;**111**:1231-35.

36. Mochizuki K, Misaki Y, Miyauchi R, et al. A higher rate of eating is associated with higher circulating interluekin-1beta concentrations in Japanese men not being treated for metabolic diseases. *Nutrition* 2012;**28**:978-83.

37. Besedovsky H, del Rey A, Sorkin E, et al. Immunoregulatory feedback between interleukin-1 and glucocorticoid hormones. *Science* 1986;**233**:652-54

38. Matsuki T, Horai R, Sudo K, et al. IL-1 plays an important role in lipid metabolism by regulating insulin levels under physiological conditions. *J Exp Med* 2003;**198**:877-88.

39. Yatabe MS, Yatabe J, Yoneda M, et al. Salt sensitivity is associated with insulin resistance, sympathetic overactivity, and decreased suppression of circulating renin activity in lean patients with essential hypertension. *Am J Clin Nutr* 2010;**92**:77-82.

40. Facchini FS, DoNascimento C, Reaven GM, et al. Blood pressure, sodium intake, insulin resistance, and urinary nitrate excretion. *Hypertension* 1999;**33**:1008-12.

41. Pollare T, Lithell H, Berne C. Insulin resistance is a characteristic feature of primary hypertension independent of obesity. *Metabolism*. 1990;**39**:167-74.

42. De Vogli R, Kouvonen A, Gimeno D. The influence of market deregulation on fast food consumption and body mass index: a cross national time series analysis. *Bull*

For peer review only - http://bmjopen.bmj.com/site/about/guidelines.xhtml

World Health Organ 2014;**92**:99-107A.

43. Ministry of Health, Labour and Welfare. Special Survey on Industrial Safety and Health. Available at: http://www.mhlw.go.jp/bunya/roudoukijun/anzeneisei11/h23.html. Accessed 28 July 2014. (in Japanese)

For peer review only

For peer review only - http://bmjopen.bmj.com/site/about/guidelines.xhtml

Self-reported eating rate and metabolic syndrome in Japanese: cross-sectional study

Satsue Nagahama [1 2 3*], Kayo Kurotani [1], Ngoc Minh Pham [4], Akiko Nanri [1], Keisuke Kuwahara [1], Masashi Dan [2], Yuji Nishiwaki [3], Tetsuya Mizoue [1]

[1] Department of Epidemiology and Prevention, Center for Clinical Sciences, National Center for Global Health and Medicine, 1-21-1, Toyama, Shinjuku-ku, Tokyo, Japan, Zip162-8655; Tel +81-3-3202-7181, Fax 81-3-3202-7364,

E-mail: satsue_0323@hotmail.com

[2] All Japan Labor Welfare Foundation, Tokyo, Japan;

[3] Department of Environmental and Occupational Health, School of Medicine, Toho University, Tokyo, Japan;

[4] Department of Epidemiology, Faculty of Public Health, Thai Nguyen University of Medicine and Pharmacy, Thai Nguyen Province, Vietnam;

Keywords: eating rate, metabolic syndrome, health checkup, Japan, Joint Interim Statement

For peer review only - http://bmjopen.bmj.com/site/about/guidelines.xhtml

Word count: 2875

For peer review only

For peer review only - http://bmjopen.bmj.com/site/about/guidelines.xhtml

ABSTRACT

Objectives: To examine the association between self-reported eating rate and metabolic syndrome.

Design: Cross-sectional study.

Setting: Annual health checkup at a health check service center in Japan.

Participants: A total of 56,865 participants (41,820 males and 15,045 females) who attended health checkup in 2011 and reported not to have a history of coronary heart disease or stroke.

Main outcome measure: Metabolic syndrome was defined by the joint of interim statement of the International Diabetes Federation and the American Heart Association/National Heart, Lung, and Blood Institute.

Results: In multiple logistic regression models, eating rate was significantly and positively associated with metabolic syndrome. The multivariable-adjusted odds ratios (95% confidence interval) for slow, normal and fast were 0.70 (0.62 to 0.79), 1.00 (reference) and 1.61 (1.53 to 1.70), respectively, in men (P for trend <0.001), and 0.74 (0.60 to 0.91), 1.00 (reference) and 1.27 (1.13 to 1.43), respectively, in women (P for trend <0.001). Of metabolic syndrome components, abdominal obesity showed the strongest association with eating rate. The associations of eating rate and metabolic

For peer review only - http://bmjopen.bmj.com/site/about/guidelines.xhtml

syndrome and its components were largely attenuated after further adjustment for body mass index; however, the association of slow eating with lower odds of high blood pressure (men and women) and hyperglycemia (men) and that of fast eating with higher odds of lipid abnormality (men) remained statistically significant.

Conclusions: Results suggest that eating rate is associated with the presence of metabolic syndrome and that this association is largely accounted for by the difference of body mass according to eating rate.

For peer review only

Strength and Limitation

This study included a large number of participants, used waist circumference in defining metabolic syndrome, and analyzed data for men and women separately.

Eating rate was assessed by a self-reported questionnaire. Information on dietary intake was not obtained.

For peer review only

For peer review only - http://bmjopen.bmj.com/site/about/guidelines.xhtml

Introduction

Metabolic syndrome (MetS) is a cluster of physiological risk factors associated with cardiovascular disease and several types of cancer.[1] Determination of etiologic factors for MetS is required for the establishment of public health strategies to reduce its prevalence and prevent resulting complications. Growing evidence from both observational and interventional studies suggests a role of dietary habits in the development of MetS,[2-4] which originates from obesity. Obesity has been extensively investigated in relation to dietary habits including eating rate since 1962, when Ferster published a theoretical and practical weight control program focusing on eating behaviors including eating rate.[5] Observational studies showed that obese people ate at a faster rate than non-obese people,[6] and reducing eating rate may be a simple and effective therapy for obesity.[7]

During the past decade, several cross-sectional studies have found a positive association between eating rate and overweight[8-11] or insulin resistance.[11-15] Similarly, a few longitudinal studies showed that eating fast was associated with an increased risk of weight gain[16, 17] and type 2 diabetes.[18] In addition, some cross-sectional studies have reported that fast eating was positively associated with hypertriglyceridemia and low high-density lipoprotein cholesterol (HDL-C).[11, 14, 19] Therefore it is conceivable that

For peer review only - http://bmjopen.bmj.com/site/about/guidelines.xhtml

eating rate may be associated with MetS. To our knowledge, however, only one Korean study cross-sectionally examined eating rate in relation to MetS.[20] In that study, MetS was defined by using body mass index (BMI), rather than waist circumference, and investigated the association in men only. Waist circumference is a component of most MetS definitions as a surrogate of central obesity, which can better predict cardiovascular risk.[21] It is therefore necessary to examine the relationship between eating rate and MetS using waist circumference in both men and women. Here, we investigated cross-sectionally the association between self-reported eating rate and the presence of MetS according to the joint of interim statement of the International Diabetes Federation and the American Heart Association/National Heart, Lung, and Blood Institute (JIS)[22] using a large dataset of health checkup in Japanese men and women.

METHODS AND PROCEDURES

Study population

In Japan, health checkup under occupational health and safety law is mandatory for all employed workers[23] and has been modified in 2008 when the recommendation for new national health checkup system focusing on MetS has been launched.[24] Study

For peer review only - http://bmjopen.bmj.com/site/about/guidelines.xhtml

participants were attendants of 2011 (calendar year) annual health checkup at All Japan Labor Welfare Foundation (Tokyo), a health service center performing health checkup. Participants were mainly Japanese employees but also included a small number of their dependents and foreign workers, aged 17-99 in men and 17-85 in women. Of 297,148 participants, we excluded 3,660 with a history of myocardial infarction, coronary heart disease or stroke, which might influence both eating rate and MetS. Of the remaining 293,488 participants, we included 269,297 who reported their eating rate. Of these, we excluded 182,487 with missing data for any of the components of MetS (173,376 without plasma glucose, 61,602 without waist circumference, 43,724 without triglyceride, 43,401 without HDL-C and 504 without blood pressure; some participants had two or more missing data). Major reason for a large number of participants with missing measurement of blood glucose was that HbA1c was measured instead of blood glucose for those who attended checkup in non-fasting condition. Of the remaining 86,810 participants, we further excluded 29,337 who took meal within 8 hours before blood drawing or provided no information on meal time. After further exclusion of 608 participants with missing information on covariates (BMI, smoking status, alcohol consumption and physical activity), 56,865 participants (41,820 males and 15,045 females) remained for analysis. We did not obtain written informed consent from each

For peer review only - http://bmjopen.bmj.com/site/about/guidelines.xhtml

participant; instead, we disclosed the execution of the study by showing posters, giving participants an opportunity to refuse the use of their data for the study. In Japan, informed consent is not necessarily required for observational studies using existing data, as described in the Japanese Ethical Guidelines for Epidemiological Research.[25] The research protocol was approved by the Ethics Committee of the National Center for Global Health and Medicine and the Ethics Committee of Toho University.

Data collection and measurements

A self-administered questionnaire, which was recommended for specific health examination by the Japanese government (Ministry of Health, Labour and Welfare),[26] was used to assess eating rate, medical history and health-related lifestyles including smoking, alcohol consumption and regular physical activity. Eating rate was assessed by asking “How fast is your speed of eating?”, with three response options (slow, normal and fast). A trained staff measured height to the nearest 0.1 cm, weight to the nearest 0.1 kg and waist circumference to the nearest 0.1 cm at the umbilical level in a standing position. BMI was calculated as the weight in kilograms divided by the squared height in meters. Blood pressure in the sitting position was measured using an automated machine (HEM-907, Omron, Kyoto, Japan). Participants with high blood pressure (≥130 mm Hg systolic or ≥85 mm Hg diastolic) received another measurement and data

For peer review only - http://bmjopen.bmj.com/site/about/guidelines.xhtml

showing lower systolic blood pressure was used. Venous blood sample was collected, stored in a cooler at 4 degrees for transportation to an external laboratory (SRL, Tokyo, Japan) and measured within 24 hours of blood drawing. Triglyceride level was measured by enzymatic colorimertic test (Bio Majesty JCA-BM8060, JEOL, Tokyo, Japan). HDL-C level was determined by a direct method (Bio Majesty JCA-BM8060, JEOL, Tokyo, Japan). Plasma glucose level was determined using by the hexokinase method (an automatic clinical chemistry analyzer JCA-BM9000 series, JEOL, Tokyo, Japan).

Definitions for MetS

According to the JIS criteria, MetS was defined as three or more of the following risk factors: 1) waist circumference for Asian population ≥90 cm in men and ≥80 cm in women, 2) triglyceride level ≥150 mg/dL (1.7 mmol/L), 3) HDL-C level <40 mg/dL (1.04 mmol/L) in men and <50 mg/dL (1.3 mmol/L) in women, 4) blood pressure ≥130 mm Hg systolic or ≥85 mm Hg diastolic, 5) fasting glucose level ≥100 mg/dL (5.6 mmol/L).[22] Participants under medication for diabetes, hypertension and dyslipidemia were considered as having respective factor, irrespective of measured data.

Statistical analysis

Participants were divided into three groups according to eating rate (slow,

For peer review only - http://bmjopen.bmj.com/site/about/guidelines.xhtml

normal and fast). The characteristics of participants across eating rate categories were expressed as means (standard deviation) for continuous variables and percentages for categorical variables, respectively. Fasting plasma glucose and triglyceride were highly skewed; hence, they were log-transformed and expressed as geometric means (95% confidence intervals (CI)). Trend association was assessed by assigning ordinal numbers (0 to 2) to the categories of eating rate (slow, normal and fast, respectively) and was tested using linear regression and logistic regression, as appropriate. Multiple logistic regression analysis was used to estimate the odds ratios (OR) with 95% CI for the presence of MetS across eating rate categories, with normal eating rate as the reference. We adjusted for age (continuous, year) in the basic model. In the second model, we further adjusted for smoking status (non-smoker, daily smoker consuming <20 cigarettes per day or ≥20 cigarettes per day), physical activity (walking time <60 min per day or ≥60 min per day), and alcohol consumption (non-drinker, <1 *go*, 1 to <2 *go* or ≥2 *go* per day; one *go* of sake, Japanese traditional beverage, is about 180 ml of 10 to 14% of ethanol and contains ~23g of ethanol). In the third model, we added BMI (continuous, kg/m^2) to the second model. We performed likelihood ratio test for testing the interaction between eating rate and sex. All analyses were done for men and women separately because the interaction was significant (P for interaction <0.001). We

For peer review only - http://bmjopen.bmj.com/site/about/guidelines.xhtml

repeated the above analyses for each component of MetS. Two-tailed P value <0.05 was considered statistically significant. All statistical analyses were performed with STATA, version 12.1 (StataCorp, College Station, TX, USA).

RESULTS

The prevalence of MetS was 18.5% in men and 12.8 % in women. **Table 1** shows characteristics of the study participants across categories of eating rate. Men who ate fast tended to be young, whereas women who ate slowly tended to be young. Both men and women who ate fast consumed greater amount of alcohol and had significantly higher BMI, waist circumference, triglyceride level and systolic and diastolic blood pressures and lower HDL-C level than those who ate slowly.

The ORs of the presence of MetS across eating rate are shown in **Table 2**. Faster eating was associated with higher presence of MetS in age- and multivariable-adjusted models. The trend was more apparent in men than in women. The multivariable-adjusted ORs (95% CI) of MetS for eating slow, normal and fast rate were 0.70 (0.62 to 0.79), 1.00 (reference) and 1.61 (1.53 to 1.70), respectively, in men (P for trend <0.001), and 0.74 (0.60 to 0.91), 1.00 (reference) and 1.27 (1.13 to 1.43), respectively, in women (P for trend <0.001). Further adjustment for BMI markedly

For peer review only - http://bmjopen.bmj.com/site/about/guidelines.xhtml

attenuated these associations; however, the association with fast eating and MetS remained statistically significant in men.

Table 3 shows the ORs of the presence of individual MetS components across three categories of eating rate. Central obesity sharply increased with increasing speed of eating; the ORs for slow, normal and fast eating were 0.63, 1.00 (reference) and 1.97, respectively, in men (P for trend <0.001), and 0.73, 1.00(reference) and 1.44, respectively, in women (P for trend <0.001). High blood pressure and high triglyceride were positively associated with eating rate in both men and women. High fasting plasma glucose and low HDL-C were associated with fast eating in both men and women, but they were associated with slow eating in men only. Additional adjustment for BMI largely attenuated these associations and the significant trend association disappeared, but the associations of slow eating with decreased odds of high blood pressure (men and women) and hyperglycemia (men) and those of fast eating with increased odds of abnormal lipid profile (men) remained statistically significant.

DISCUSSION

In this large population of Japanese men and women, we found that eating rate was positively associated with the presence of MetS, especially in men. Of components

For peer review only - http://bmjopen.bmj.com/site/about/guidelines.xhtml

of MetS, the association with abdominal obesity was strongest. These associations were largely attenuated after adjustment for BMI. However, slow eating was associated with decreased odds of high blood pressure in both men and women and high fasting plasma glucose in men, and fast eating was associated with increased odds of lipid abnormal profiles in men. To our best knowledge, the present study is the first to report a positive association between eating rate and MetS defined by using waist circumference.

The present finding for MetS is consistent with that of a study among Korean men reporting that eating rate was positively associated with MetS, which was defined by using BMI instead of waist circumference.[20] As regards MetS components, our study is compatible with some cross-sectional studies showing that eating rate was associated with higher BMI[8-11] and two longitudinal studies showing that eating rate was associated with weight gain.[16, 17] In a Korean study that elucidated the association between eating rate and components of MetS for men and women separately, eating rate was associated with obesity, high blood pressure, hyperglycemia and abnormal lipid profile in men, whereas it was associated with only obesity in women.[11] Our results were largely consistent with those in the Korean study (except for blood pressure in women).

For peer review only - http://bmjopen.bmj.com/site/about/guidelines.xhtml

Notably, we found that the associations of MetS components with eating rate were largely attenuated after adjustment for BMI, a finding compatible with those of a cross-sectional study in Korea[11] and a prospective study in Japan.[18] This result indicates that obesity is a mediator whereby fast eating deteriorates MetS components. We also found, however, that some associations remained statistically significant even after adjusting for BMI (dyslipidemia with fast eating and hyperglycemia with slow eating in men, and high blood pressure with slow eating in both men and women). Similarly, the above-mentioned Korean study[11] reported that high rate of eating remained an important determinant for low HDL-C and high fasting plasma glucose after adjustment for BMI in men. Therefore, there may be pathways other than weight gain that might underlie the association between eating rate and MetS.

We found that the association between eating rate and MetS was stronger in men than in women, consistent with a previous study in Korea.[11] Such sex difference may reflect the difference in actual eating speed between men and women. One study elucidated that women took more bites, smaller bite size and slower bites than men in eating the same amount of doughnut, irrespectively of body size.[27] Another study showed that objectively measured eating speed in men with self-reported slow eating

For peer review only - http://bmjopen.bmj.com/site/about/guidelines.xhtml

was faster than that in women with self-reported fast eating.[28] Taken together, eating rate may have a greater impact on metabolism in men than that in women.

Although mechanisms whereby eating rate influences metabolism is not fully elucidated, overeating may link fast eating to MetS. Fast eating gives few satiety signal from oral cavity to the brain,[29, 30] induces less satiation and satiety due to a lack of stomach expansion[31] and alters the circulating levels of certain gut hormones.[32, 33] In these pathways, fast eating leads to excess energy intake,[34, 35] resulting in overweight and MetS. Because fast eating has been associated with obesity even after adjusting for total energy intake,[8-11, 14] there may be other pathways. One study showed that interleukin-1β and interleukin-6 were higher among those who ate fast than among those who ate slowly, even after accounting for energy intake and BMI.[36] These cytokines could induce insulin resistance,[37, 38] contributing to high blood pressure via an increased renal sodium and water retention, plasma noradrenalin and sympathetic nervous system activity.[39-41]

The strengths of our study deserve mention. The present study has large sample size (56,865 participants). In addition, body weight, body height and waist circumference were measured by trained technicians, which increased the validity of our study. Nonetheless, several limitations in the present study merit consideration. First,

For peer review only - http://bmjopen.bmj.com/site/about/guidelines.xhtml

eating rate was self-reported. However, self-reported eating rate has been shown to be well correlated with friend-reported one[9] or objectively measured one.[28] Second, information on dietary intake was not available in the present study and thus total energy intake was not considered in analyses. The adjustment of energy intake, however, may not be appropriate because energy intake may increase with eating rate and thus may act as a mediator rather than confounder. Moreover, eating rate has been associated with body weight independent of energy intake.[7-10,13] Third, fast-food is an energy-dense dietary source and has been linked to MetS.[42] Because fast-food is usually consumed quickly, it may confound the association of eating rate with MetS. Fourth, the study participants were mainly workers in various industries including manufacturing (43.6%), service (27.8%) and transport and telecommunications (9.9%), and these figures are similar to those of national survey.[43] However, information on profession of participants was not available, and thus caution is required when generalize the present finding. Fifth, a large number of participants were excluded from the present analysis due to missing data for MetS components. We cannot deny a possibility of bias due to such selective inclusion. Sixth, cross-sectional design precludes any causal inferences about the role of eating rate. Finally, we cannot exclude a possibility of the effects of residual confounding and confounding by unmeasured variables.

For peer review only - http://bmjopen.bmj.com/site/about/guidelines.xhtml

For peer review only

In conclusion, we found a positive trend association between self-reported eating rate and the presence of MetS in men and women. The association between eating rate and MetS was largely accounted for by the difference of body mass across eating rate. Further research should address whether reducing eating rate prevents obesity and MetS.

For peer review only

For peer review only - http://bmjopen.bmj.com/site/about/guidelines.xhtml

Table1. Characteristics of the study individuals according to eating rates

	Men (n = 41,820)			P for trend [a]	Women (n = 15,045)			P for trend [a]
	Slow	Normal	Fast		Slow	Normal	Fast	
n (%)	2,821 (6.8)	24,893 (59.5)	14,106 (33.7)		1,398 (9.3)	9,893 (65.8)	3,754 (24.9)	
Age (years) [b]	46.9 ± 12.3	46.9 ± 10.9	45.0 ± 10.4	<0.001	43.5 ± 12.5	47.2 ± 11.6	46.7 ± 11.2	<0.001
Walking time, ≥60 min/day (%)	21.8	19.0	20.6	0.004	15.5	15.0	16.1	0.798
Smoking status (%)								
Non-smoker	61.9	55.0	56.6	<0.001	82.9	83.1	80.7	0.572
Daily consuming <20 cigarettes /day	28.6	34.6	31.3		16.0	15.7	17.6	
Daily consuming ≥20 cigarettes /day	9.5	10.4	12.1		1.1	1.2	1.7	

For peer review only - http://bmjopen.bmj.com/site/about/guidelines.xhtml

Alcohol (%)								
Non-drinker	30.2	26.7	26.7	<0.001	53.4	52.2	49.8	<0.001
Drinker <1 *go* /day [d]	33.9	35.7	34.5		34.2	35.9	35.5	
Drinker 1 to <2 *go* /day [d]	24.6	26.3	26.8		9.5	9.5	11.5	
Drinker ≥2 *go* /day [d]	11.3	11.3	12.0		2.9	2.4	3.2	
BMI (kg/m^2)[b]	22.4 ± 3.3	23.4 ± 3.3	24.6 ± 3.7	<0.001	21.0 ± 3.5	21.8 ± 3.5	22.5 ± 3.8	<0.001
Waist circumference (cm)[b]	80.3 ± 9.2	82.9 ± 9.0	86.0 ± 9.8	<0.001	75.5 ± 9.5	77.7 ± 9.4	79.6 ± 9.8	<0.001
Systolic blood pressure (mm Hg)[b]	123.5 ± 15.5	126.1 ± 15.5	126.7 ± 15.1	<0.001	113.1 ± 16.3	117.3 ± 17.2	117.0 ± 17.2	<0.001
Diastolic blood pressure (mm Hg)[b]	75.2 ± 11.4	77.3 ± 11.9	78.0 ± 12.0	<0.001	69.1 ± 10.9	71.4 ± 11.5	71.5 ± 11.9	<0.001
Fasting plasma glucose (mg/dL)[c]	93.0 (92.5 to 93.6)	94.4 (94.2 to 94.6)	94.6 (94.3 to 94.8)	<0.001	88.1 (87.5 to 88.7)	89.1 (88.9 to 89.3)	89.5 (89.1 to 89.9)	<0.001

For peer review only - http://bmjopen.bmj.com/site/about/guidelines.xhtml

Triglyceride (mg/dL)[c]	98.3 (96.2 to 100.4)	103.8 (103.0 to 104.6)	110.8 (109.8 to 111.9)	<0.001	67.0 (65.3 to 68.7)	71.6 (70.9 to 72.3)	74.1 (72.9 to 75.2)	<0.001
HDL-C (mg/dL)[b]	61.3 ± 15.3	59.4 ± 15.0	57.2 ± 14.3	<0.001	71.4 ± 15.3	70.5 ± 15.8	69.3 ± 15.5	<0.001

Cross to sectional survey of 56,865 examinees in All Japan Labor Welfare Foundation, Japan, 2011.

BMI=body mass index; HDL-C=high to density lipoprotein cholesterol.

[a] Linear regression and logistic regression, assigning ordinal number (0 to 2) to eating rate, as appropriate.

[b] Mean ± SD.

[c] Geometric means (95% confidence intervals).

[d] One *go* contains ~25g of ethanol.

For peer review only - http://bmjopen.bmj.com/site/about/guidelines.xhtml

Table2. Odds ratios and 95% confidence intervals for metabolic syndrome according to eating rate (n = 56,865)

	Men (n = 41,820)				Women (n = 15,045)			
Eating rate	Slow	Normal [a]	Fast	P for trend [b]	Slow	Normal [a]	Fast	P for trend [b]
n (%)	2,821 (6.8)	24,893 (59.5)	14,106 (33.7)		1,398 (9.3)	9,893 (65.8)	3,754 (24.9)	
MetS, n	361	4,180	3,193		116	1,261	547	
Model 1 [c]	0.70 (0.62 to 0.79)	1.00	1.62 (1.53 to 1.71)	<0.001	0.75 (0.61 to 0.92)	1.00	1.27 (1.13 to 1.42)	<0.001
Model 2 [d]	0.70 (0.62 to 0.79)	1.00	1.61 (1.53 to 1.70)	<0.001	0.74 (0.60 to 0.91)	1.00	1.27 (1.13 to 1.43)	<0.001
Model 3 [e]	0.91 (0.80 to 1.04)	1.00	1.10 (1.03 to 1.17)	<0.001	0.88 (0.70 to 1.11)	1.00	0.98 (0.86 to 1.12)	0.714

MetS=Metabolic syndrome.

For peer review only - http://bmjopen.bmj.com/site/about/guidelines.xhtml

MetS as defined using the criteria of the Joint Interim Statement : the presence of three or more of the following risk factors: 1) waist circumference ≥90 cm in men and ≥80 cm in women, 2) triglyceride level ≥150 mg/dL (1.7 mmol/L), 3) HDL-C level <40 mg/dL (1.04 mmol/L) in men and <50 mg/dL (1.3 mmol/L) in women, 4) blood pressure ≥130 mm Hg systolic or ≥85 mm Hg diastolic, 5) fasting glucose level ≥100 mg/dL (5.6 mmol/L).

[a] Reference.

[b] Multiple logistic regression, assigning ordinal number (0 to 2) to eating rate.

[c] Adjusted for age.

[d] Adjusted for age, smoking status, alcohol and regular physical activity.

[e] Adjusted for age, smoking status, alcohol, regular physical activity and body mass index.

For peer review only - http://bmjopen.bmj.com/site/about/guidelines.xhtml

Table3. Odds ratios and 95% confidence intervals for components of metabolic syndrome according to eating rate (n = 56,865)

	Men (n = 41,820)				Women (n = 15,045)			
Eating rate	Slow	Normal [a]	Fast	P for trend [b]	Slow	Normal [a]	Fast	P for trend [b]
n (%)	2,821 (6.8)	24,893 (59.5)	14,106 (33.7)		1,398 (9.3)	9,893 (65.8)	3,754 (24.9)	
Central obesity [c]								
Model 1 [d]	0.63 (0.56 to 0.71)	1.00	1.98 (1.89 to 2.08)	<0.001	0.73 (0.64 to 0.83)	1.00	1.44 (1.34 to 1.56)	<0.001
Model 2 [e]	0.63 (0.56 to 0.70)	1.00	1.97 (1.88 to 2.07)	<0.001	0.73 (0.64 to 0.83)	1.00	1.44 (1.33 to 1.56)	<0.001

For peer review only - http://bmjopen.bmj.com/site/about/guidelines.xhtml

For peer review only

High blood pressure [f]								
Model 1 [d]	0.75 (0.69 to 0.82)	1.00	1.22 (1.17 to 1.27)	<0.001	0.76 (0.66 to 0.88)	1.00	1.10 (1.01 to 1.21)	<0.001
Model 2 [e]	0.74 (0.68 to 0.81)	1.00	1.20 (1.15 to 1.26)	<0.001	0.76 (0.65 to 0.88)	1.00	1.10 (1.00 to 1.20)	<0.001
Model 3 [g]	0.88 (0.81 to 0.96)	1.00	0.97 (0.93 to 1.02)	0.645	0.85 (0.72 to 0.99)	1.00	0.93 (0.84 to 1.02)	0.923
High fasting plasma glucose [h]								
Model 1 [d]	0.78 (0.71 to 0.87)	1.00	1.17 (1.12 to 1.23)	<0.001	1.03 (0.85 to 1.25)	1.00	1.17 (1.04 to 1.31)	0.035
Model 2 [e]	0.78 (0.71 to	1.00	1.16 (1.11 to	<0.00	1.03 (0.85 to	1.00	1.16 (1.03 to	0.042

For peer review only

For peer review only - http://bmjopen.bmj.com/site/about/guidelines.xhtml

	0.87)		1.22)	1	1.25)		1.31)	
Model 3 [g]	0.88 (0.80 to 0.98)	1.00	0.99 (0.94 to 1.05)	0.238	1.14 (0.94 to 1.40)	1.00	1.02 (0.90 to 1.15)	0.536
High triglyceride [i]								
Model 1 [d]	0.88 (0.80 to 0.96)	1.00	1.32 (1.26 to 1.38)	<0.001	0.83 (0.67 to 1.01)	1.00	1.14 (1.01 to 1.28)	0.002
Model 2 [e]	0.90 (0.82 to 0.98)	1.00	1.33 (1.27 to 1.39)	<0.001	0.81 (0.66 to 1.00)	1.00	1.13 (1.01 to 1.27)	0.002
Model 3 [g]	1.08 (0.98 to 1.19)	1.00	1.07 (1.02 to 1.12)	0.121	0.90 (0.73 to 1.11)	1.00	0.98 (0.87 to 1.11)	0.753
Low HDL-C [j]								

For peer review only

For peer review only - http://bmjopen.bmj.com/site/about/guidelines.xhtml

Model 1 [d]	0.83 (0.73 to 0.96)	1.00	1.34 (1.26 to 1.43)	<0.001	0.90 (0.74 to 1.09)	1.00	1.11 (0.99 to 1.25)	0.018
Model 2 [e]	0.83 (0.73 to 0.96)	1.00	1.36 (1.28 to 1.45)	<0.001	0.89 (0.74 to 1.08)	1.00	1.12 (1.00 to 1.26)	0.011
Model 3 [g]	0.97 (0.84 to 1.12)	1.00	1.10 (1.03 to 1.18)	0.004	1.00 (0.82 to 1.22)	1.00	0.96 (0.85 to 1.08)	0.500

BMI=body mass index; HDL-C=high to density lipoprotein cholesterol.

[a] Reference.

[b] Multiple logistic regression, assigning ordinal number (0 to 2) to eating rate.

[c] Waist circumference ≥90 cm in men and ≥80 cm in women.

[d] Adjusted for age.

[e] Adjusted for age, smoking status, alcohol and regular physical activity.

For peer review only

For peer review only - http://bmjopen.bmj.com/site/about/guidelines.xhtml

[f] Blood pressure $\geq$130 mm Hg for systolic or $\geq$85 mm Hg for diastolic.

[g] Adjusted for age, smoking status, alcohol, regular physical activity and body mass index.

[h] Fasting plasma glucose $\geq$100 mg/dL or under medication.

[i] Triglyceride $\geq$150 mg/dL or under medication.

[j] HDL-C <40 mg/dL in men, <50 mg/dL in women or under medication.

For peer review only

For peer review only - http://bmjopen.bmj.com/site/about/guidelines.xhtml

Acknowledgments

The authors would like to thank Dr. Nobuo Yanagisawa and Dr. Takeshi Kawaguchi for their coordinating the study.

Contributors: SN and KKurotani designed study and drafted the manuscript. SN, NMP, AN, KKuwahara performed the data analysis. MD collected and interpreted the data. All authors have participated in the interpretation of the findings, revised it critically for important intellectual content and approved final version to be published. TM and YN provided administrative, technical and material support.SN and TM are guarantors.

Funding: This study was supported by the Industrial Health Foundation.

Competing interests: All authors have completed the ICMJE uniform disclosure form at www.icmje.org/coi_disclosure.pdf and declare: all authors declare no interests.

Ethical approval: The research protocol was approved by the Ethics Committee of the National Center for Global Health and Medicine and the Ethics Committee of Toho University.

Data sharing: No additional data available.

Transparency: SN affirms that the manuscript is an honest, accurate, and transparent account of the study being reported; that no important aspects of the study have been omitted; and that any discrepancies from the study as planned (and, if relevant,

For peer review only

registered) have been explained.

For peer review only

For peer review only - http://bmjopen.bmj.com/site/about/guidelines.xhtml

References

1. Eckel RH, Alberti KG, Grundy SM, et al. The metabolic syndrome. *Lancet* 2010;**375**:181-83.

2. Gomez-Martinez S, Martinez-Gomez D, Perez de Heredia F, et al. Eating habits and total and abdominal fat in Spanish adolescents: influence of physical activity. The AVENA study. *J Adolesc Health* 2012;**50**:403-09.

3. Ilanne-Parikka P, Eriksson JG, Lindstrom J, et al. Effect of lifestyle intervention on the occurrence of metabolic syndrome and its components in the Finnish Diabetes Prevention Study. *Diabetes care* 2008;**31**:805-07.

4. Nanri A, Tomita K, Matsushita Y, et al. Effect of six months lifestyle intervention in Japanese men with metabolic syndrome: randomized controlled trial. *J Occup Health* 2012;**54**:215-22.

5. Ferster CB, Nurnberger JI, Levitt EB. The control of eating. 1962. *Obes Res* 1996;**4**:401-10.

6. Hill SW, McCutcheon NB. Eating responses of obese and nonobese humans during dinner meals. *Psychosom Med* 1975;**37**:395-401.

7. Bellack AS. Behavior therapy for weight reduction. *Addict Behav* 1975;**1**:73-82.

8. Otsuka R, Tamakoshi K, Yatsuya H, et al. Eating fast leads to obesity: findings based

For peer review only

For peer review only - http://bmjopen.bmj.com/site/about/guidelines.xhtml

on self-administered questionnaires among middle-aged Japanese men and women. *J Epidemiol* 2006;**16**:117-24.

9. Sasaki S, Katagiri A, Tsuji T, et al. Self-reported rate of eating correlates with body mass index in 18-y-old Japanese women. *Int J Obes Relat Metab Disord* 2003;**27**:1405-10.

10. Maruyama K, Sato S, Ohira T, et al. The joint impact on being overweight of self reported behaviours of eating quickly and eating until full: cross sectional survey. *BMJ* 2008;**337**:a2002.

11. Lee KS, Kim DH, Jang JS, et al. Eating rate is associated with cardiometabolic risk factors in Korean adults. *Nut Metab Cardiovasc Dis* 2013;**23**:635-41.

12. Otsuka R, Tamakoshi K, Yatsuya H, et al. Eating fast leads to insulin resistance: findings in middle-aged Japanese men and women. *Prev Med* 2008;**46**:154-59.

13. Radzeviciene L, Ostrauskas R. Fast eating and the risk of type 2 diabetes mellitus: a case-control study. *Clin Nutr* 2013;**32**:232-35.

14. Ohkuma T, Fujii H, Iwase M, et al. Impact of eating rate on obesity and cardiovascular risk factors according to glucose tolerance status: the Fukuoka Diabetes Registry and the Hisayama Study. *Diabetologia* 2013;**56**:70-77.

15. Shigeta H, Shigeta M, Nakazawa A, et al. Lifestyle, obesity, and insulin resistance.

For peer review only

Diabetes care 2001;**24**:608.

16. Gerace TA, George VA. Predictors of weight increases over 7 years in fire fighters and paramedics. *Prev Med* 1996;**25**:593-600.

17. Tanihara S, Imatoh T, Miyazaki M, et al. Retrospective longitudinal study on the relationship between 8-year weight change and current eating speed. *Appetite* 2011;**57**:179-83.

18. Sakurai M, Nakamura K, Miura K, et al. Self-reported speed of eating and 7-year risk of type 2 diabetes mellitus in middle-aged Japanese men. *Metabolism* 2012;**61**:1566-71.

19. Kral JG, Buckley MC, Kissileff HR, et al. Metabolic correlates of eating behavior in severe obesity. *Int J Obes Relat Metab Disord* 2001;**25**:258-64.

20. Shin A, Lim SY, Sung J, et al. Dietary intake, eating habits, and metabolic syndrome in Korean men. *J Am Diet Assoc* 2009;**109**:633-40.

21. Shen W, Punyanitya M, Chen J, et al. Waist circumference correlates with metabolic syndrome indicators better than percentage fat. *Obesity (Silver Spring)* 2006;**14**:727-36.

22. Alberti KG, Eckel RH, Grundy SM, et al. Harmonizing the metabolic syndrome: a joint interim statement of the International Diabetes Federation Task Force on Epidemiology and Prevention; National Heart, Lung, and Blood Institute; American

For peer review only

Heart Association; World Heart Federation; International Atherosclerosis Society; and International Association for the Study of Obesity. *Circulation* 2009;**120**:1640-5.

23. Ministry of Justice, Japan. Industrial Safety and Health Act, Article 66 (Medical Examination). Available at: http://www.japaneselawtranslation.go.jp/law/detail/?id=1926&vm=&re=. Accessed 28 July 2014.

24. Ministry of Health, Labour and Welfare. Specific Health Checkups and Specific Health Guidance. Secondary Specific Health Checkups and Specific Health Guidance. Available at: http://www.mhlw.go.jp/english/wp/wp-hw3/dl/2-007.pdf. Accessed 28 July 2014.25. The Ministry of Health, Labour and Welfare, Ministry of Education, Culture, Sports, Science and Technology. Ethical guidelines for epidemiological research. 2002. Available at: http://www.niph.go.jp/wadai/ekigakurinri/guidelines.pdf. Accessed 28 July, 2014.

26. Ministry of Health, Labour and Welfare. Questionnaire of Specific Health Checkups. Secondary Questionnaire of Specific Health Checkups. Available at: http://www.mhlw.go.jp/seisakunitsuite/bunya/kenkou_iryou/kenkou/seikatsu/dl/hoken-program2_02.pdf. Accessed 28 July 2014. (in Japanese)

27. Hill SW, McCutcheon NB. Contributions of obesity, gender, hunger, food preference,

For peer review only - http://bmjopen.bmj.com/site/about/guidelines.xhtml

and body size to bite size, bite speed, and rate of eating. *Appetite* 1984;**5**:73-83.

28. Ekuni D, Furuta M, Takeuchi N, et al. Self-reports of eating quickly are related to a decreased number of chews until first swallow, total number of chews, and total duration of chewing in young people. *Arch Oral Biol* 2012;**57**:981-86.

29. Rolls ET. Sensory processing in the brain related to the control of food intake. *Proc Nutr Soc* 2007;**66**:96-112.

30. Morton GJ, Cummings DE, Baskin DG, et al. Central nervous system control of food intake and body weight. *Nature* 2006;**443**:289-95.

31. de Graaf C, Blom WA, Smeets PA, et al. Biomarkers of satiation and satiety. *Am J Clin Nutr* 2004;**79**:946-61.

32. Kokkinos A, le Roux CW, Alexiadou K, et al. Eating slowly increases the postprandial response of the anorexigenic gut hormones, peptide YY and glucagon-like peptide-1. *J Clin Endocrinol Metab* 2010;**95**:333-37.

33. Sobki SH, Zaid AA, Khan HA, et al. Significant impact of pace of eating on serum ghrelin and glucose levels. *Clin Biochem* 2010;**43**:522-24.

34. Viskaal-van Dongen M, Kok FJ, de Graaf C. Eating rate of commonly consumed foods promotes food and energy intake. *Appetite* 2011;**56**:25-31.

35. Scisco JL, Muth ER, Dong Y, et al. Slowing bite-rate reduces energy intake: an

For peer review only

application of the bite counter device. *J Am Diet Assoc* 2011;**111**:1231-35.

36. Mochizuki K, Misaki Y, Miyauchi R, et al. A higher rate of eating is associated with higher circulating interluekin-1beta concentrations in Japanese men not being treated for metabolic diseases. *Nutrition* 2012;**28**:978-83.

37. Besedovsky H, del Rey A, Sorkin E, et al. Immunoregulatory feedback between interleukin-1 and glucocorticoid hormones. *Science* 1986;**233**:652-54

38. Matsuki T, Horai R, Sudo K, et al. IL-1 plays an important role in lipid metabolism by regulating insulin levels under physiological conditions. *J Exp Med* 2003;**198**:877-88.

39. Yatabe MS, Yatabe J, Yoneda M, et al. Salt sensitivity is associated with insulin resistance, sympathetic overactivity, and decreased suppression of circulating renin activity in lean patients with essential hypertension. *Am J Clin Nutr* 2010;**92**:77-82.

40. Facchini FS, DoNascimento C, Reaven GM, et al. Blood pressure, sodium intake, insulin resistance, and urinary nitrate excretion. *Hypertension* 1999;**33**:1008-12.

41. Pollare T, Lithell H, Berne C. Insulin resistance is a characteristic feature of primary hypertension independent of obesity. *Metabolism*. 1990;**39**:167-74.

42. De Vogli R, Kouvonen A, Gimeno D. The influence of market deregulation on fast food consumption and body mass index: a cross national time series analysis. *Bull*

For peer review only - http://bmjopen.bmj.com/site/about/guidelines.xhtml

World Health Organ 2014;**92**:99-107A.

43. Ministry of Health, Labour and Welfare. Special Survey on Industrial Safety and Health. Available at: http://www.mhlw.go.jp/bunya/roudoukijun/anzeneisei11/h23.html. Accessed 28 July 2014. (in Japanese)

For peer review only

STROBE Statement—Checklist of items that should be included in reports of ***cross-sectional studies***

MS ID#: bmjopen-2014-005241.R1
MS TITLE: Self-reported eating rate and metabolic syndrome in Japanese: cross-sectional study

	Item No	Recommendation	Location in manuscript
**Title and abstract**	1	(*a*) Indicate the study's design with a commonly used term in the title or the abstract	Line 1 on page 1 and line 23 on page 3
		(*b*) Provide in the abstract an informative and balanced summary of what was done and what was found	What was done: line 21-22 on page 3<br>What was found: line 42-44 on page 4
**Introduction**			
Background/rationale	2	Explain the scientific background and rationale for the investigation being reported	Scientific background: line 51-67 on page 6<br>Rationale: Line 67-74 on page 6 and 7
Objectives	3	State specific objectives, including any prespecified hypotheses	Line 73-79 on page 7
**Methods**			
Study design	4	Present key elements of study design early in the paper	Line 75 on page 7
Setting	5	Describe the setting, locations, and relevant dates, including periods of recruitment, exposure, follow-up, and data collection	Line 83-89 on page 7-8
Participants	6	(*a*) Give the eligibility criteria, and the sources and methods of selection of participants	Line 89-103 on page 8
Variables	7	Clearly define all outcomes, exposures, predictors, potential confounders, and effect modifiers. Give diagnostic criteria, if applicable	Outcome: line 131-138 on page 10<br>Exposure: line 115 on page 9<br>Potential confounders: line 149-155 on page 11
Data sources/ measurement	8*	For each variable of interest, give sources of data and details of methods of assessment (measurement). Describe comparability of assessment methods if there is more than one group	Line 116-129 on page 9 and 10
Bias	9	Describe any efforts to address potential sources of bias	Exclusion: line 89-103 on page 8<br>Adjustment: line 149-155 on page 11
Study size	10	Explain how the study size was arrived at	Not provided
Quantitative variables	11	Explain how quantitative variables were handled in the analyses. If applicable, describe which groupings were chosen and why	Adjustment: line 149-155 on page 11

For peer review only - http://bmjopen.bmj.com/site/about/guidelines.xhtml

Statistical methods	12	(*a*) Describe all statistical methods, including those used to control for confounding	Line 140-157 on page 11
		(*b*) Describe any methods used to examine subgroups and interactions	Line 155-157 on page 11
		(*c*) Explain how missing data were addressed	We excluded participants who had missing information on potential confounding variables (line 100-102 on page 8).
		(*d*) If applicable, describe analytical methods taking account of sampling strategy	N/A
		(*e*) Describe any sensitivity analyses	N/A
**Results**			
Participants	13*	(a) Report numbers of individuals at each stage of study—eg numbers potentially eligible, examined for eligibility, confirmed eligible, included in the study, completing follow-up, and analysed	Table 1
		(b) Give reasons for non-participation at each stage	N/A
		(c) Consider use of a flow diagram	N/A
Descriptive data	14*	(a) Give characteristics of study participants (eg demographic, clinical, social) and information on exposures and potential confounders	Table 1<br>Line 63-168 on page 12
		(b) Indicate number of participants with missing data for each variable of interest	N/A
Outcome data	15*	Report numbers of outcome events or summary measures	Table 2<br>Line 163 on page 12
Main results	16	(*a*) Give unadjusted estimates and, if applicable, confounder-adjusted estimates and their precision (eg, 95% confidence interval). Make clear which confounders were adjusted for and why they were included	Table 2, Table3<br>Line 169-189 on page 12-13
		(*b*) Report category boundaries when continuous variables were categorized	Table2, Table3
		(*c*) If relevant, consider translating estimates of relative risk into absolute risk for a meaningful time period	N/A
Other analyses	17	Report other analyses done—eg analyses of subgroups and interactions, and sensitivity analyses	Line 155-156 on page 11
**Discussion**			
Key results	18	Summarise key results with reference to study objectives	Line 192-199 on page 13 and 14
Limitations	19	Discuss limitations of the study, taking into account sources of potential bias	Line 245-263 on page 16 and 17

For peer review only - http://bmjopen.bmj.com/site/about/guidelines.xhtml

		or imprecision. Discuss both direction and magnitude of any potential bias	
Interpretation	20	Give a cautious overall interpretation of results considering objectives, limitations, multiplicity of analyses, results from similar studies, and other relevant evidence	Line 200-210 on page 14
Generalisability	21	Discuss the generalisability (external validity) of the study results	Line 254-258 on page 17
**Other information**			
Funding	22	Give the source of funding and the role of the funders for the present study and, if applicable, for the original study on which the present article is based	This study was supported by the Industrial Health Foundation for drafting the maniscript.

N/A: Not applicable.

*Give information separately for exposed and unexposed groups.

Note: An Explanation and Elaboration article discusses each checklist item and gives methodological background and published examples of transparent reporting. The STROBE checklist is best used in conjunction with this article (freely available on the Web sites of PLoS Medicine at http://www.plosmedicine.org/, Annals of Internal Medicine at http://www.annals.org/, and Epidemiology at http://www.epidem.com/). Information on the STROBE Initiative is available at www.strobe-statement.org.

For peer review only
